# Implementation and external validation of the Cambridge Multimorbidity Score in the UK Biobank cohort

**DOI:** 10.1186/s12874-024-02175-9

**Published:** 2024-03-20

**Authors:** Hannah Harrison, Samantha Ip, Cristina Renzi, Yangfan Li, Matthew Barclay, Juliet Usher-Smith, Georgios Lyratzopoulos, Angela Wood, Antonis C. Antoniou

**Affiliations:** 1 Department of Public Health and Primary Care, School of Clinical Medicine, University of Cambridge, Cambridge, UK; 2 Victor Phillip Dahdaleh Heart and Lung Research Institute, University of Cambridge, Cambridge, UK; 3 Department of Behavioural Science and Health, Institute of Epidemiology and Healthcare, University College London, London, UK; 4 Faculty of Medicine, University Vita-Salute San Raffaele, Milan, Via Olgettina 58, Milan, Italy; 5 National Institute for Health and Care Research Blood and Transplant Research Unit in Donor Health and Behaviour, University of Cambridge, Cambridge, UK; 6 Health Data Research UK Cambridge, Wellcome Genome Campus and University of Cambridge, Cambridge, UK

**Keywords:** Multimorbidity, UK Biobank, External validation, Electronic health records, Primary care records

## Abstract

**Background:**

Patients with multiple conditions present a growing challenge for healthcare provision. Measures of multimorbidity may support clinical management, healthcare resource allocation and accounting for the health of participants in purpose-designed cohorts. The recently developed Cambridge Multimorbidity scores (CMS) have the potential to achieve these aims using primary care records, however, they have not yet been validated outside of their development cohort.

**Methods:**

The CMS, developed in the Clinical Research Practice Dataset (CPRD), were validated in UK Biobank participants whose data is not available in CPRD (the cohort used for CMS development) with available primary care records (n = 111,898). This required mapping of the 37 pre-existing conditions used in the CMS to the coding frameworks used by UK Biobank data providers. We used calibration plots and measures of discrimination to validate the CMS for two of the three outcomes used in the development study (death and primary care consultation rate) and explored variation by age and sex. We also examined the predictive ability of the CMS for the outcome of cancer diagnosis. The results were compared to an unweighted count score of the 37 pre-existing conditions.

**Results:**

For all three outcomes considered, the CMS were poorly calibrated in UK Biobank. We observed a similar discriminative ability for the outcome of primary care consultation rate to that reported in the development study (C-index: 0.67 (95%CI:0.66–0.68) for both, 5-year follow-up); however, we report lower discrimination for the outcome of death than the development study (0.69 (0.68–0.70) and 0.89 (0.88–0.90) respectively). Discrimination for cancer diagnosis was adequate (0.64 (0.63–0.65)). The CMS performs favourably to the unweighted count score for death, but not for the outcomes of primary care consultation rate or cancer diagnosis.

**Conclusions:**

In the UK Biobank, CMS discriminates reasonably for the outcomes of death, primary care consultation rate and cancer diagnosis and may be a valuable resource for clinicians, public health professionals and data scientists. However, recalibration will be required to make accurate predictions when cohort composition and risk levels differ substantially from the development cohort. The generated resources (including codelists for the conditions and code for CMS implementation in UK Biobank) are available online.

**Supplementary Information:**

The online version contains supplementary material available at 10.1186/s12874-024-02175-9.

## Background

The prevalence of multimorbidity (living with two or more medical conditions) is rising; in higher income countries this is largely driven by aging populations [1], and it is more likely to affect people from deprived areas or with lower socioeconomic status [2]. In England nearly 15 million people are multimorbid, and this is predicted to rise significantly in the next 10 years [3]. Around half of all clinical interactions involve patients with multiple health conditions, and multimorbidity is expected to continue driving increased demand for healthcare resources [3].

The rise in multimorbidity presents challenges for the clinical management of individuals with complex medical history, and for effective use of the resources within the healthcare system [3, 4]. The Academy of Medical Sciences identified research into multimorbidity as a global health priority, highlighting evidence gaps around the prevalence of multimorbidity and the effectiveness of existing healthcare services for this population [5]. A recent review, however, found that measurement of multimorbidity is highly variable and often poorly reported [6]. For researchers, including public health and primary care professionals, there is an increasing need to quantify the level of multimorbidity in populations of interest, and to project the expected outcomes and healthcare resource use of this group.

Many existing scores can be used to assess disease burden in individuals within a population. Examples include the Eastern Cooperative Oncology Group performance status [7], and the Charlson comorbidity score [8]. In a recent paper, Payne et al. [9] developed the Cambridge multimorbidity scores (CMS) which use common pre-existing conditions to predict death, hospital admission and primary care consultation rate at an individual level. Unlike previous scores, the CMS were developed specifically for use with primary care electronic health records. These design features mean that the CMS are also of interest to researchers working with large primary care datasets; they provide a method to assess the health status of the population of interest, or to adjust for health status when modelling other associations.

The CMS were developed and internally validated in the Clinical Research Practice Dataset [9]. In this study, we implemented the CMS in participants of the UK Biobank cohort with available primary care records, requiring adaption for use with a range of coding frameworks used in UK primary care. We externally validated the CMS for two of the three outcomes that were used in the development study (death and primary care consultation rate) and explored variation in performance by demographic characteristics (age and sex). In addition, we also examined the predictive ability of the CMS for the outcome of cancer diagnosis, motivated by a need for an appropriate measure of multimorbidity as an effect modifier of cancer risk [10].

## Methods

### Study population

UK Biobank is a large population-based cohort of 502,619 participants aged 40–69 at the time of enrolment (between 2006 and 2010, [11]. Data on deaths and cancer incidence are available through linkage to national registries. Primary care data have been linked and are available for about half of the cohort (n = 228,913), which includes coded information from primary care consultations (symptoms, diagnoses, and test results) and prescription records.

**Fig. 1:**
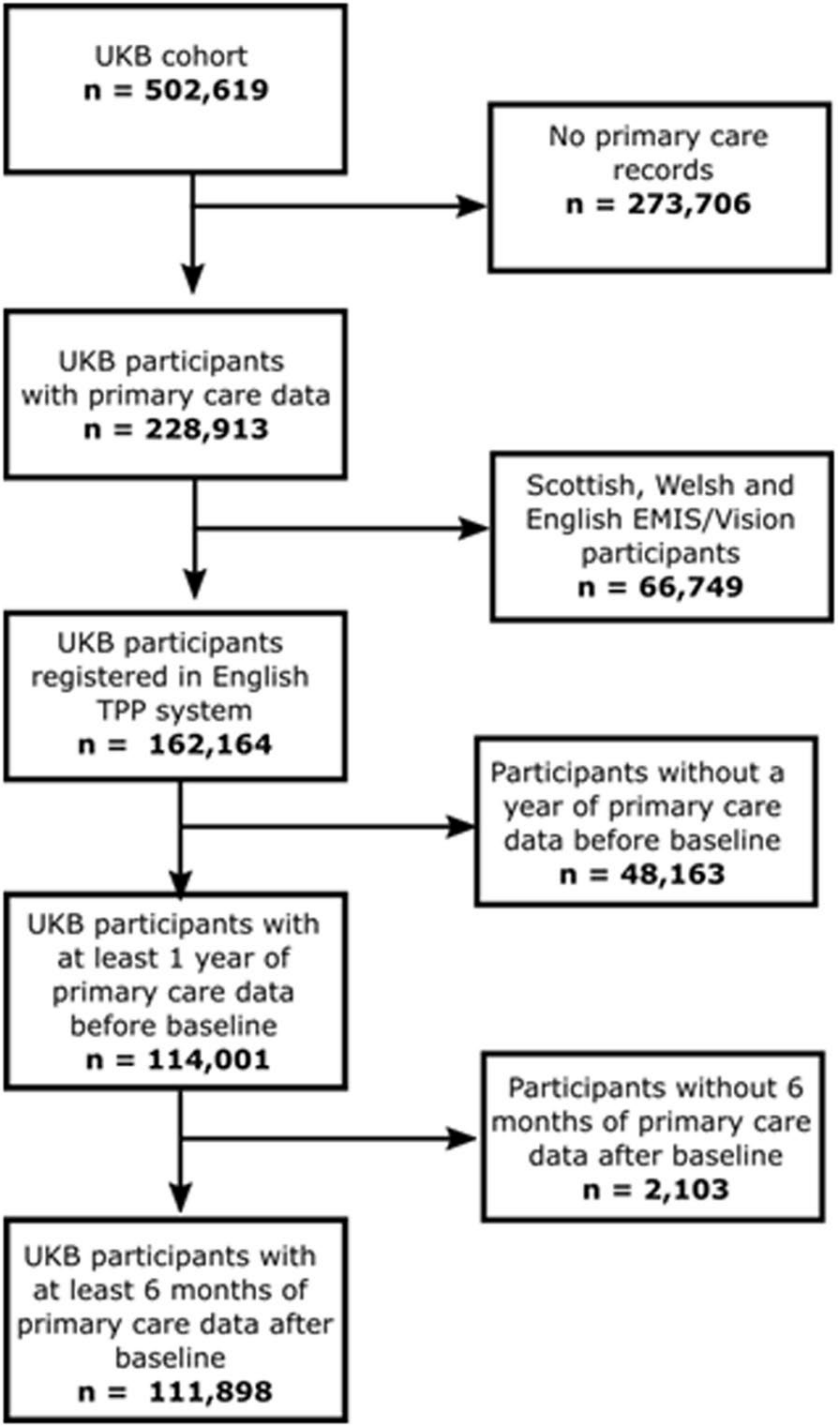
Cohort flow diagram

The CMS were implemented for the whole primary care cohort; however, validation was restricted to participants who were registered at one of the four data providers (English TPP, see Table S 7), to avoid potential crossover with the development population (Clinical Practice Research Datalink) used by Payne et al. [9]. Individuals included in the validation were also required to have at least one year of continuous primary care data (defined as no gaps in registration > 90 days) prior to baseline assessment and six months afterwards. Thus, the validation cohort included 111,898 individuals (Fig. 1).

### Outcomes

The performance of the score was assessed for two of the three outcomes used in the development study: death and primary care consultation rate. Additionally, we investigated the performance of the scores for the outcome of cancer diagnosis. All three outcomes were determined during follow-up, between the date of baseline assessment for each individual and the study end date (10/07/2016, the last valid data entry for the available English TPP data). Deaths were determined using linked death registry data. Primary care consultation rate was determined by calculating the number of primary care clinical records available for each cohort member (ignoring multiple records on the same day) and dividing by the available follow-up period, as in the development study [9]. Cancer incidence was determined via linkage to the cancer registry, we identified the first diagnosis of cancer (excluding non-melanoma skin cancer) recorded after baseline assessment. Note that the main analyses included individuals with a diagnosis of cancer before UK Biobank baseline assessment; this was done as patients with a prior cancer are also at risk of subsequent cancers, reflecting that cancer diagnosis is both a prior condition included in the CMS and an outcome in the analysis. A sensitivity analysis was carried out for the cancer diagnosis outcome which removed individuals with cancer diagnosis before baseline assessment from the analysis.

### Implementation of the Cambridge multimorbidity score

Codelists, used to define the 37 conditions included in the multimorbidity score for the Clinical Practice Research Datalink cohort (as used in the development study [9]), are freely available online [12]. These were mapped onto the coding frameworks used by the UK Biobank primary care data providers, including Clinical Terms Version 3 (CTV3) read codes for consultation data and the British National Formulary (BNF) codes and Dictionary of Medicines and Devices (DM + D) codes for prescription data (see Table S7). Look-up tables provided by both Clinical Practice Research Datalink and UK Biobank were used to identify equivalent codes in each framework. After mapping, manual checking was used to minimise inconsistencies and identify any erroneously included codes. Further details are given in the supplementary methods.

The mapped codelists were used to query clinical and prescription records for UK Biobank participants with primary care records. The definitions used for the 37 conditions (as defined by the CPRD@Cambridge collaboration [12]) vary between conditions, from the presence of an event at any point in time prior to the index date (includes diabetes and hypertension), to checking for regular prescriptions (includes migraine) or checking the level of the last two recorded glomerular filtration rate tests (chronic kidney disease). A list of the 37 conditions is given in Table S1.

Once the conditions had been defined, the multimorbidity scores were calculated using the date of the UK Biobank baseline assessment as the index date. Three versions of the CMS developed by Payne et al. [9] were considered: (1) the general outcome score, a weighted linear sum of the conditions; (2) a Cox proportional hazards model originally developed for the outcome of mortality; and (3) a Cox proportional hazards model originally developed for the outcome of hospital admission. They are hereafter referred to as the general CMS, mortality CMS and the hospital CMS respectively. The mortality CMS and hospital CMS include variables for age and sex in addition to the conditions. All three include all 37 conditions, however, we also implemented and validated the short versions of the models for the mortality and hospital CMS (also developed by Payne et al. [9]), which include only the 20 most influential conditions (Table S1).

An unweighted count score (a sum of the number of prevalent conditions for each individual) was also included in the analysis (see Table 1) – this was not used in the development study and is included here as a comparator for the CMS. We validated all scores for the three outcomes (death, GP consultation rate and cancer diagnosis), to identify to what extent the scores are good predictors of poor health outcomes in general, and the extent to which they specifically predict the outcomes for which they were designed.

**Table 1:** Scores included in analysis

Models	From development study (Payne et al. [9])	Model Type	Included Variables	Model outcome (in development study)	Outcomes assessed in UKB validation	Alternate versions
General CMS	Y	Weighted linear sum of the conditions	37 conditions	Weighted combination of death, hospital admission and GP consultation rate	Death, GP consultation rate, cancer diagnosis	-
Mortality CMS	Y	Cox proportional hazards (prognostic index used in validation)	37 conditions, age, sex	Death in follow-up	Death, GP consultation rate, cancer diagnosis	Results for short version with 20 conditions in supplementary materials
Hospital CMS	Y	Cox proportional hazards (prognostic index used in validation)	37 conditions, age, sex	Emergency hospital admission in follow-up	Death, GP consultation rate, cancer diagnosis	Results for short version with 20 conditions in supplementary materials
Unweighted Count Score	N	Sum of number of conditions	37 conditions	-	Death, GP consultation rate, cancer diagnosis	Results for short version with 20 conditions in supplementary materials

### Assessing performance

The discrimination of the implemented scores was assessed by calculating the concordance statistic – equivalent to Harrell’s C-index for the binary outcomes (death and cancer diagnosis) – to compute agreement between the scores and the outcomes [13]. We used the “concordance” function in the R survival package [14].

To assess the discrimination over a range of follow-up periods, we calculated the concordance statistic for a range of follow-up periods (1–10 years) after baseline assessment. Concordance (or C-statistic) is a rank order statistic which computes the probability that a randomly chosen individual with the outcome has a higher score than a randomly chosen individual without the outcome. A C-statistic of 0.5 indicates no discriminatory ability and that the score is no better than a random coin toss at distinguishing between these two individuals, while a C-statistic of 1 indicates perfect discrimination. Individuals were censored after an event occurred (death or cancer incidence), at death, at the study end date (last date of linkage for the primary care records) or at the end of the follow-up period (ranging from 1–10 years). When measuring discrimination for the outcome of GP consultation rate, we only included cohort members with available GP records up to the end of each follow-up period (for example, inclusion in the analysis for 5-years of follow-up required continuous registration for 5-years after baseline). Subgroup analysis was used to examine differences in discrimination by sex and 10-year age group (general CMS only). Calibration of the CMS (for all outcomes) was assessed graphically [15], using calibration plots comparing the risk predicted by the CMS and the observed relative risk of the outcome for each decile of the CMS (more details in supplementary methods).

All analysis (including codelist mapping, implementation of the CMS and assessing performance) was conducted in R (version 4.1.0).

## Results

### Validation cohort

The validation cohort (n = 111,898) had similar demographic characteristics to the full UK Biobank cohort and the subset with linked primary care records (Table S2); the mean age (sd) in the validation cohort is 57.0 (8.0) years compared to 56.5 (8.1) and 56.7 (8.1) years respectively; and the percentage of women is 53.7% compared to 54.4% and 54.5% respectively.. In the validation cohort 54,579 (48.8%) participants had at least one of the 37 conditions at baseline, and 25,738 (23.0%) had two or more conditions. Participants with one or more conditions were more likely to be women, smokers, overweight and live in an area with high levels of deprivation than those with no conditions (Table 2). The most common conditions identified in the validation cohort were painful condition (16.8%), hypertension (16.5%) and anxiety/depression (9.7%). Some of the conditions were very rare in this cohort, for example, only 17 people had dementia at baseline. Prevalence of all 37 conditions in the validation cohort at baseline assessment is given in Table S1.

**Table 2:** Characteristics of Validation Cohort

		**Whole cohort**	**No conditions**	**1 condition**	** > 1 condition**
**Counts**	**n (%)**	111,898 (100)	57,319 (51.2)	28,841 (25.8)	25,738 (23.0)
**Cohort Characteristics**					
**Age**	**Mean (SD)**	57.0 (8.0)	55.0 (8.0)	58.0 (7.6)	60.0 (7.0)
	**Missing (%)**	0 (0)	0 (0)	0 (0)	0 (0)
** Sex**	**Female (%)**	60,036 (53.7)	30,165 (52.6)	15,557 (53.9)	14,314 (55.6)
	**Male (%)**	51,862 (46.3)	27,154 (47.4)	13,284 (46.1)	11,424 (44.4)
	**Missing (%)**	0 (0)	0 (0)	0 (0)	0 (0)
** Smoking Status**	**Never (%)**	61,638 (55.1)	33,956 (59.2)	15,402 (53.4)	12,280 (47.7)
	**Former (%)**	38,845 (34.7)	17,669 (30.8)	10,615 (36.8)	10,561 (41.0)
	**Current (%)**	10,864 (9.7)	5475 (9.5)	2678 (9.3)	2711 (10.5)
	**Missing (%)**	551 (0.49)	219 (0.38)	146 (0.51)	186 (0.72)
** Deprivation Quintiles (English IMD)**	**1 (least deprived)**	29,789 (26.6)	16,323 (28.5)	7644 (26.5)	5822 (22.6)
	**2**	26,247 (23.5)	13,893 (24.2)	6724 (23.3)	5630 (21.9)
	**3**	22,008 (19.7)	11,224 (19.6)	5718 (19.8)	5066 (19.7)
	**4**	18,082 (16.2)	8735 (15.2)	4780 (16.6)	4567 (17.7)
	**5 (most deprived)**	12,460 (11.1)	5447 (9.5)	3141 (10.9)	3872 (15.0)
	**Missing (%)**	3312 (3.0)	1697 (3.0)	834 (2.9)	781 (3.0)
** BMI**	**Mean (sd)**	27.4 (4.7)	26.6 (4.2)	27.7 (4.7)	29.0 (5.3)
	** < 20 (%)**	2444 (2.2)	1470 (2.6)	577 (2.0)	397 (1.5)
	**20—24.9 (%)**	33,265 (29.7)	20,266 (35.4)	7780 (27.0)	5219 (20.3)
	**25—29.9 (%)**	46,958 (42.0)	24,310 (42.4)	12,490 (43.3)	10,158 (39.5)
	** ≥ 30 (%)**	26,349 (23.5)	9924 (17.3)	7271 (25.2)	9154 (35.6)
	**Missing (%)**	2882 (2.6)	1349 (2.4)	723 (2.5)	810 (3.1)
**Outcome measures**					
** Deaths**	**Number in 1 year (%)**	161 (0.14)	42 (0.07)	29 (0.10)	90 (0.35)
	**Number in 5 years (%)**	1987 (1.77)	587 (1.02)	522 (1.81)	878 (3.41)
** Cancer Diagnoses**	**Number in 1 year (%)**	1071 (0.96)	432 (0.75)	307 (1.06)	332 (1.29)
	**Number in 5 years (%)**	5589 (5.0)	2377 (4.15)	1565 (5.43)	1647 (6.40)
** GP Consultation Rate**	**Rate per year, Median (IQR)**	7.68 (4.78–11.73)	5.54 (3.53–8.34)	8.77 (6.19–12.2)	12.46 (8.95–17.6)
	**Zero consultations (%)**	43 (0.04)	28 (0.05)	7 (0.02)	8 (0.03)
** GP Follow-up (years)**	**Median (IQR)**	7.03 (6.48–7.61)	7.05 (6.56–7.62)	7.02 (6.48–7.59)	7.00 (6.41–7.57)

Over a ten-year follow-up period, 5,503 deaths and 8,315 cancer diagnoses were recorded in the validation cohort (Fig. S1). The likelihood of death or cancer diagnosis was higher in those with one or more conditions at baseline compared to those with no conditions at baseline (Table 2); 2.6 times higher for death and 1.4 times higher for cancer diagnosis after 5 years of follow-up.

Consultation rate was analysed up to 8 years after baseline (only 10,219 members of the validation cohort had 8 continuous years of primary care records from baseline). The median number of primary care consultations per year was 7.68 (IQR: 4.78–11.73) and only 43 cohort members had no consultations recorded (Table 2). Cohort members with one or more conditions at baseline had higher consultation rates than those without (median (IQR): 10.4 (7.23–14.8) and 5.54 (3.53–8.34) respectively).

### Predicting mortality

**Fig. 2:**
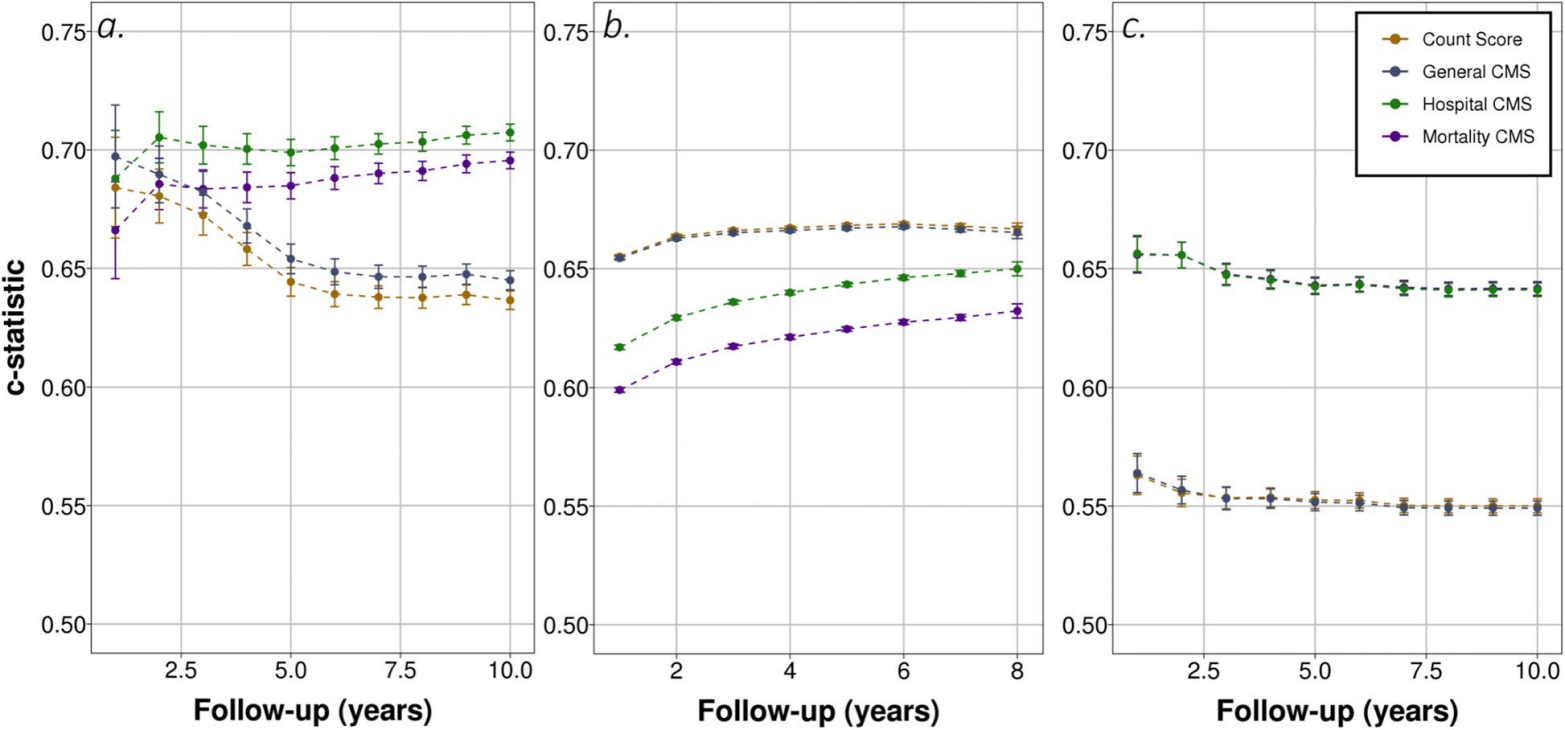
Discrimination of models over time for (**a**) death, (**b**) primary care consultation rate and (**c**) cancer diagnosis

As shown in Fig. 2(a) and Table S3, the general CMS had a C-index of 0.70 (95% CI: 0.68–0.72) for the outcome of mortality in the first year of follow-up. The C-index decreased for 5-year risk prediction to 0.65 (95% CI: 0.64–0.66) and remained constant up to 10-years of follow-up. The general CMS discriminated slightly better than the unweighted count score for all follow-up periods. The mortality CMS and hospital CMS had similar concordance to the general CMS for short term follow-up (mortality CMS and hospital CMS, 1-year: 0.67 (95% CI: 0.65–0.69) and 0.69 (95% CI: 0.67–0.71) respectively) but over longer follow-up periods they showed better discriminatory ability (mortality and hospital CMS, 5-years: 0.69 (95% CI: 0.68–0.69) and 0.70 (95% CI: 0.69–0.71) respectively).

The general CMS was poorly calibrated for mortality, with some underestimation of risk in the lower deciles and over estimation in higher deciles for both 1 and 5- year follow-up (Fig. S3). Both the mortality CMS and hospital CMS also show poor calibration, significantly underestimating the relative risk to those in the higher deciles. In contrast the unweighted count score appears to be well calibrated for mortality, especially for shorter follow-up periods (1-year).

In subgroup analysis (Figs S2(a-c), Table S5(a)), the general CMS had better discriminatory ability for men (5-year: 0.67 (95% CI: 0.66–0.68)) compared to women (5-year: 0.63 (95% CI: 0.62–0.64)). The highest concordance values for most follow-up periods were for younger (in their 40’s at baseline) men (5-year: 0.68 (95% CI: 0.65–0.71)). Conversely, the score was least discriminatory in younger women (5-year: 0.57 (0.53–0.61)).

### Predicting primary care consultation rate

For primary care consultation rate (Fig. 2(b) and Table S3), the general CMS had a concordance of 0.65 (95% CI: 0.64–0.66) for a follow-up period of 1-year, then remained relatively stable (5-year follow-up period: 0.67 (95% CI: 0.66–0.68)). The unweighted count score had a similar discriminatory performance (5-year follow-up period: 0.67 (95% CI: 0.66–0.67)) whilst the concordance measured for the mortality CMS and hospital CMS is lower (5-year follow-up period: 0.63 (95% CI: 0.62–0.64) and 0.64 (95% CI: 0.64–0.65) respectively).

None of the models were well calibrated for this outcome (Fig. S3), with the general CMS and the unweighted count score in particular showing underestimation of risk in the lower deciles and overestimation in the higher deciles. The mortality CMS and hospital CMS were better calibrated for this outcome, but underestimated risk in higher deciles.

In subgroup analysis (Figs S2(d-f), Table S5(b)), the discriminatory ability of the general CMS was similar for men and women. Additionally, for both men and women, concordance was higher for older (in their 60’s at baseline) than younger (in their 40’s at baseline) individuals.

### Predicting cancer diagnosis

Discriminatory ability of all the scores included in the analysis was much lower when predicting cancer diagnosis, compared to the other outcomes measured in this study (Fig. 2(c) and Table S3). The general CMS had a C-index < 0.6 across all prediction time-horizons considered (5-year: 0.55 (95% CI: 0.54–0.56)) and very similar performance to the unweighted count score. Both the mortality CMS and the hospital CMS showed adequate discriminatory ability (mortality CMS and hospital CMS, 5-years: 0.64 (0.63–0.65) and 0.64 (95% CI:0.63–0.65) respectively).

All models had very poor calibration for this outcome. The calibration plots (Fig. S3) showed very little variation in the observed outcome between the deciles for the general CMS and unweighted count score. Conversely, the mortality CMS and hospital CMS significantly overestimate the risk for those in lower deciles and underestimate the risk to those in the higher deciles.

In subgroup analysis by age and sex (Figs S2(g-i), Table S5(c)), the general CMS had a higher discriminatory power for men (5-year: 0.56 (0.55–0.57)) and lower for women (5-year: 0.54 (0.53–0.55)). A sensitivity analyses which measured the discrimination for this outcome in a cohort excluding any individuals diagnosed with cancer (except non-melanoma skin cancer) before baseline assessment (n = 103,463) found no significant differences compared to the main analyses (Fig. S4).

### Additional analyses

Results examining short versions of the models (with only 20 conditions) for all three outcomes are found in the supplementary materials (Table S4); no significant differences with the primary results are seen.

## Discussion

### Summary

This is the first implementation and external validation of the CMS in a population distinct to the development cohort. The scores have been implemented for the four primary care data providers that supplied data to UK Biobank. In validation, the general CMS had reasonable discrimination for the outcomes of death, primary care consultation rate and cancer diagnosis in this population; however, this was comparable to the discriminatory ability of an unweighted count score for the outcomes of primary care consultation rate and cancer diagnosis. Calibration was generally poor across all the scores and outcomes tested. 

### Results in context

The discrimination measured in the UKB validation cohort was lower than that measured in the development cohort (Clinical Practice Research Datalink) by Payne et al. [9] for most combinations of outcome and score measured in both studies. The discrimination (C-index) of the general CMS in the development cohort was 0.82 (95% CI:0.81–0.83) for the outcome of mortality over a 5-year follow-up period compared to 0.65 (95% CI:0.64–0.67) in this UK Biobank validation (Table 3). However, the general CMS has a very similar performance for the outcome of primary care consultation rate in both development and this validation, for a five-year follow-up period both report a concordance statistic of 0.67. The modest performance of the CMS in the UKB cohort –with comparable discrimination to an unweighted count score – makes a weak case for using the CMS in isolation to predict negative health outcomes outside of the development cohort. However, this does not mean that the CMS does not capture useful information about the underlying health status of individuals in a cohort, and it may be a useful variable to include in analyses when examining associations or developing prognostic models for a range of outcomes. Further, we note that we did not measure the performance of the CMS for one of the outcomes used in the development study (hospital admission).

**Table 3:** Comparison of discrimination (c-statistic) between development study and validation, 5-year follow-up

	**Outcomes (5 -year)**		
	**Death**	**Cancer Diagnosis**	**Rate**
**a: Validation (UKB)**			
**General CMS**	0.654 (0.648–0.660)	0.552 (0.548–0.555)	0.667 (0.666–0.668)
**Mortality CMS**	0.685 (0.679–0.690)	0.643 (0.64–0.646)	0.625 (0.624–0.625)
**Hospital CMS**	0.699 (0.693–0.704)	0.643 (0.639–0.646)	0.643 (0.642–0.644)
**Unweighted Count Score**	0.644 (0.638–0.0.650)	0.553 (0.549–0.556)	0.668 (0.668–0.669)
**b: Development (CPRD), from Payne et al. (2020)**			
**General CMS**	0.824 (0.819–0.830)	**-**	0.667 (0.665–0.668)
**Mortality CMS**	0.890 (0.886–0.894)	-	-
**Hospital CMS**	-	-	-
**Unweighted Count Score**	-	-	-

The calibration of all of scores was poor for most outcomes. This suggests that while the discrimination of these scores was adequate, they may be a poor fit for accurately predicting the likelihood of these outcomes in the UK Biobank cohort. Recalibration of the multimorbidity score within the intended population of use, may be required to make accurate predictions of the level of risk for individuals for these three outcomes.

A recently published study by Tsang et al. [16] developed a modified version of the CMS (developing their own score using the same 37 pre-existing conditions) using data from the English primary care sentinel surveillance network, but did not implement or validate the original CMS developed by Payne et al. [9]. Their new model had excellent discrimination for the outcome of death for both short term (C-index of 0.92 at 1 year) and long term (C-index of 0.91 at 5 years) follow-up in internal validation (also using the sentinel surveillance network cohort), which is in line with the performance of the CMS in its development cohort, but higher than the results we have seen in this external validation of the CMS in UK Biobank. Another recent study tested the performance of the CMS for cancer diagnosis (but not death or GP consultation) rate using self-reported information on the 37 pre-existing conditions from the UKB baseline assessment [17]. This study found no clear association between the CMS and breast, colorectal and prostate cancer diagnosis over a long follow-up period (10 years) – however, those with a high number of pre-existing conditions (≥ 4) were found to be two times more likely to be diagnosed with lung cancer.

### Limitations

Differences between the development cohort and UK Biobank should be considered when interpreting these results. The cohort used in the development study included adults aged 21–95 years, while UK Biobank only recruited individuals aged 40–70 years. Further, UK Biobank is known to be significantly different from the general population of the UK, including differences in demographics and health outcomes [18].

The development cohort and the UKB validation cohort also have substantial differences in prevalence for the 37 conditions included in the CMS (Table S1). For most conditions (33/37) the prevalence is lower in the UK Biobank cohort (for example, chronic kidney disease is nearly twice as prevalent in the development cohort than the UK Biobank validation cohort: 4.5% and 2.5% respectively) reflecting the established healthy volunteer bias in UK Biobank [18]. Individuals with some conditions (for example, learning disability or dementia) are likely to have been deemed ineligible for the UK Biobank cohort due to concerns around consent. There are a small number of conditions that are more common in the UK Biobank cohort, including the most common “painful condition” (16.8%), which is only the third most common condition in the CPRD development cohort (11.6%), and “irritable bowel syndrome” (6.1% and 7.6% respectively). These differences may be explained by the difference in age range between the cohorts, and differences in health-seeking behaviours between the UK Biobank cohort and the general population. Additional analysis of the CMS in a restricted subset of the Clinical Practice Research Datalink dataset with characteristics similar to the UK Biobank cohort (for example by limiting ages to the range 40–70 years) could improve understanding how differences in the performance of the score in these two cohorts is driven by the differences in the composition of the two datasets.

In both this study and the development study [9], primary care consultation rate is derived by identifying the number of entries recorded by the primary care provider for each individual in the follow-up period. To avoid inflating the number of consultations, successive events on the same day are not counted, however, this measure does include other interactions with primary care that are not consultations. These include failed attempts to contact a patient, the input of test results or a record of events in secondary care that have been reported to the primary care provider of the patient. While this outcome may be a good indicator of the level of interaction an individual has with their primary care provider, it is not necessarily a precise estimate of the number of consultations. In this study, we measure an average rate of 7.7 consultations per year in UK Biobank, this is slightly higher than the rate seen in the development study (5.9/year), likely due to the higher average age of the UK Biobank cohort.

### Applications of the CMS

The CMS may be a useful tool for data scientists, public health professionals and primary care clinicians looking to assess the health of a population for which primary care electronic health records are available. It also provides a method to account for variation in health status when examining associations or developing prognostic models in this type of data. The resources developed as part of this work will support these applications.

Part of the motivation for this analysis was to identify a suitable method to account for multimorbidity when investigating the risk of a cancer diagnosis in the UK Biobank cohort. The relationship between multimorbidity and a cancer diagnosis is complex [19]; in a recent study, looking at colorectal cancer diagnoses, patients with multiple conditions were found to be more likely to consult their primary care provider with relevant symptoms, yet also more likely to be diagnosed following emergency presentation [10, 20]. By including a version of the CMS as a variable in a prognostic model for cancer risk, it is possible to capture information about the multimorbidity and the relative burden of the 37 conditions in the model, without including variables for each individual condition. Note that in this study, we examined the performance of the CMS for a diagnosis of any cancer after baseline assessment (excluding non-melanoma skin cancer); in practice, the association of the score with a diagnosis is likely to vary between cancer types. Further, the inclusion of people in this analysis with diagnoses of cancer before baseline means that this outcome many include a small number of secondary cancer diagnoses. However, a sensitivity analysis restricting the validation cohort to individuals without a diagnosis of cancer before UK Biobank baseline assessment (hence including only primary diagnoses in the outcome) found minimal change in the measured discrimination (Fig. S4).

For potential users, understanding the extent to which the performance of the CMS is driven by the complex interaction between aging, multimorbidity and the outcomes of interest will be key. In this study, we showed that the general CMS (which does not include age explicitly as a variable) discriminates well for the outcomes of death and primary care consultation rate. The discriminatory ability of this score is only slightly reduced when validated separately for different age groups. It is unsurprising that the mortality CMS and hospital CMS, which explicitly include terms for age and sex in addition to the 37 conditions, perform significantly better for predicting death and cancer diagnosis. However, it is notable that these models do not outperform the general CMS (which does not include terms of age and sex) when predicting primary care consultation rate. This may simply reflect that the mortality CMS and hospital CMS are not suitable for predicting this outcome, however, it may also suggest that primary care use is influenced more by multimorbidity (the number of conditions) than age.

### Available resources

A key facet of this work has been the implementation of the CMS in a new cohort and the development of resources which may be of interest to readers working with electronic health records, in particular, primary care data from UK data providers. The four data providers which supplied records to UK Biobank cover 36% of primary care practices in England [21] and 100% of the primary care practices in Scotland [22] and Wales [23]. All of the resources developed are avaliable in a public GitHub repository (https://github.com/CCGE-Cambridge/ACED-multimorbidity) and include:

(i) Code to automate conversion of codelists developed for CPRD Gold (Readv2 and Prodcodes) to the coding frameworks used in UK Biobank (including CTV3, BNF and D + MD)
(ii) Codelists for the 37 conditions defined by Payne et al. [9] suitable for use with the four data providers linked to UK Biobank
(iii) Code to implement the CMS in UK Biobank
(iv) Analysis code for the validation (cohort definition, outcome definition and performance measurement)

As all the primary care data linked to the UK Biobank cohort is from before 2018, we have not developed or used codelists based on the SNOMED framework. However, SNOMED codelists for the 37 conditions used in the CMS have recently been published [16], providing a complimentary resource to (ii).

## Conclusion

The CMS has reasonable discrimination for predicting death, primary care consultation rate and cancer diagnosis in an external validation using the UK Biobank (a cohort that is different from the model development cohort). This suggests the CMS may be a valuable resource for clinicians looking to predict short and long-term health outcomes for patients with multimorbidity, as well as a useful resource for data scientists and public health professionals looking to quantify the overall health of a population cohort or to adjust for multimorbidity when investigating other health conditions (such as cancer). Calibration of the CMS for all the outcomes considered in this analysis is poor and their discriminatory ability is comparable to that of an unweighted count score; recalibration may be required when using in cohorts that differ substantially from the development cohort.

### Supplementary Information

**Supplementary material 1.**

**Supplementary material 2.**

**Supplementary material 3.**

## Data Availability

This paper uses data from UK Biobank that the authors do not have permission to distribute. Bona-fide researchers can apply for access to this data, including linked primary care records, from UK Biobank https://www.ukbiobank.ac.uk/
