# Supplementary material for: Implementation and external validation of the Cambridge Multimorbidity Score in the UK Biobank cohort
Source: BMC Med Res Methodol. 2024 Mar 20;24:71. doi: 10.1186/s12874-024-02175-9 (PMC10953059; doi:10.1186/s12874-024-02175-9)
Supplement: Supplementary file 1 — Supplementary material 1. [file 12874_2024_2175_MOESM1_ESM.docx]

Figure S1 – Kaplan-Meier Plots for the outcomes of (a) death and (b) cancer diagnosis.

These Kaplan-Meier plots show 1 - probability of the outcomes of (a) death and (b) a cancer diagnosis for the cohort over the 10-year follow-up period after baseline. At baseline (no follow-up) there is a 0% likelihood of the outcomes, while after ten years there is a 4% chance of death and a 9% likelihood of a cancer diagnosis. The number of people in the cohort and the number of events (cumulative) are indicated in 2.5 year interval underneath each plot.


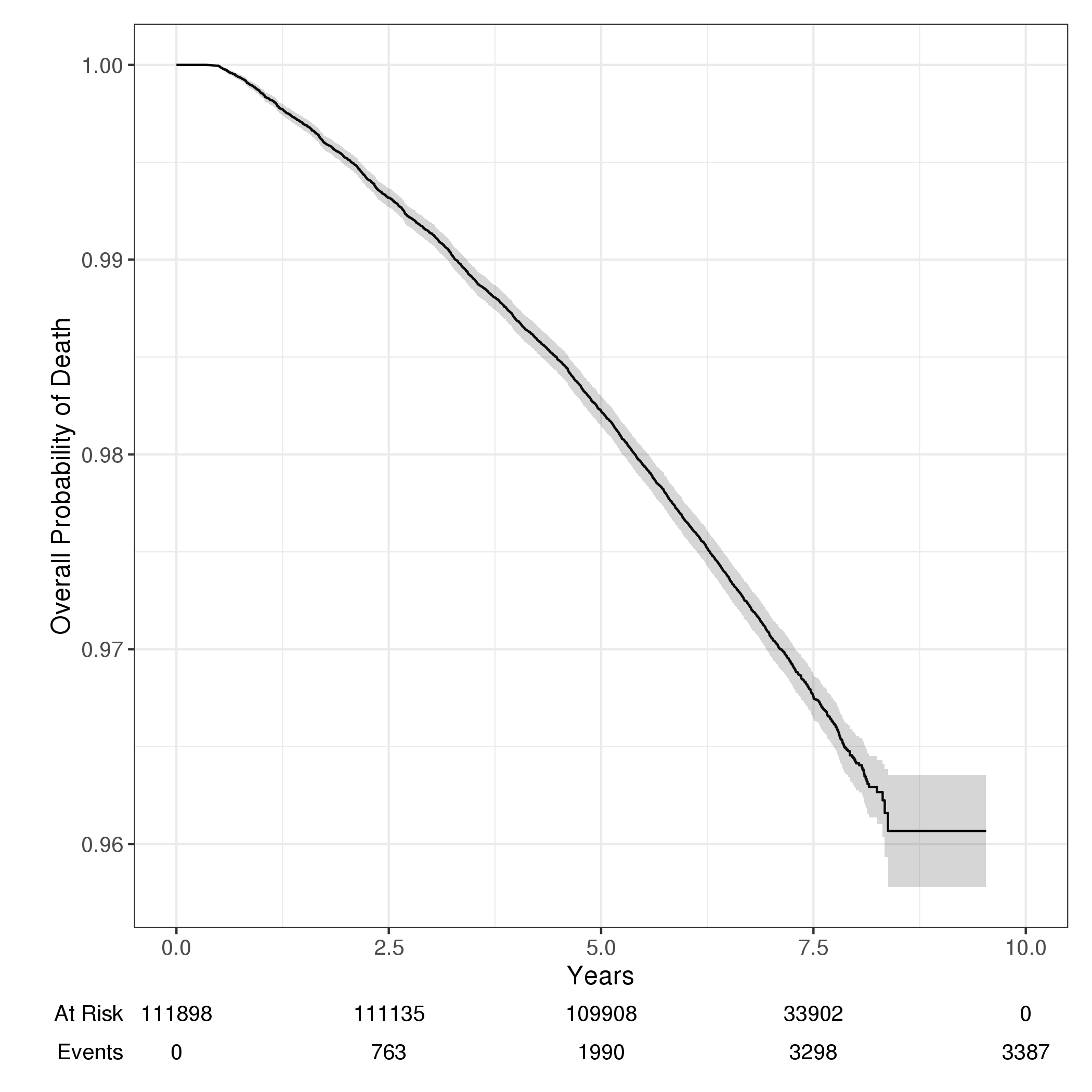

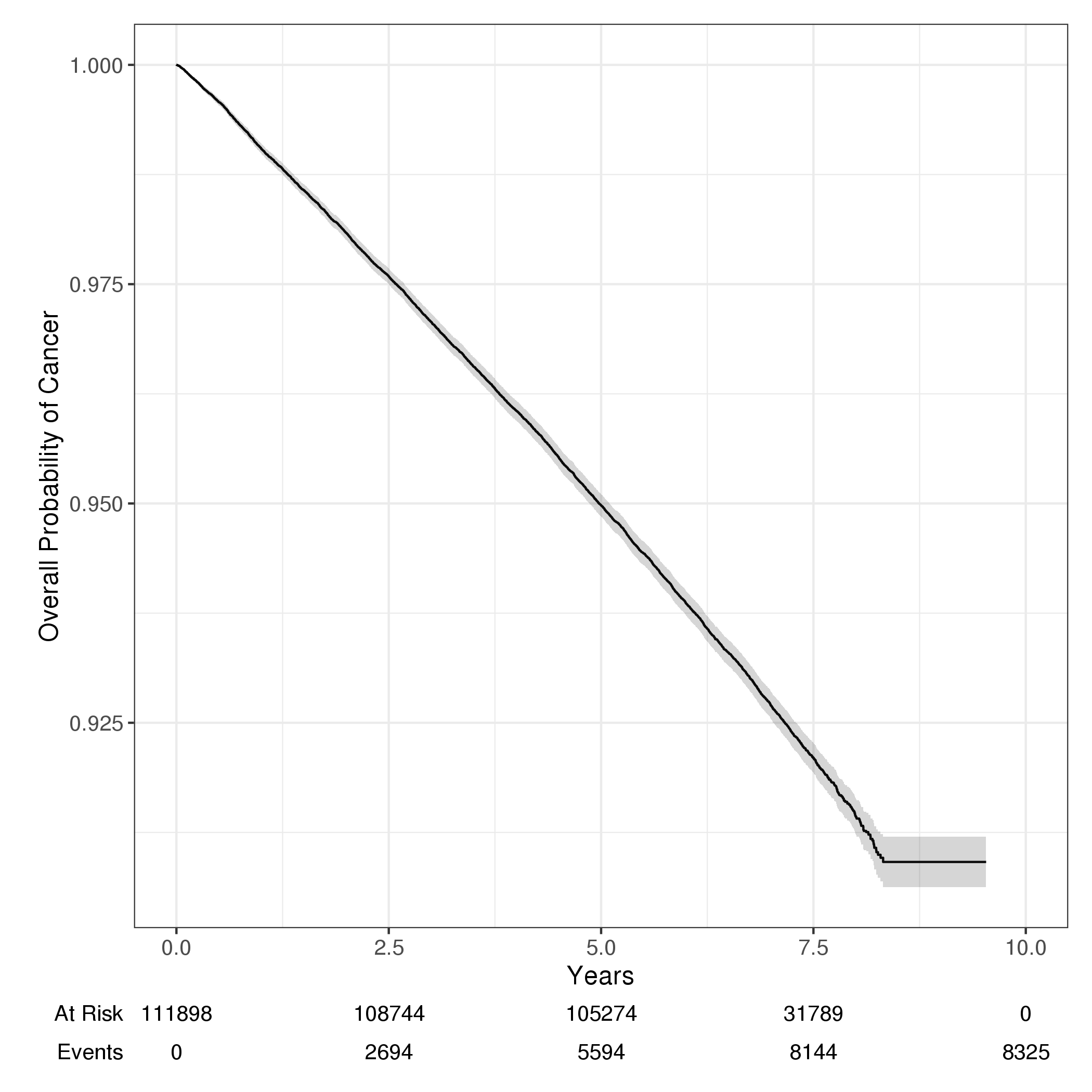


Figures S2 – subgroups (General CMS)


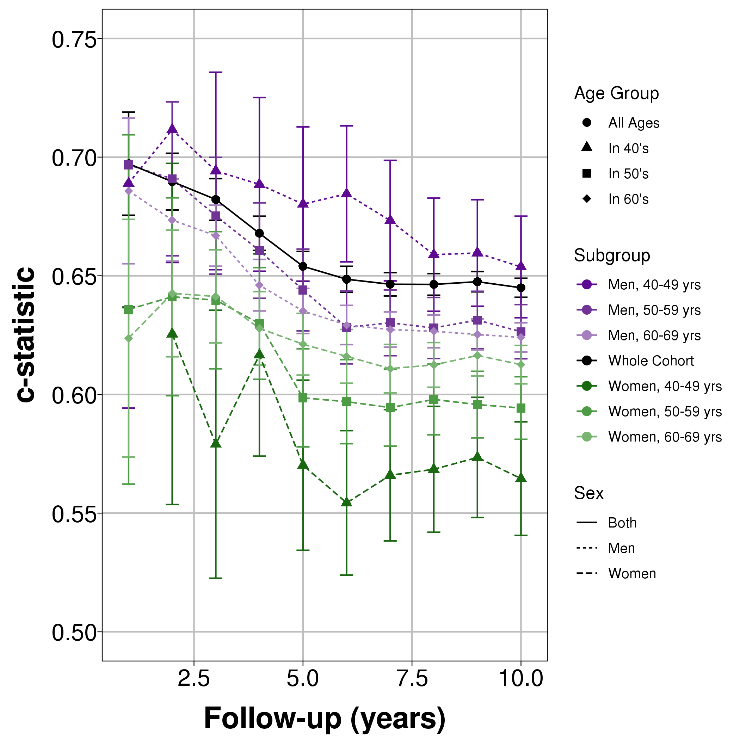

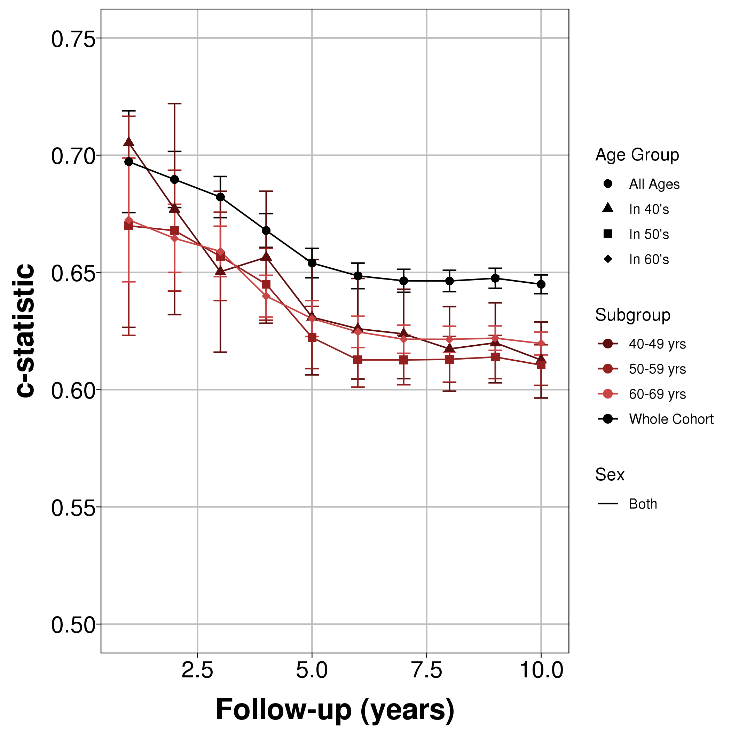

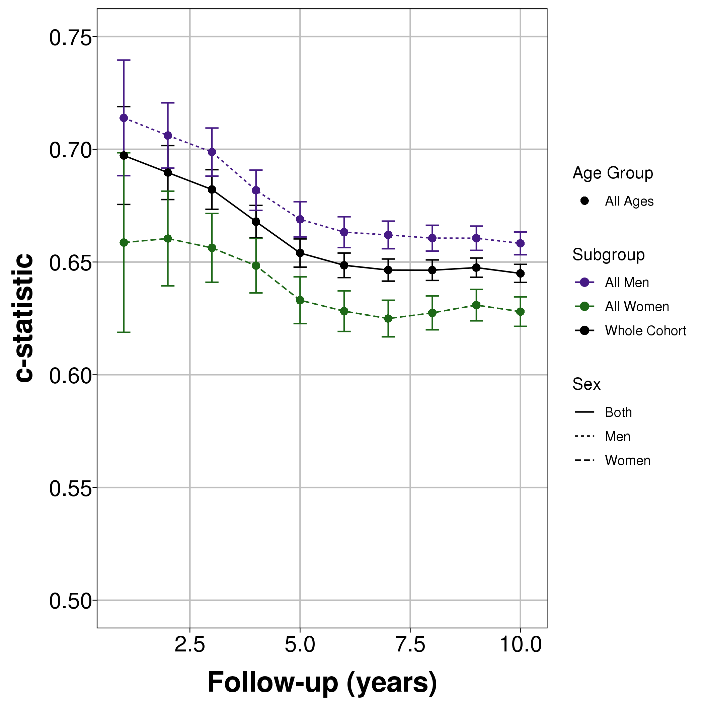
S2a-c: Discrimination of models over time for the outcome of death (by subgroups of sex, age, and sex & age)

S2d-f: Discrimination of models over time for the outcome of GP consultation rate (by subgroups of sex, age, and sex & age)


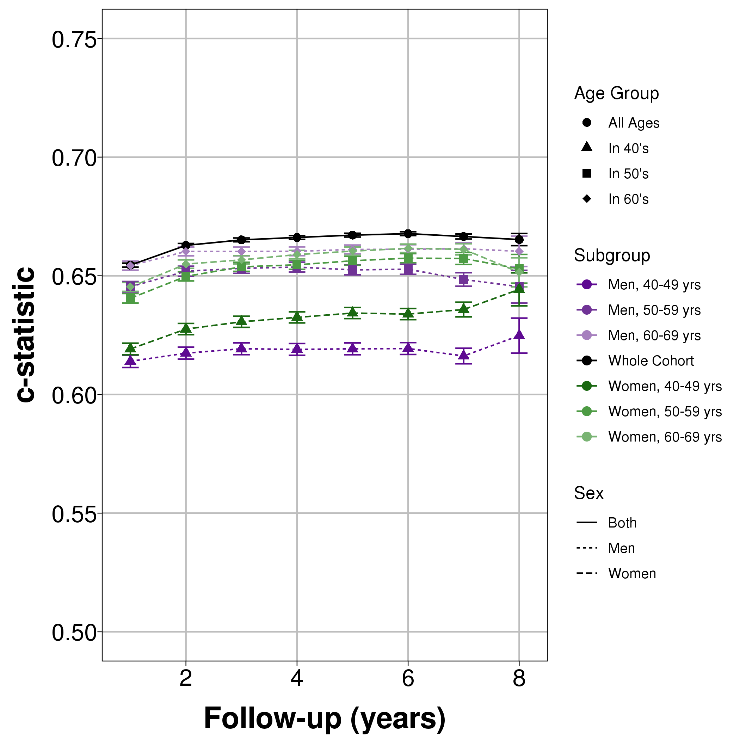

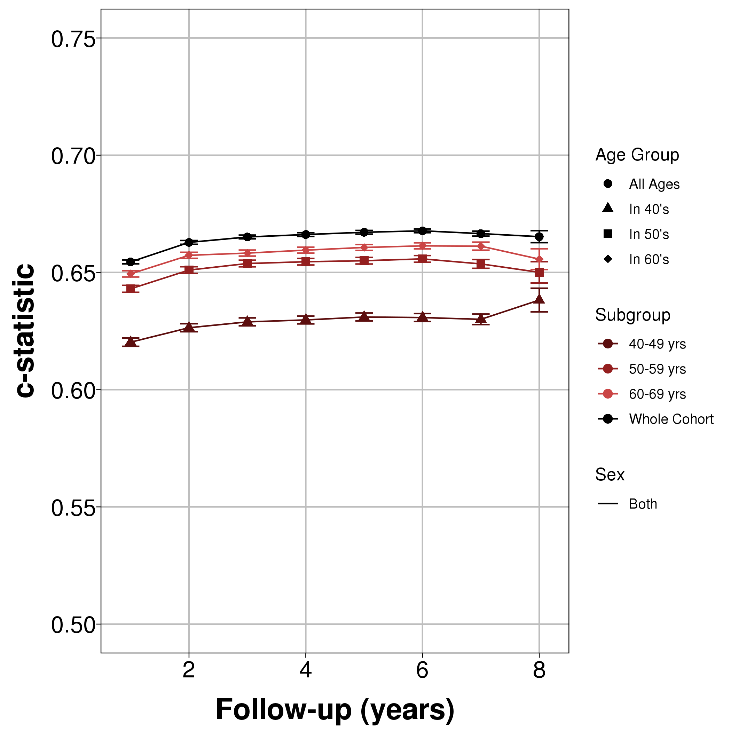

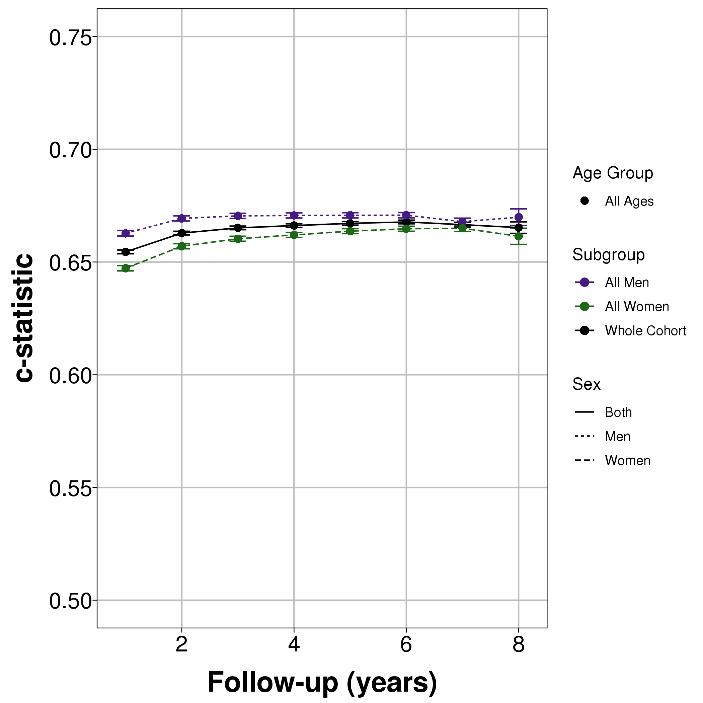


S2g-i: Discrimination of models over time for the outcome of cancer diagnosis (by subgroups of sex, age, and sex & age)


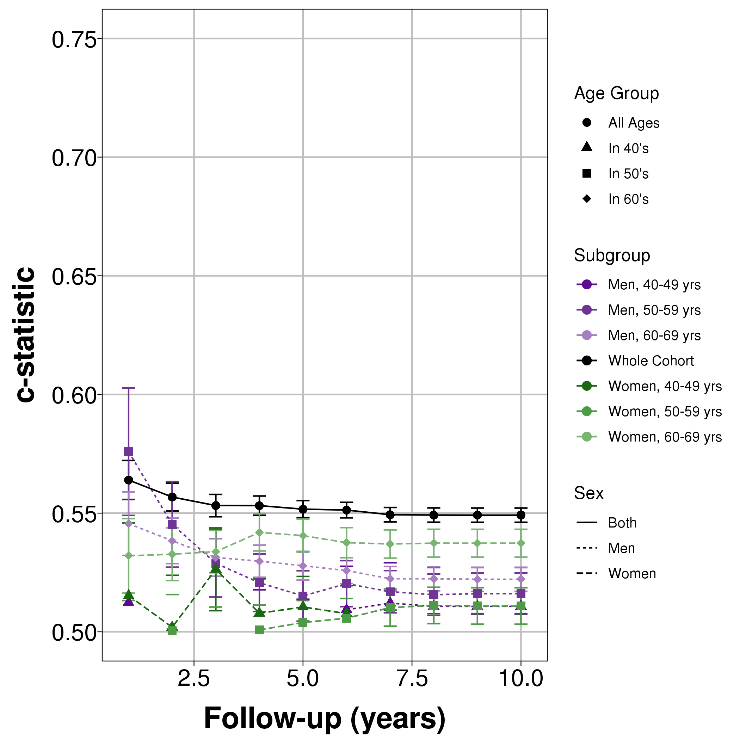

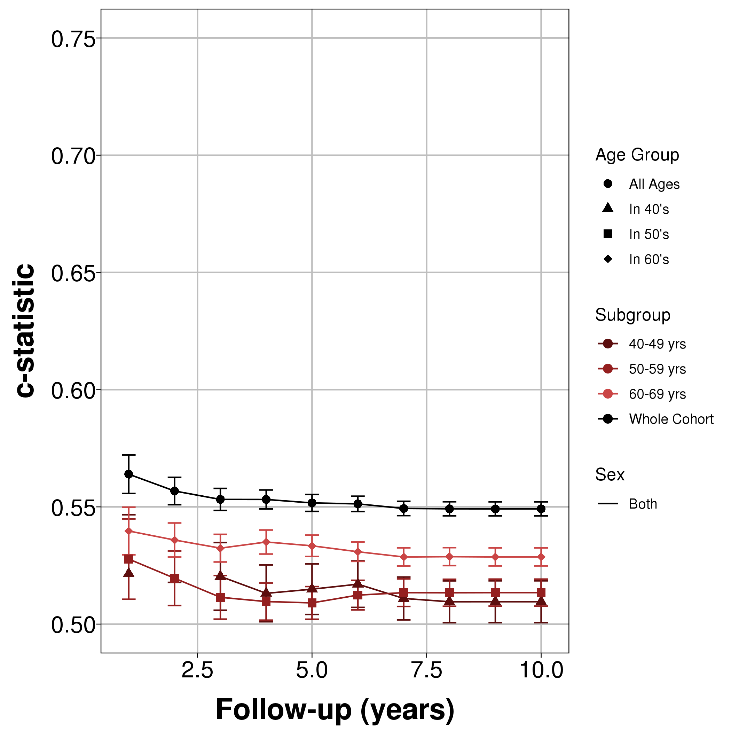

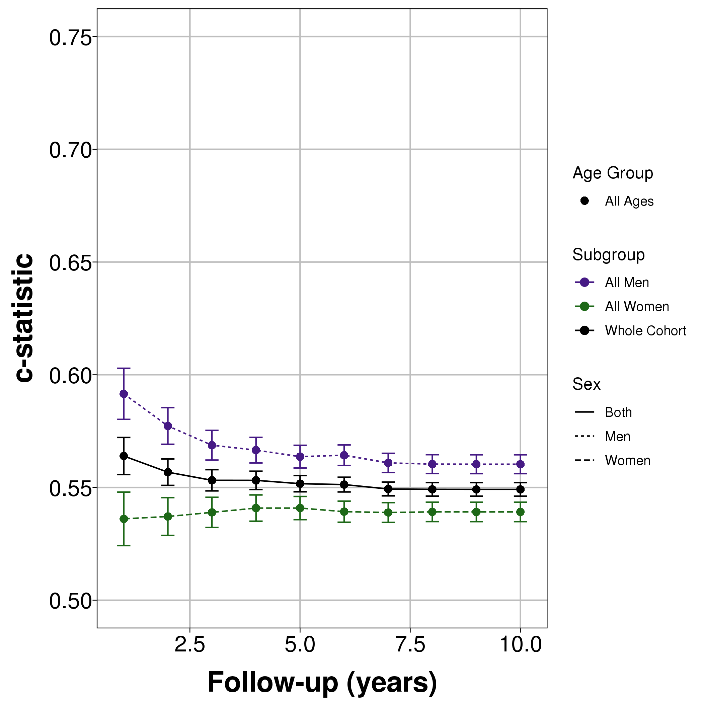


Figure S3 - Calibration plots

*Calibration plots for the (rows) general CMS, unweighted count score (short and long versions), hospital CMS (short and long versions) and the mortality CMS (short and long version) for the (columns) three outcomes included in the analysis (mortality, GP consultation rate and cancer diagnosis). The calibration was assessed at two time points (1 and 5 years) which are given on sequential rows for each of the model versions. The plots present the risk computed by the CMS on the x-axis (predicted relative risk) by decile, and the observed risk (i.e. proportion of people with the outcome) is plotted for each decile on the y-axis (observed relative risk in the UKB cohort). The predicted and observed risk for each decile are normalised relative to the values of for the 5^th^ decile.*

|  | **Outcome: Mortality** | **Outcome: GP Consultation Rate** | **Outcome: Cancer Diagnosis** |
| --- | --- | --- | --- |
| General CMS (1 year follow-up) | 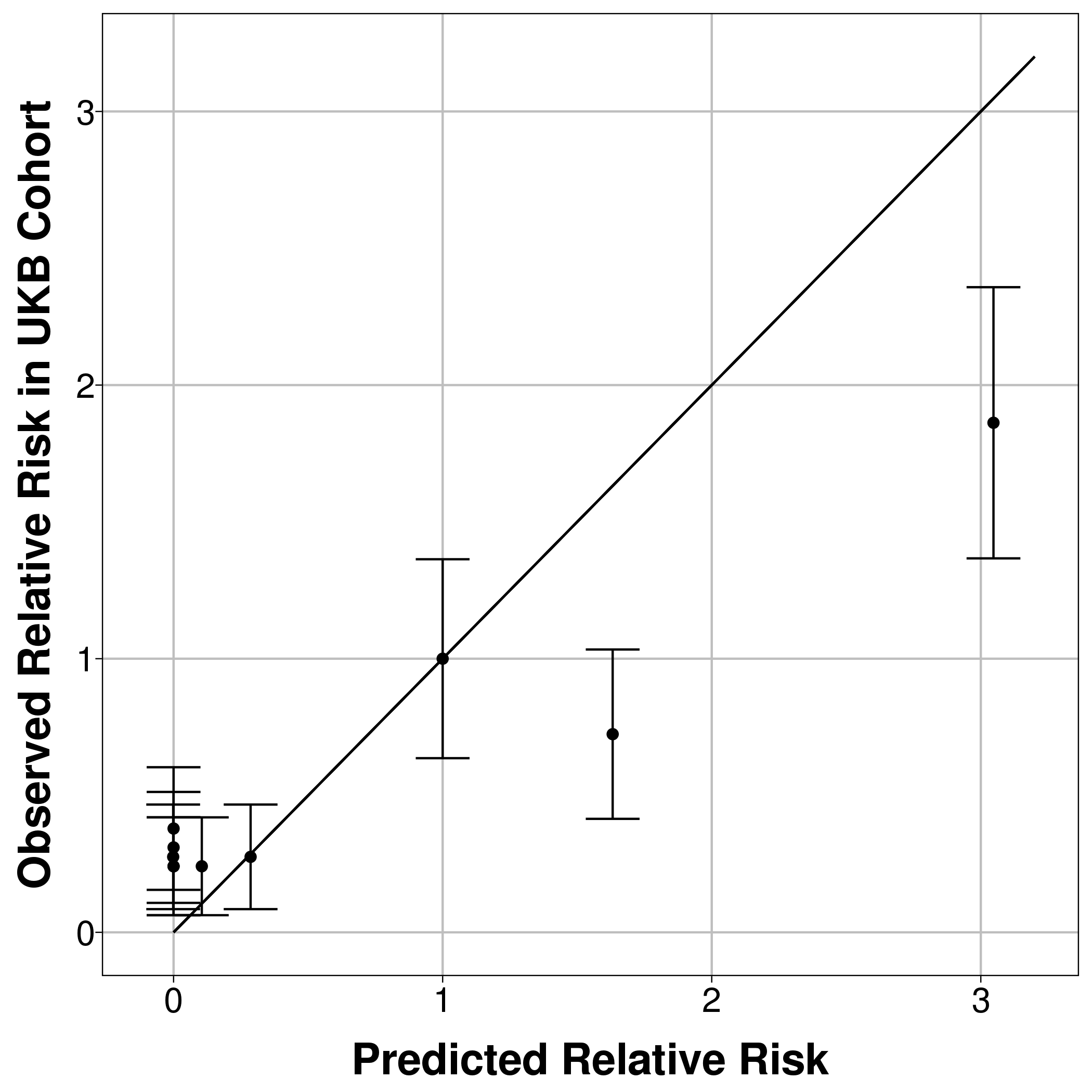 | 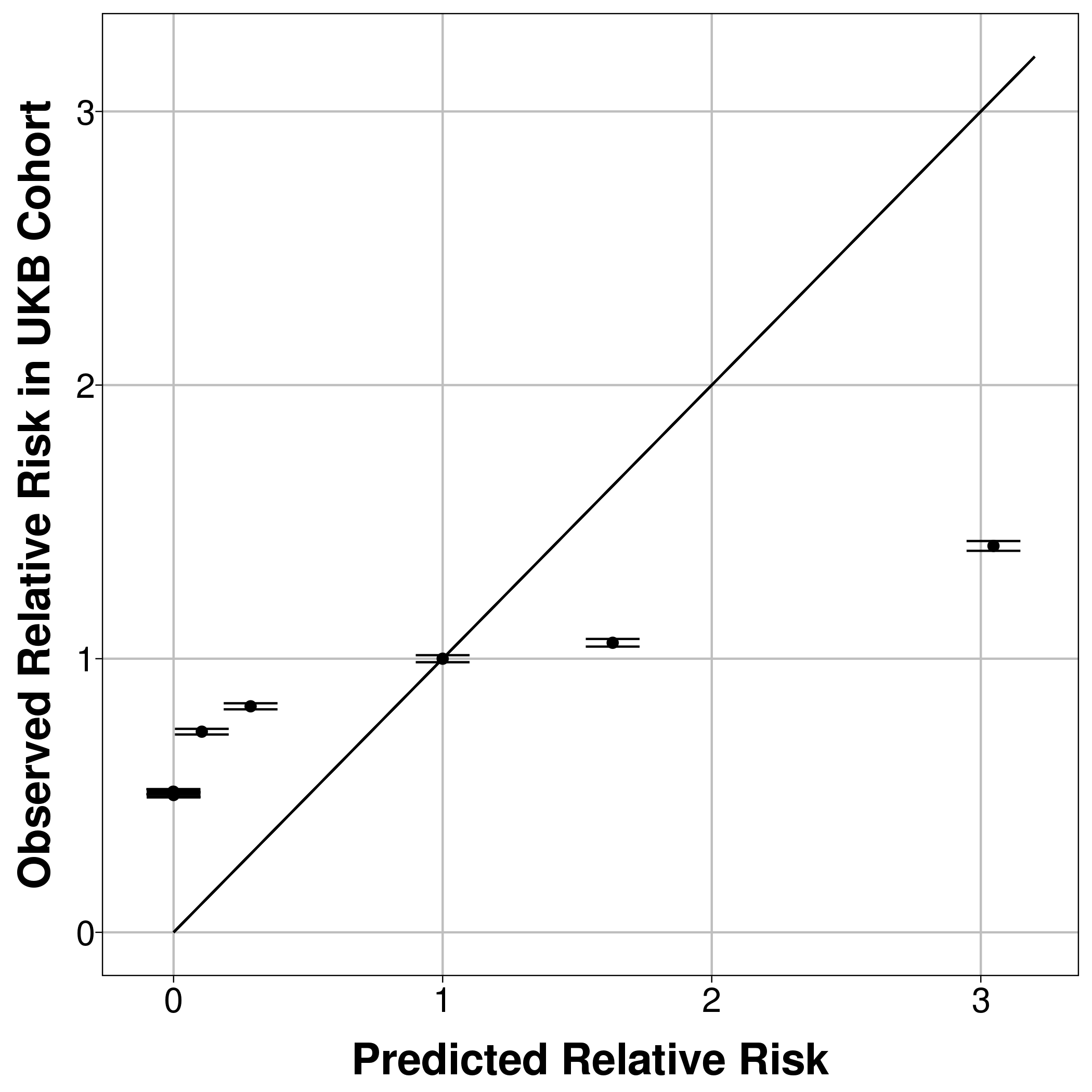 | 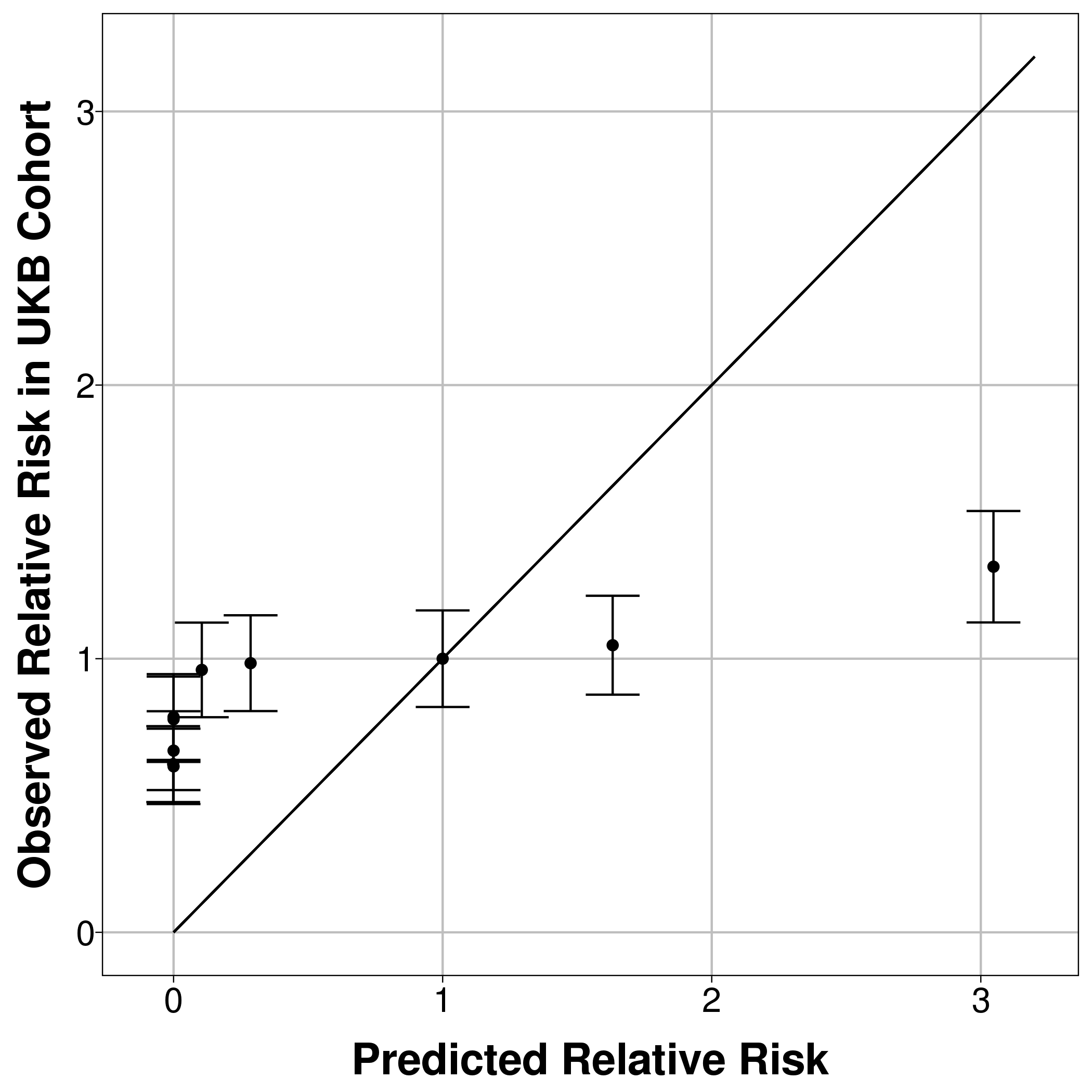 |
| General CMS (5 years follow-up) | 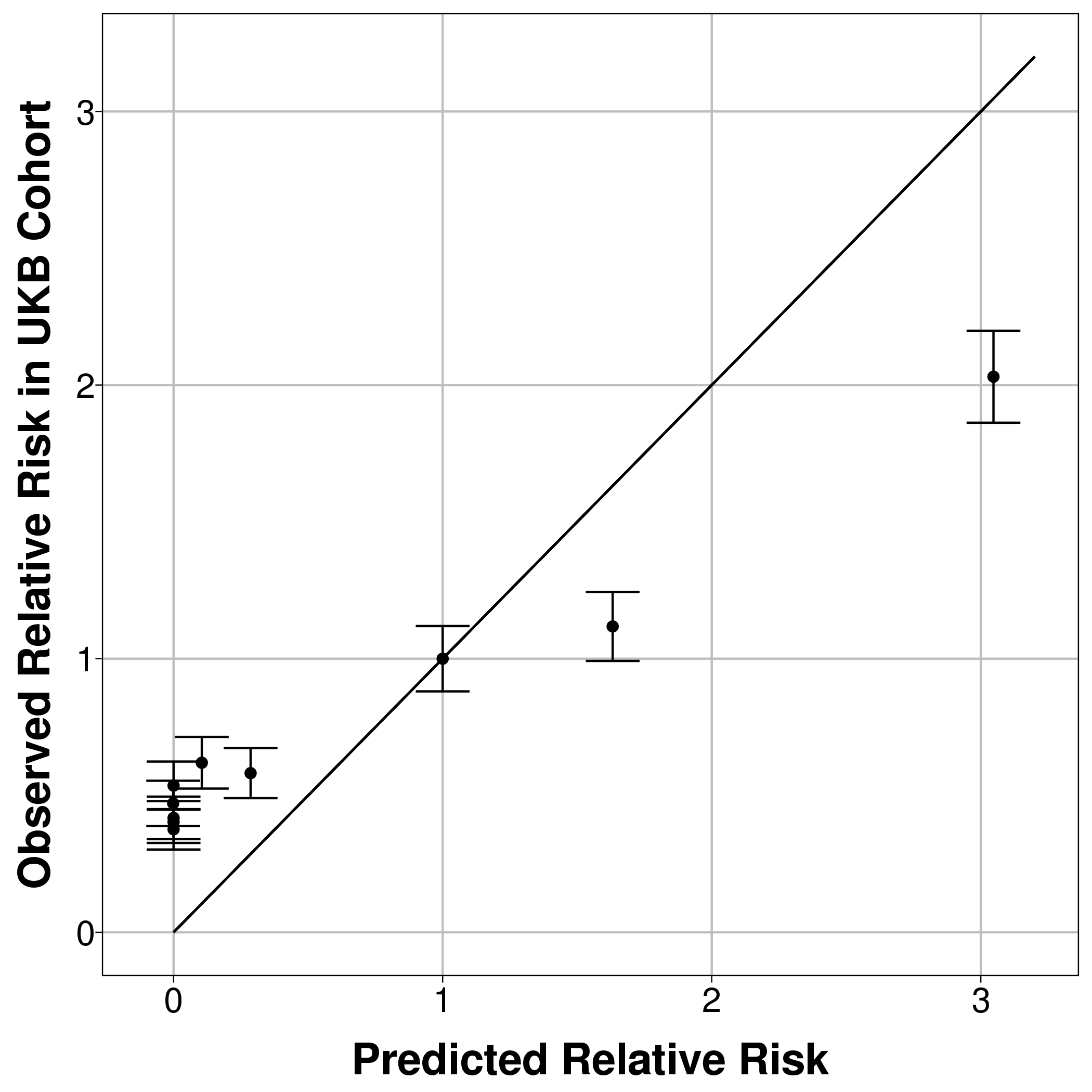 | 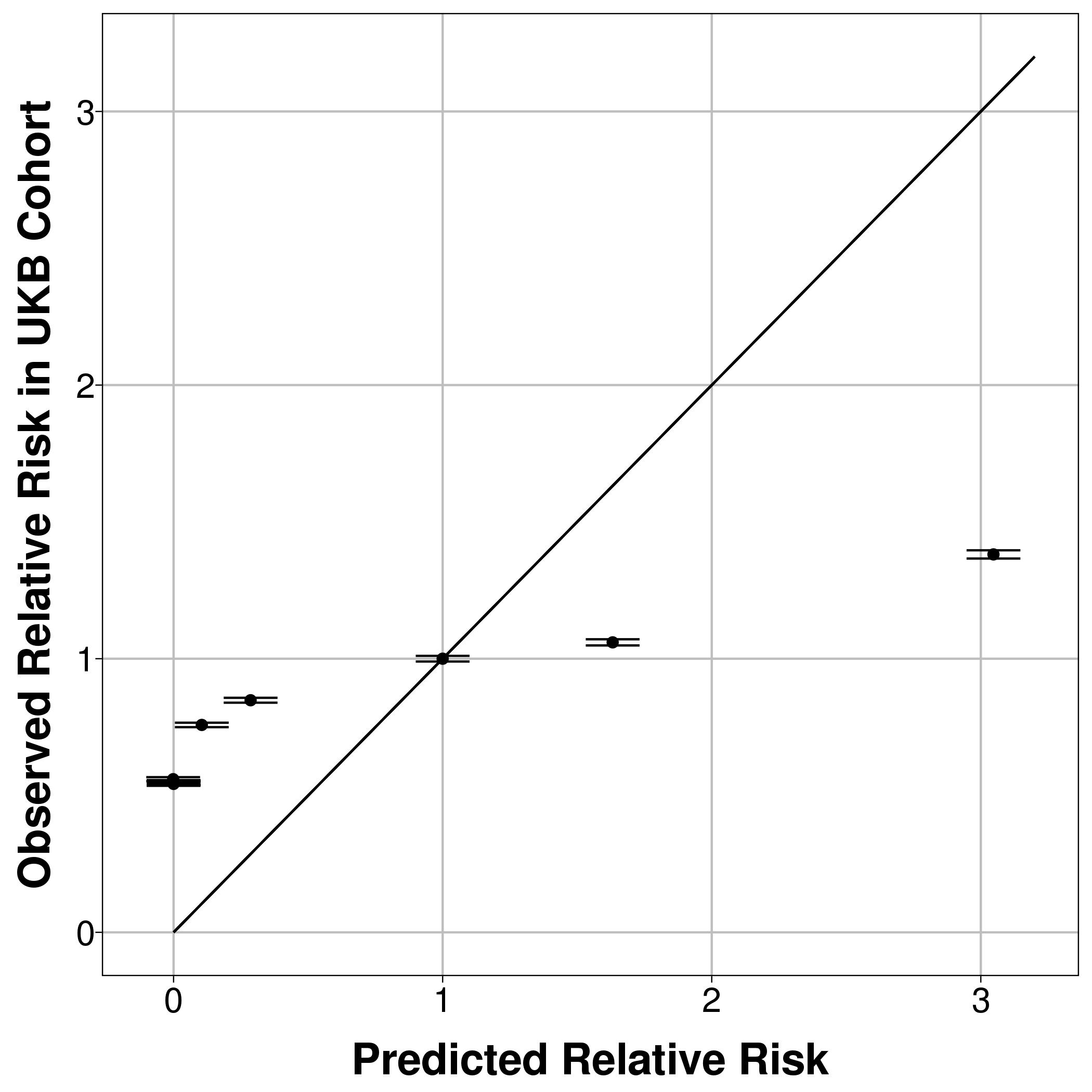 | 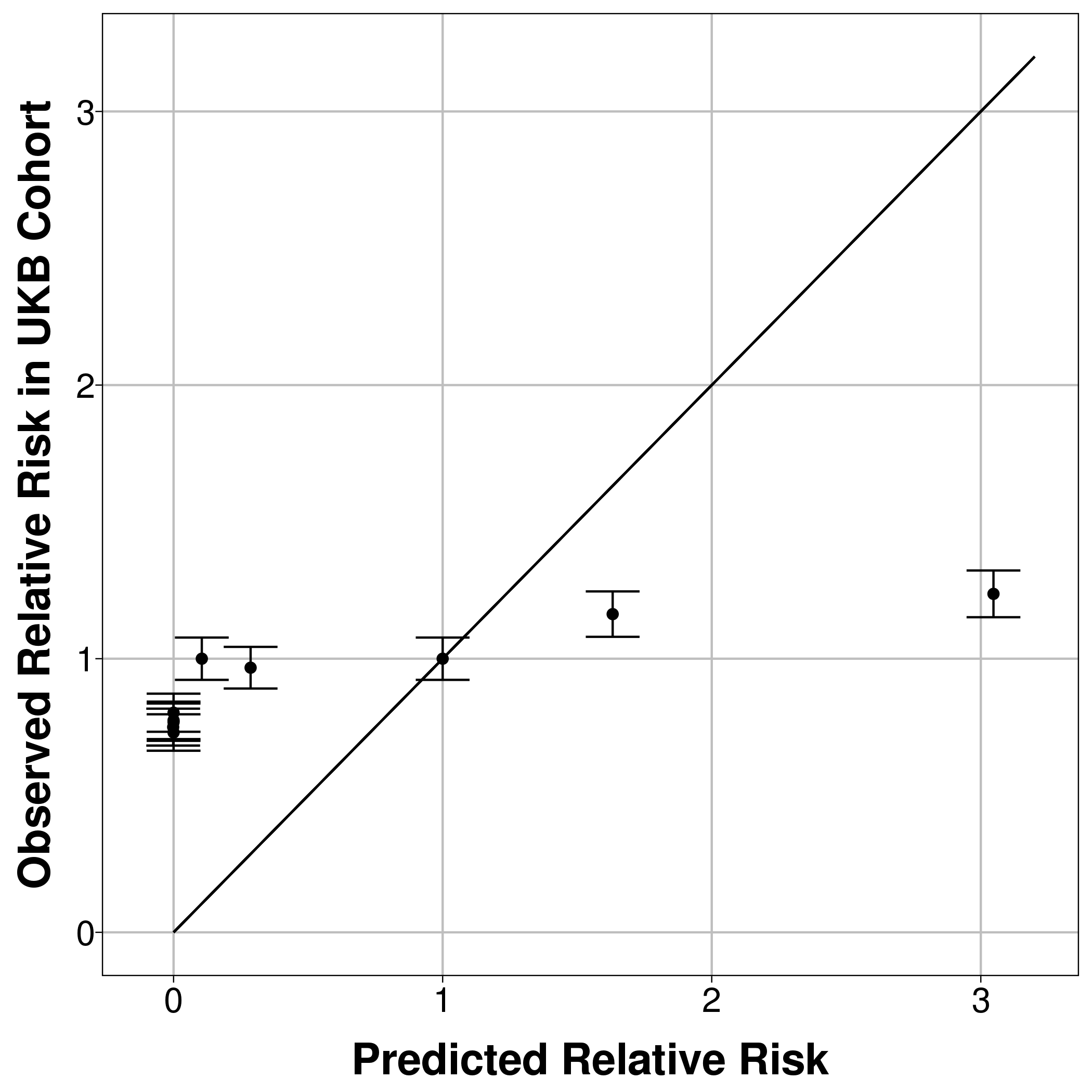 |
| Unweighted count score (1 year follow-up) | 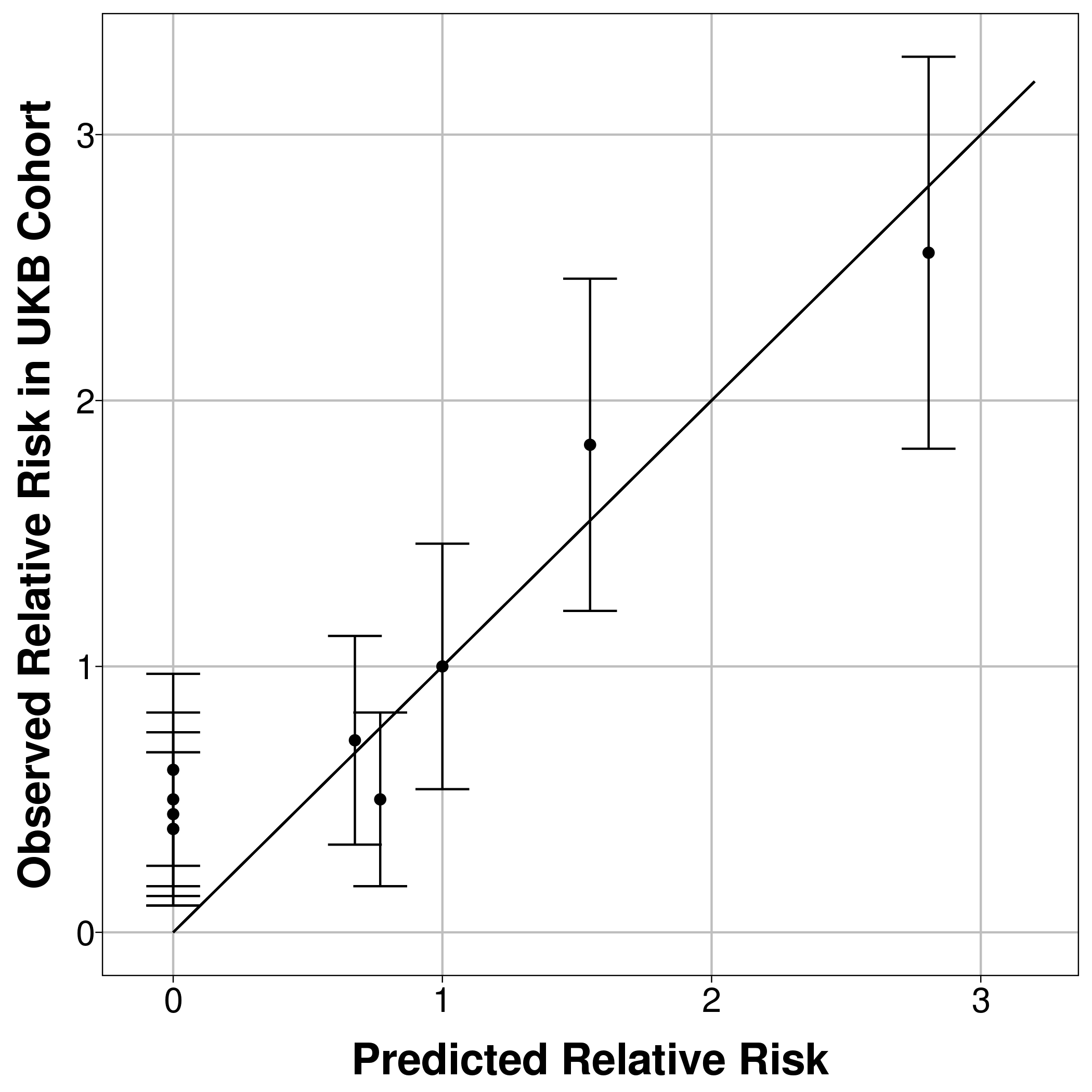 | 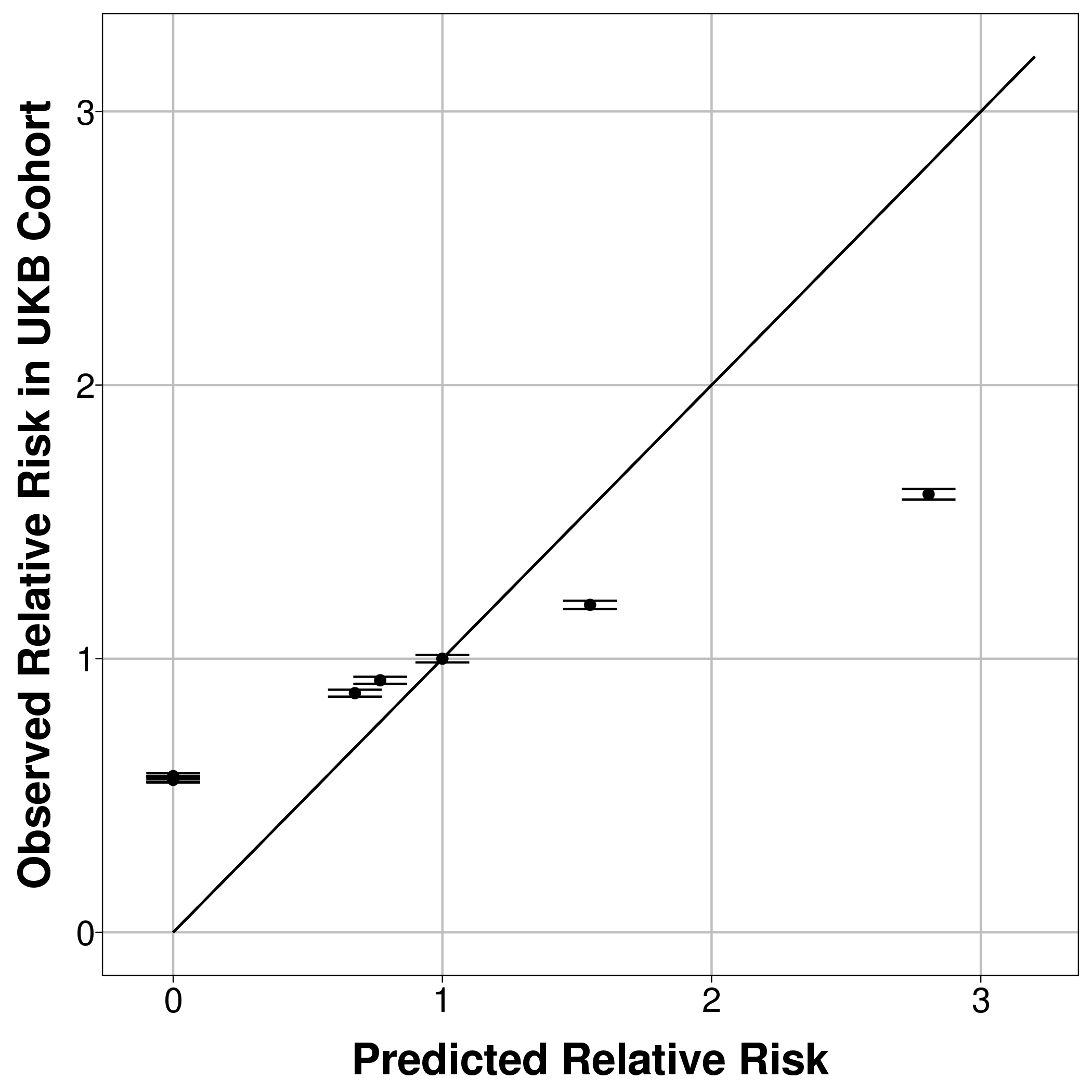 | 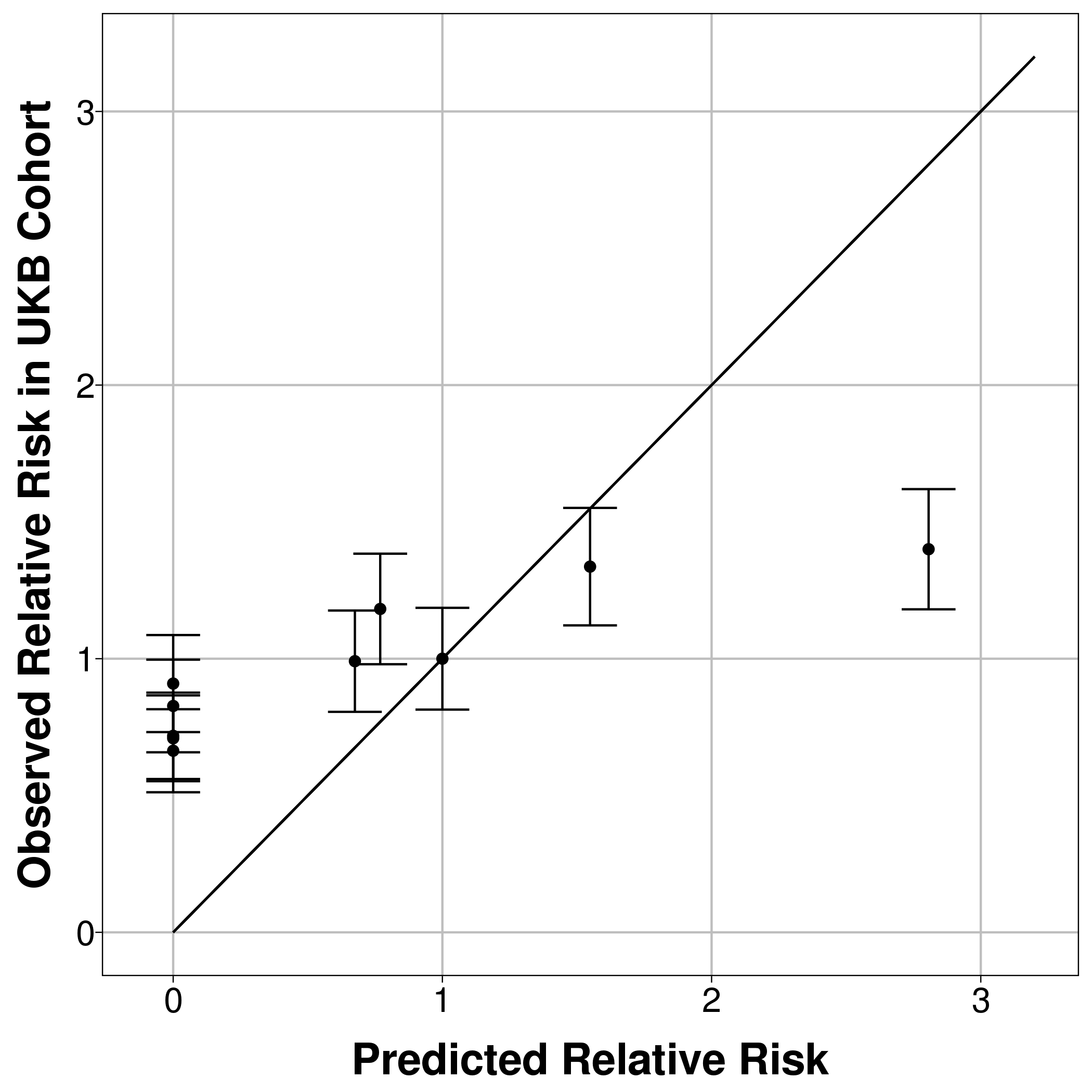 |
| Unweighted count score (5 years follow-up) | 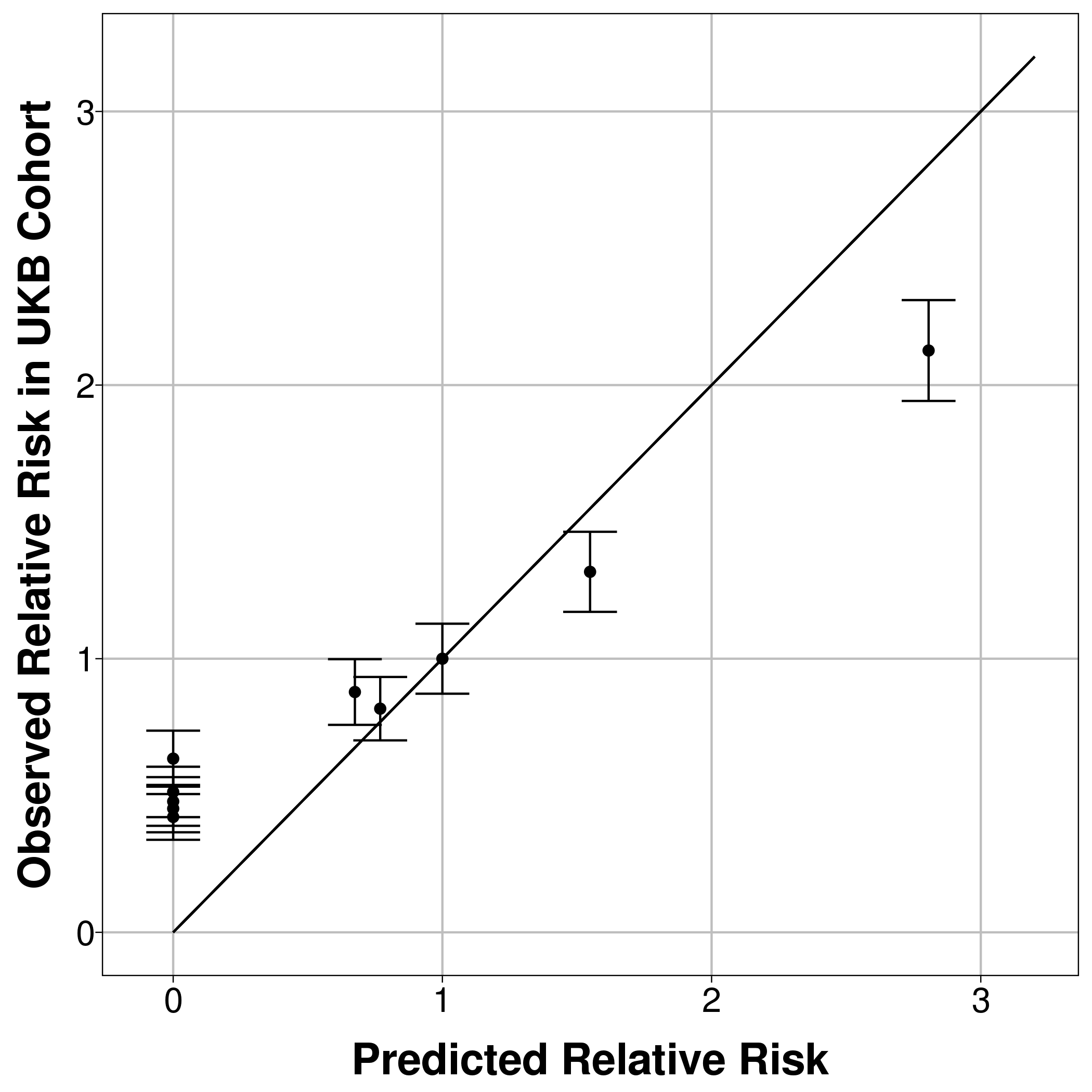 | 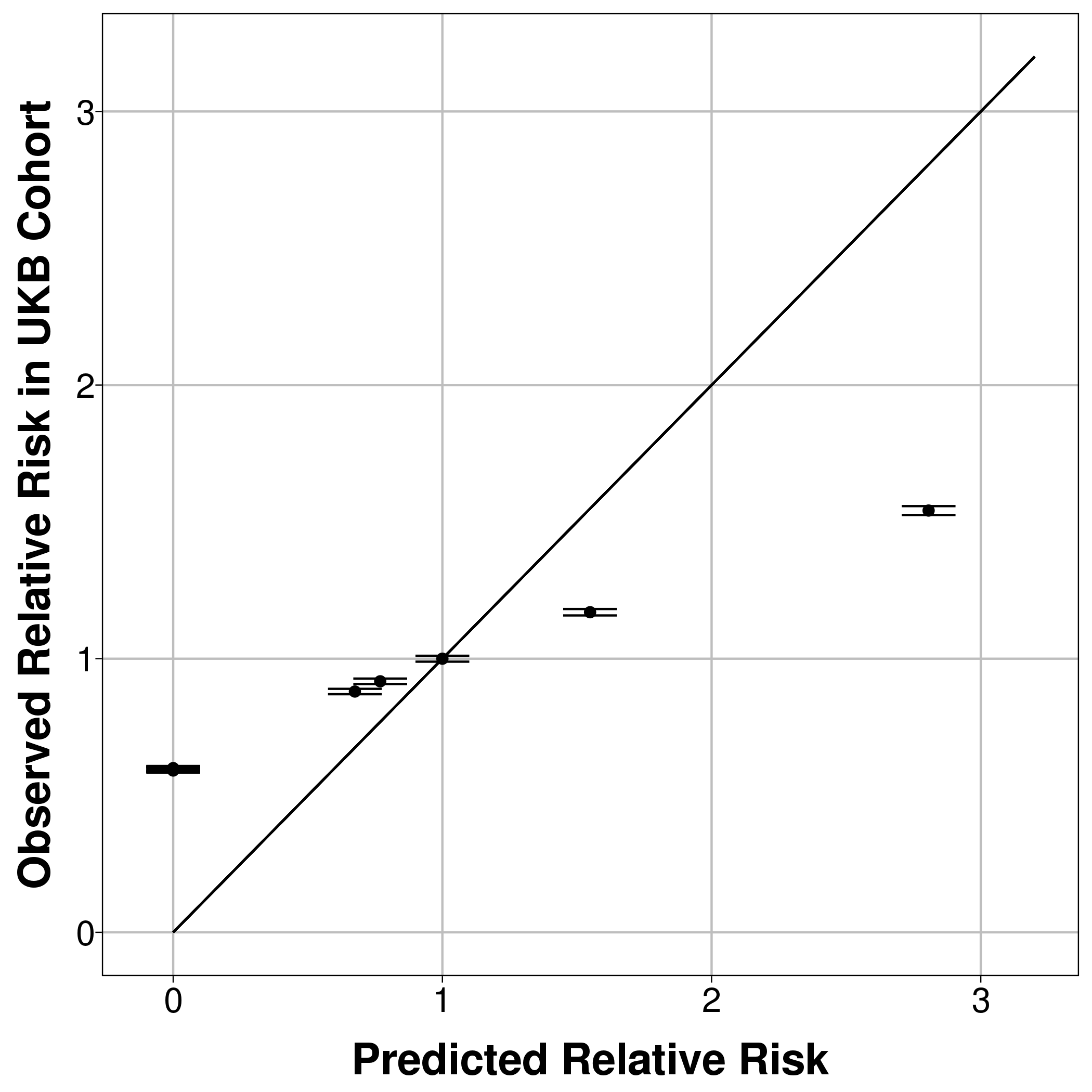 | 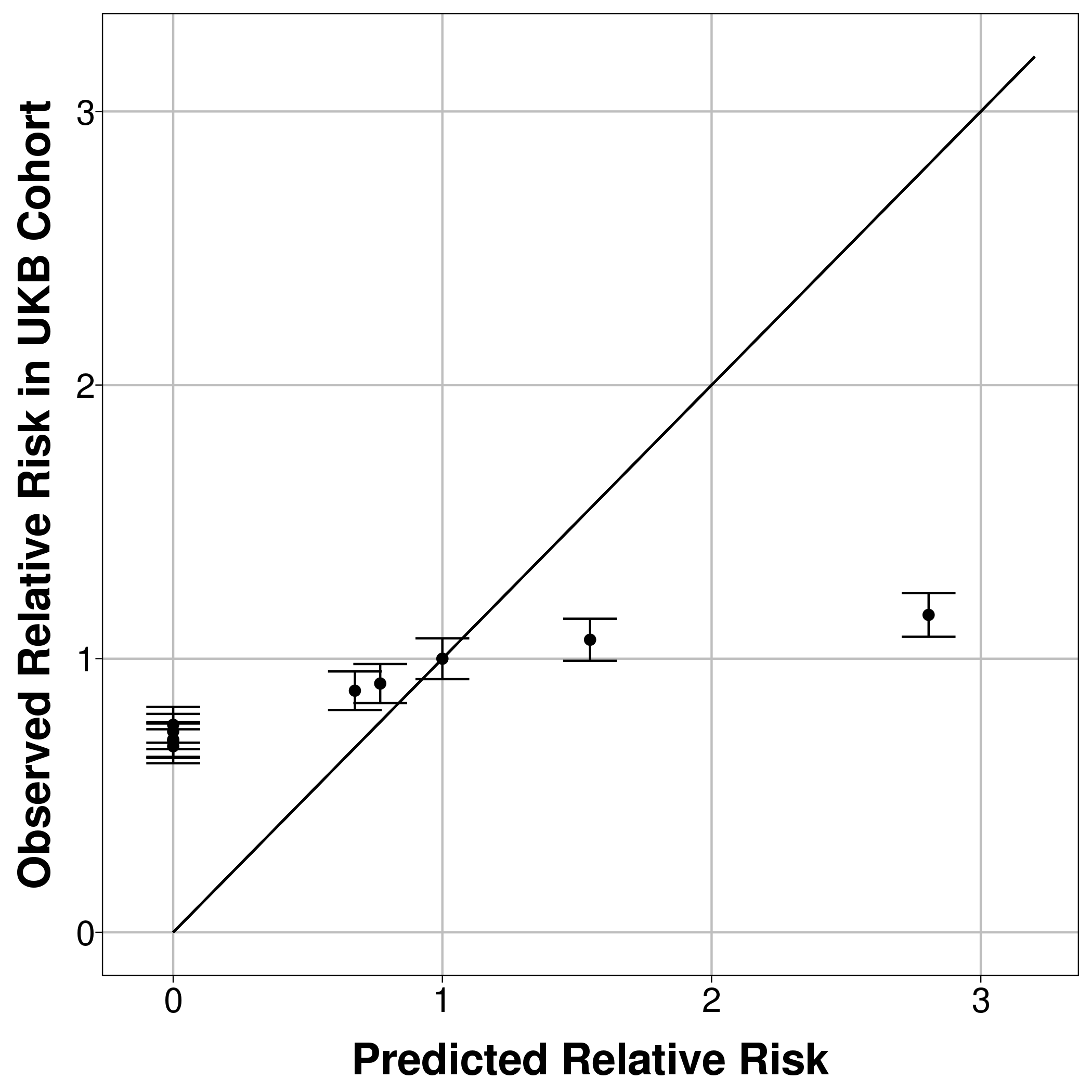 |
| Unweighted count score – short version with only 20 conditions (1 year follow-up) | 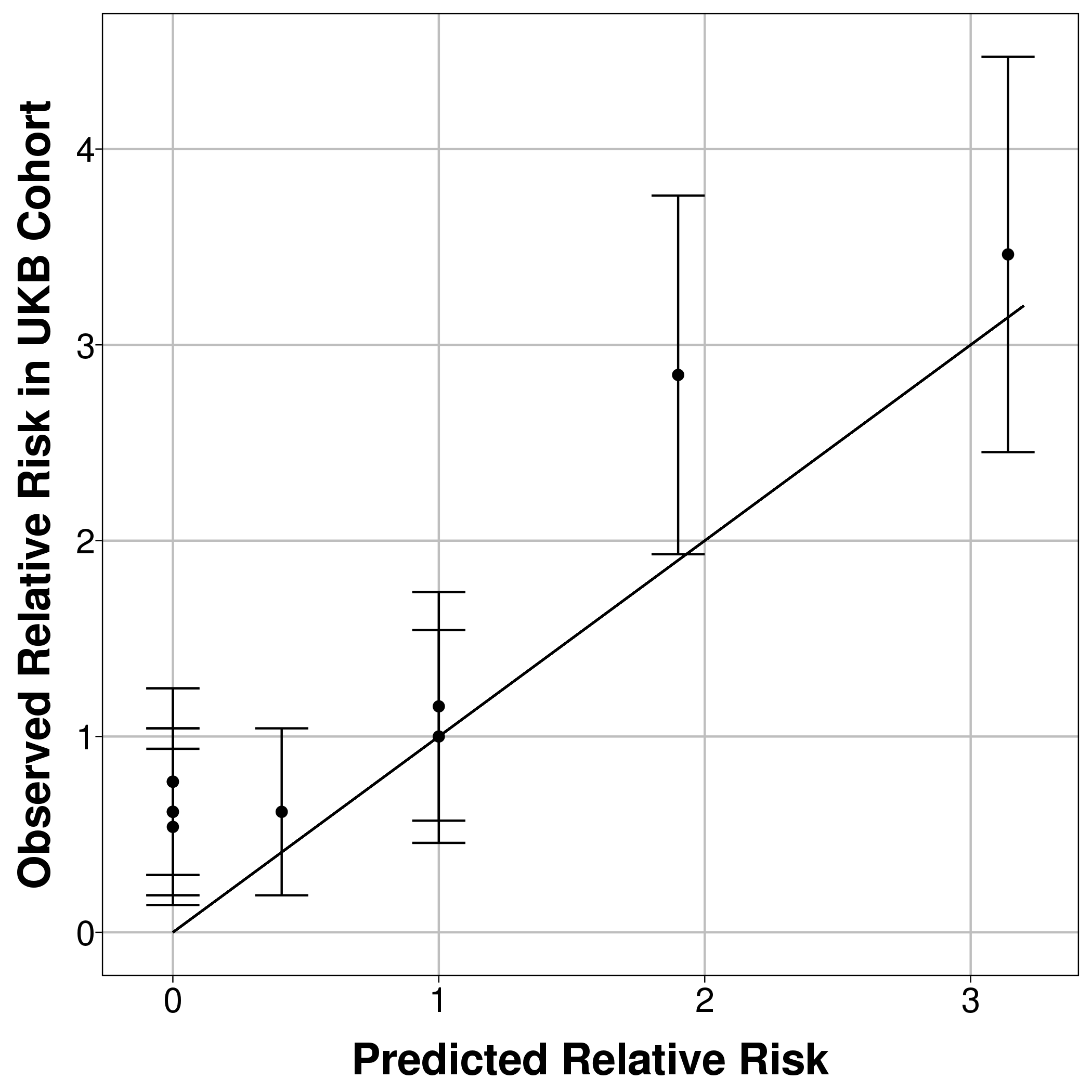 | 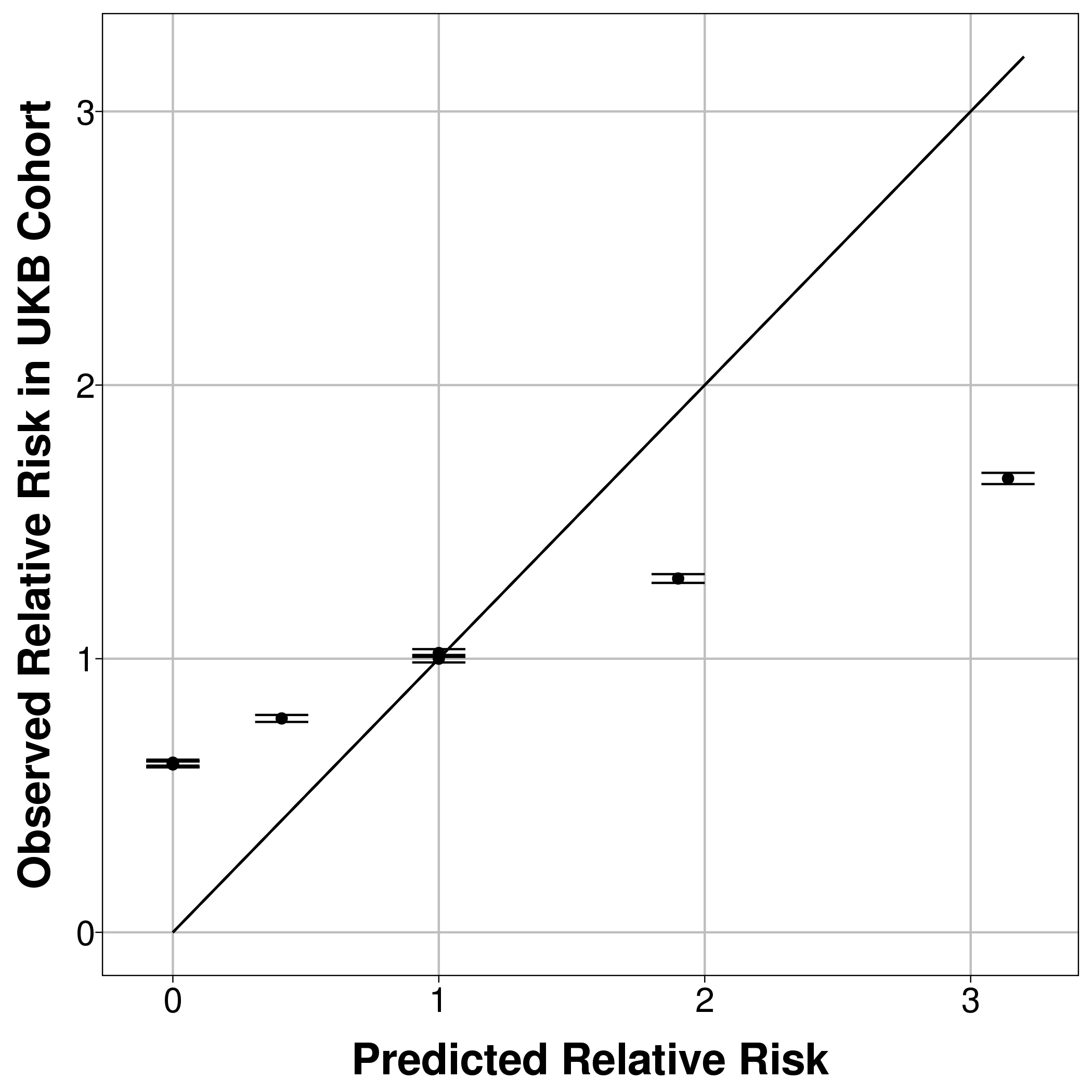 | 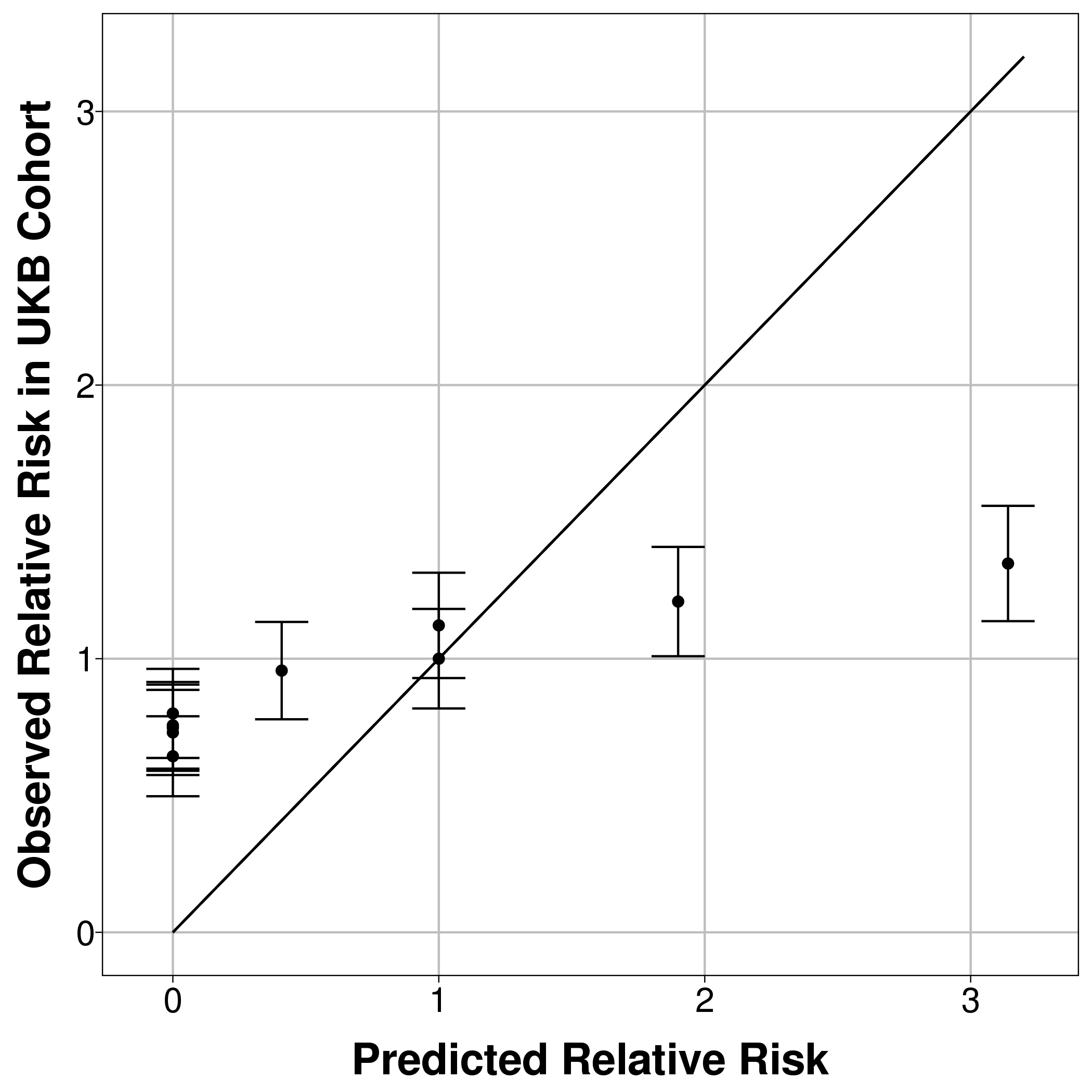 |
| Unweighted count score – short version with only 20 conditions (5 years follow-up) | 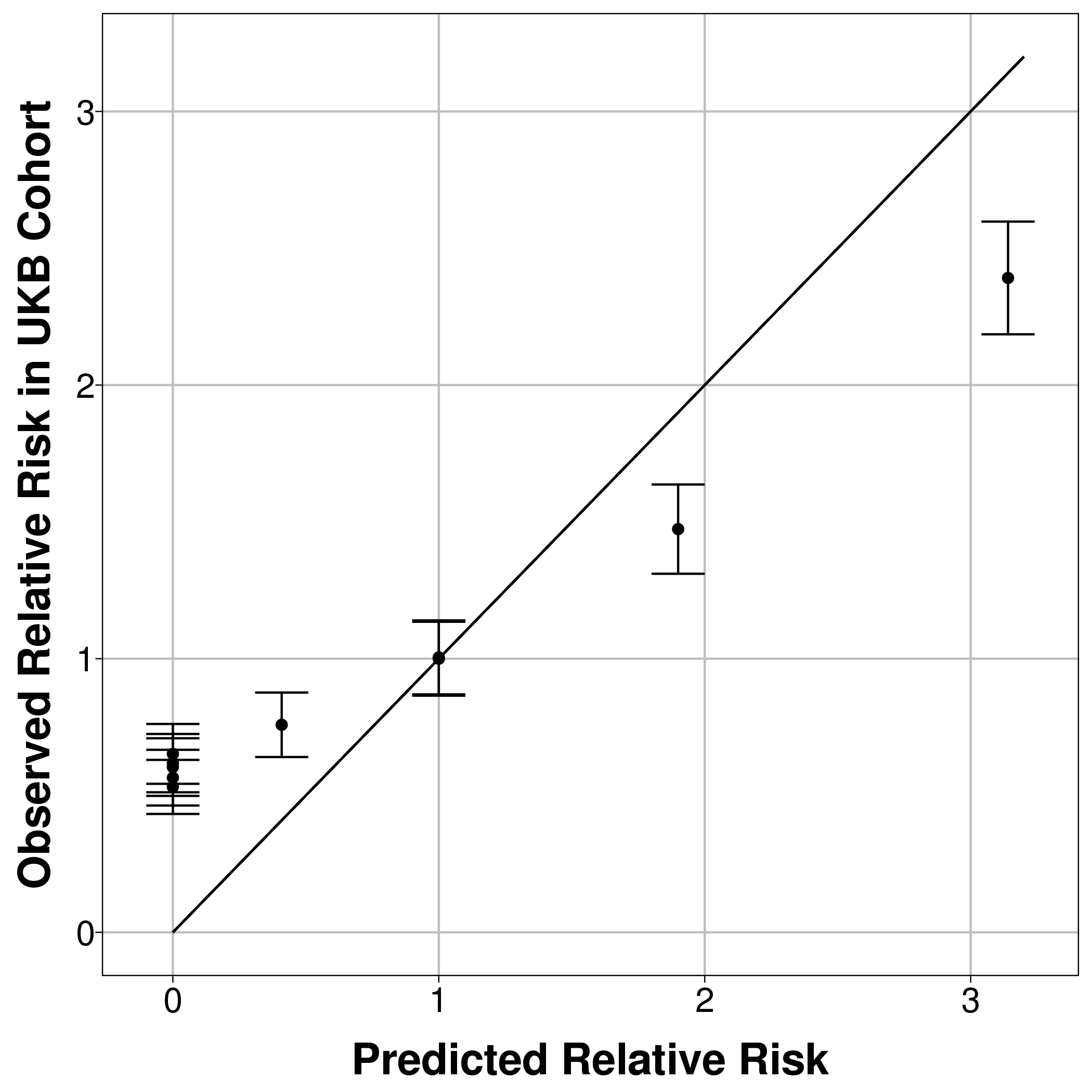 | 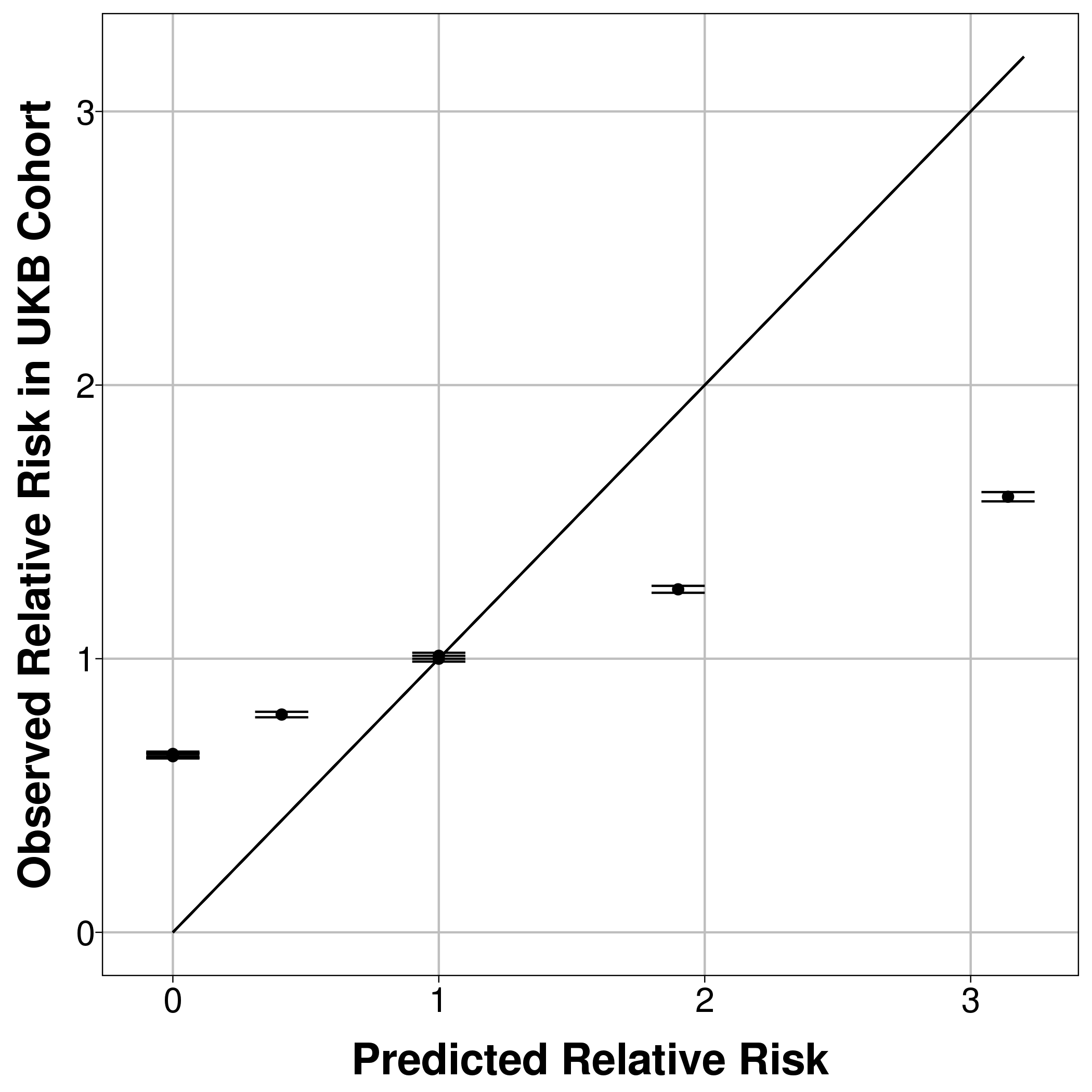 | 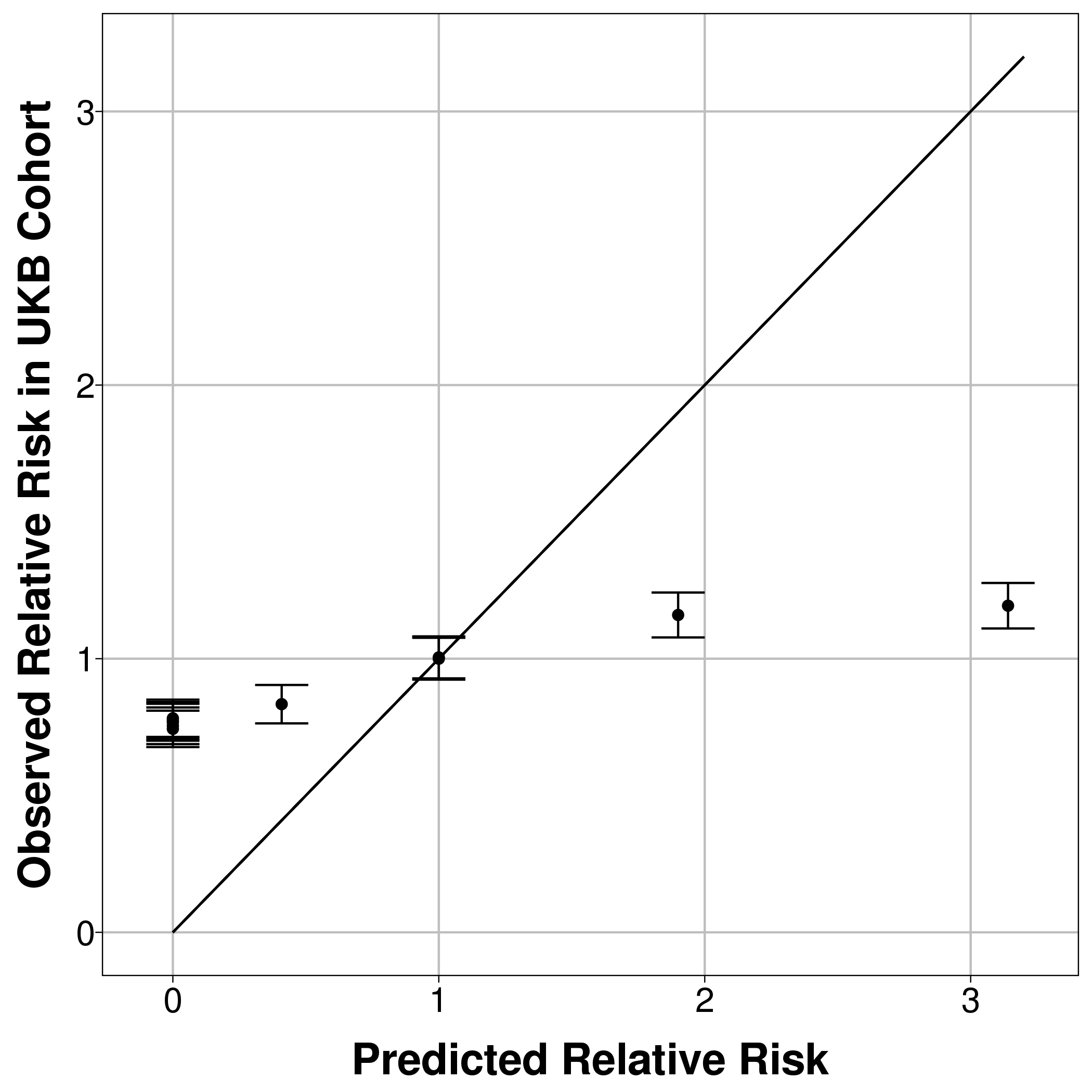 |
| Mortality CMS (1 year follow-up) | 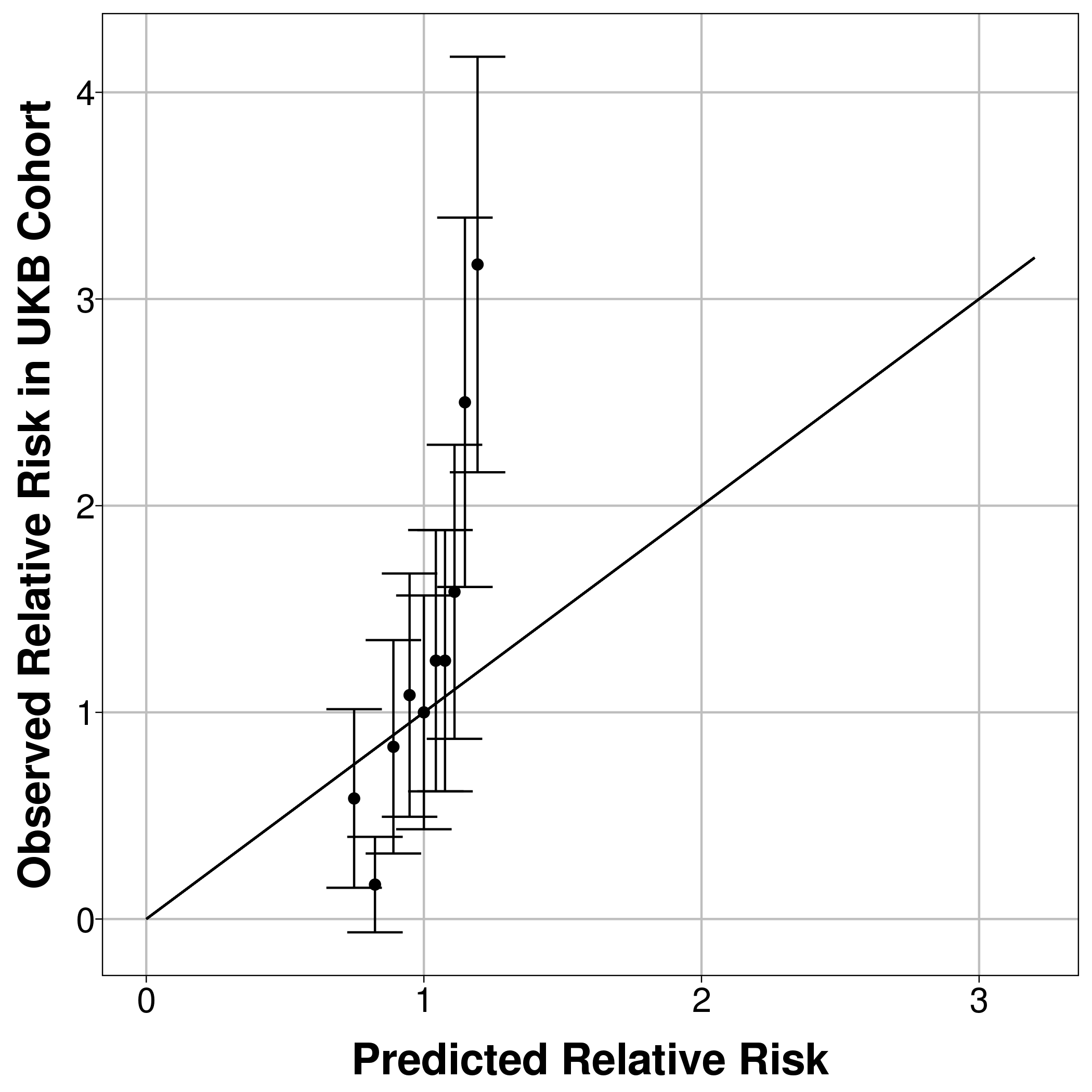 | 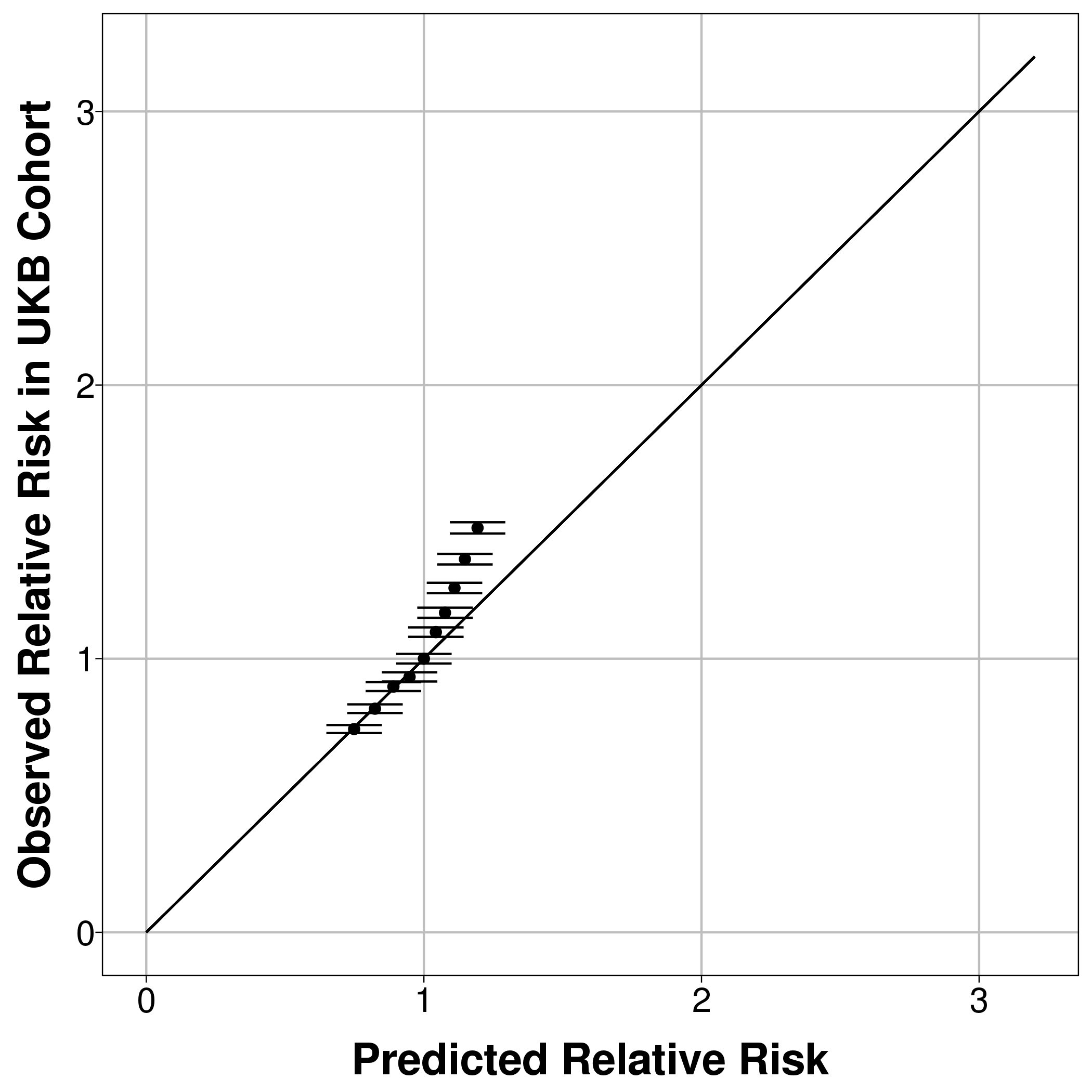 | 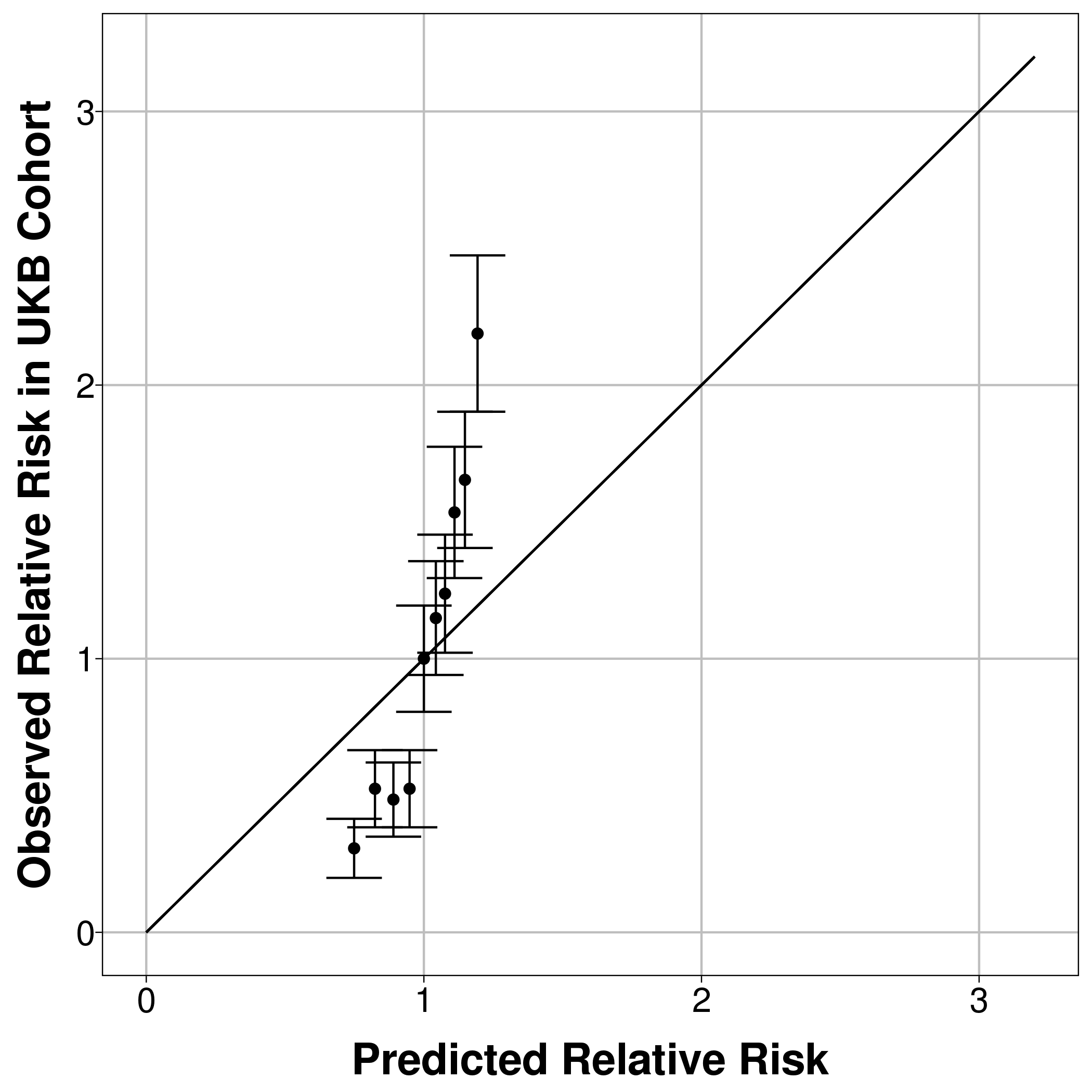 |
| Mortality CMS (5 years follow-up) | 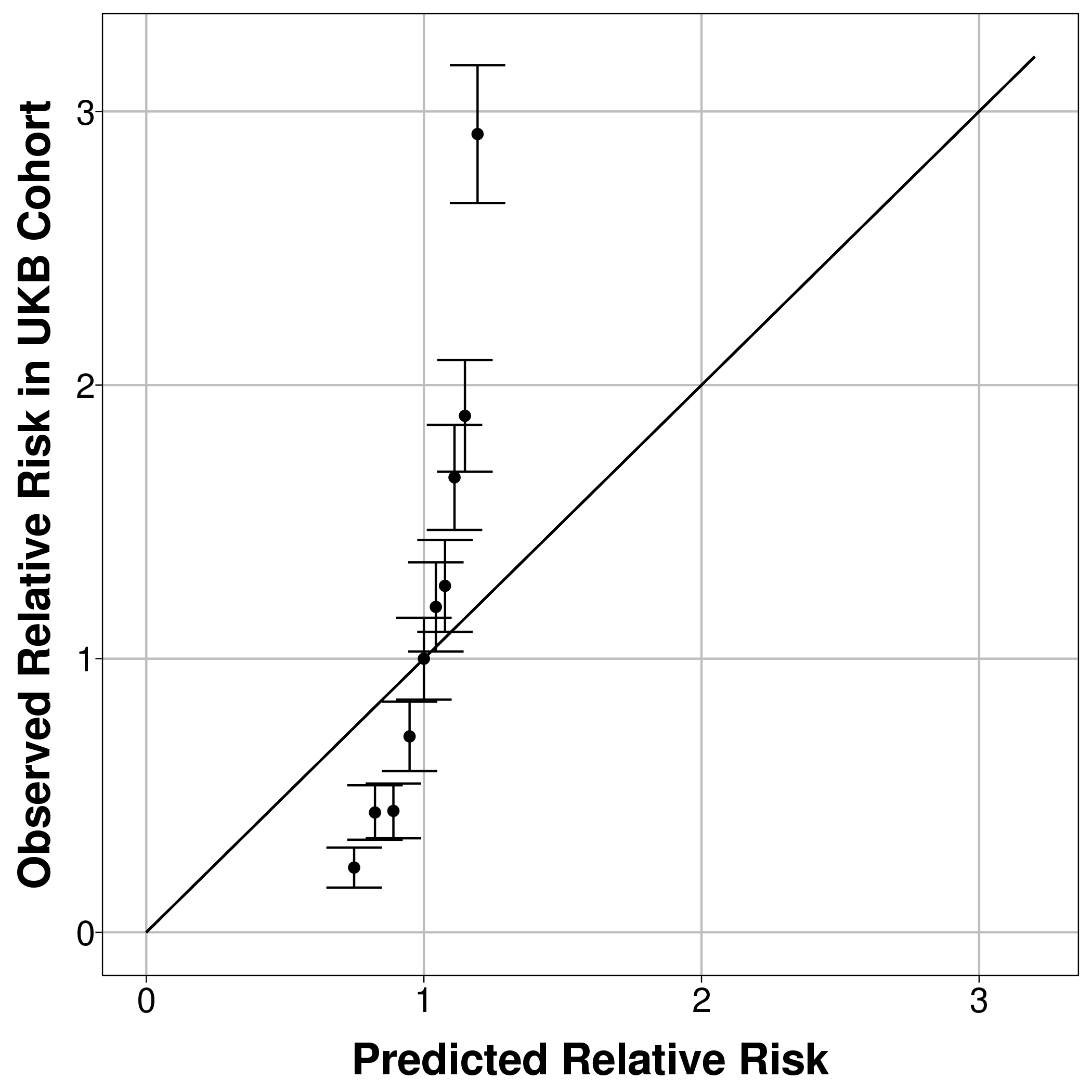 | 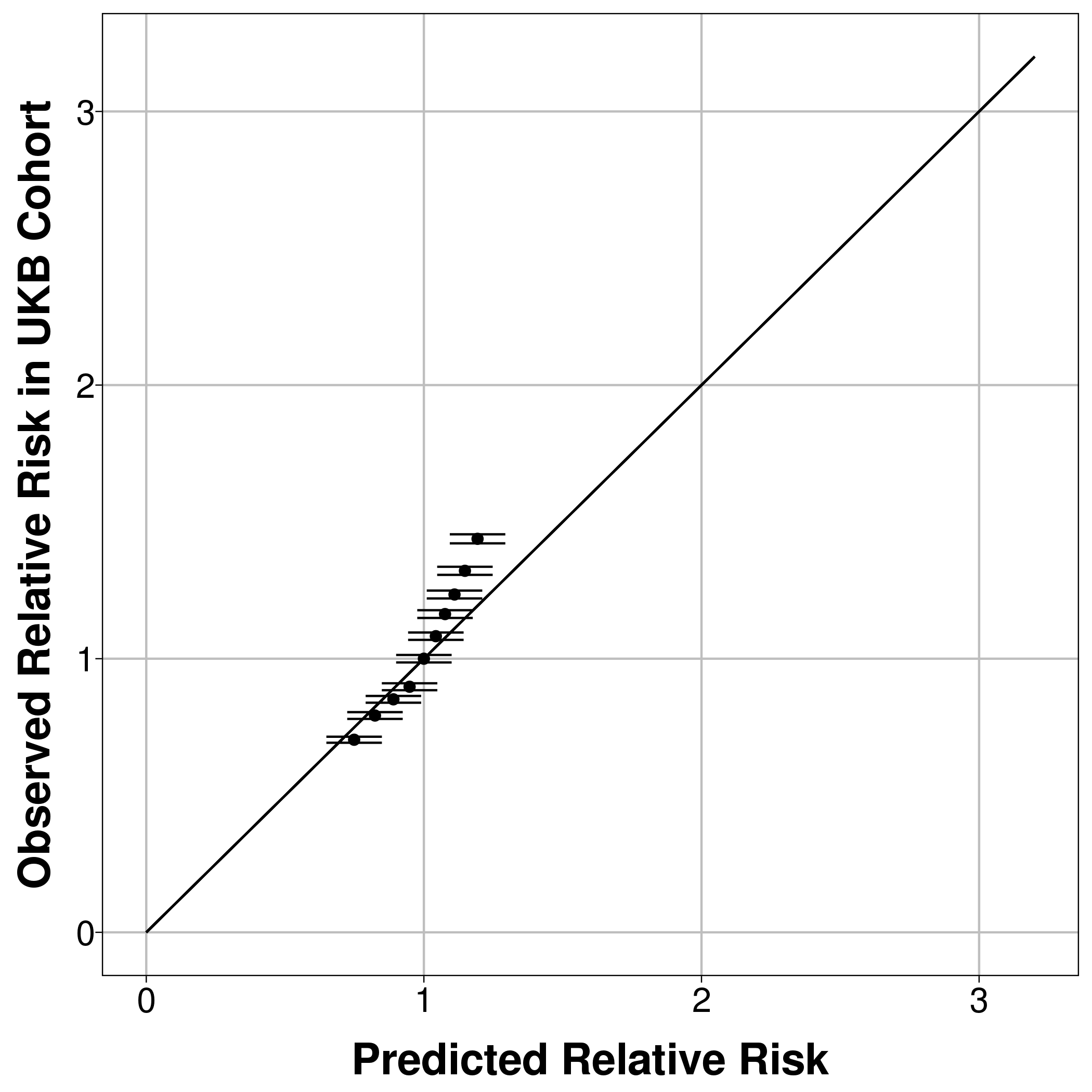 | 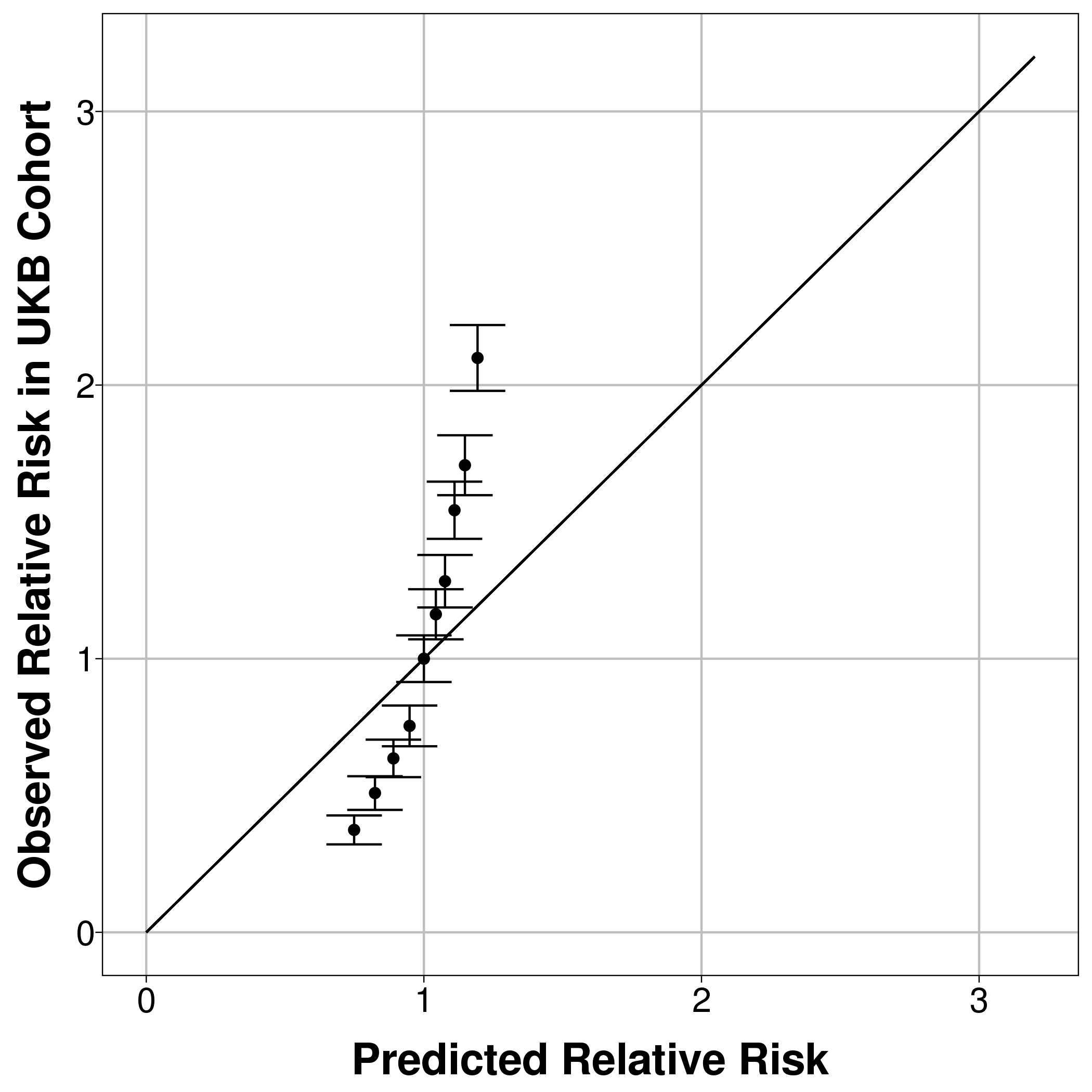 |
| Mortality CMS – short version with only 20 conditions (1 year follow-up) | 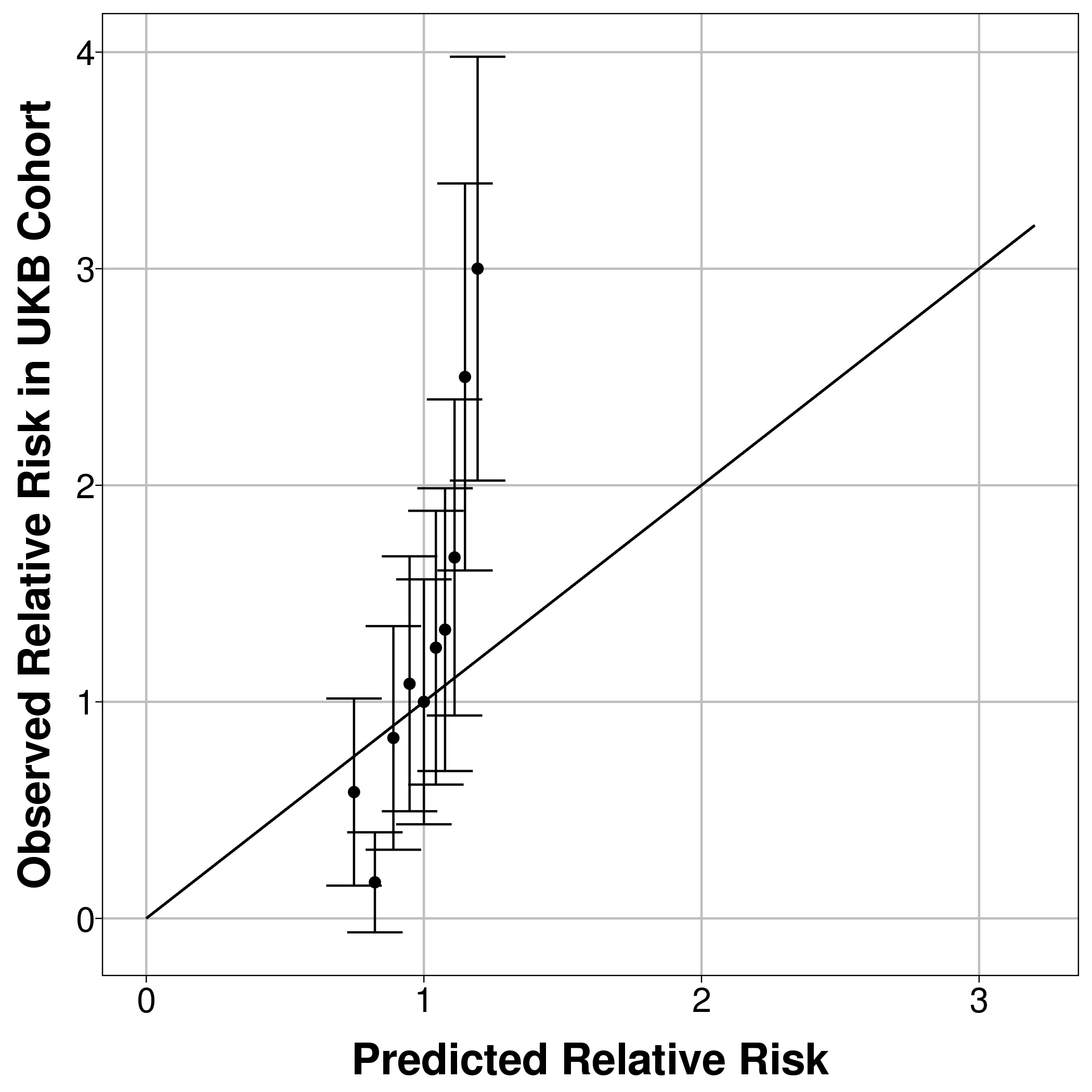 | 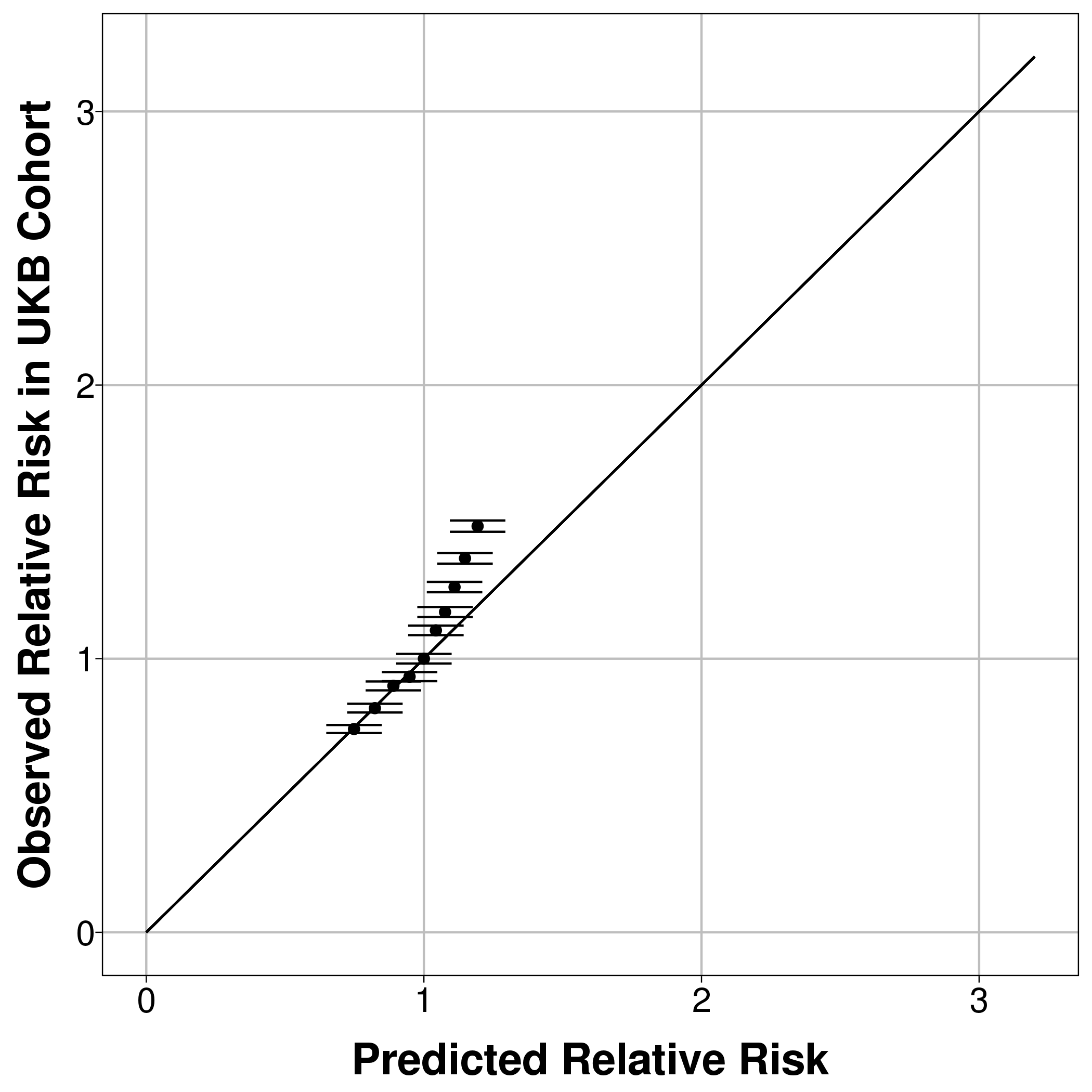 | 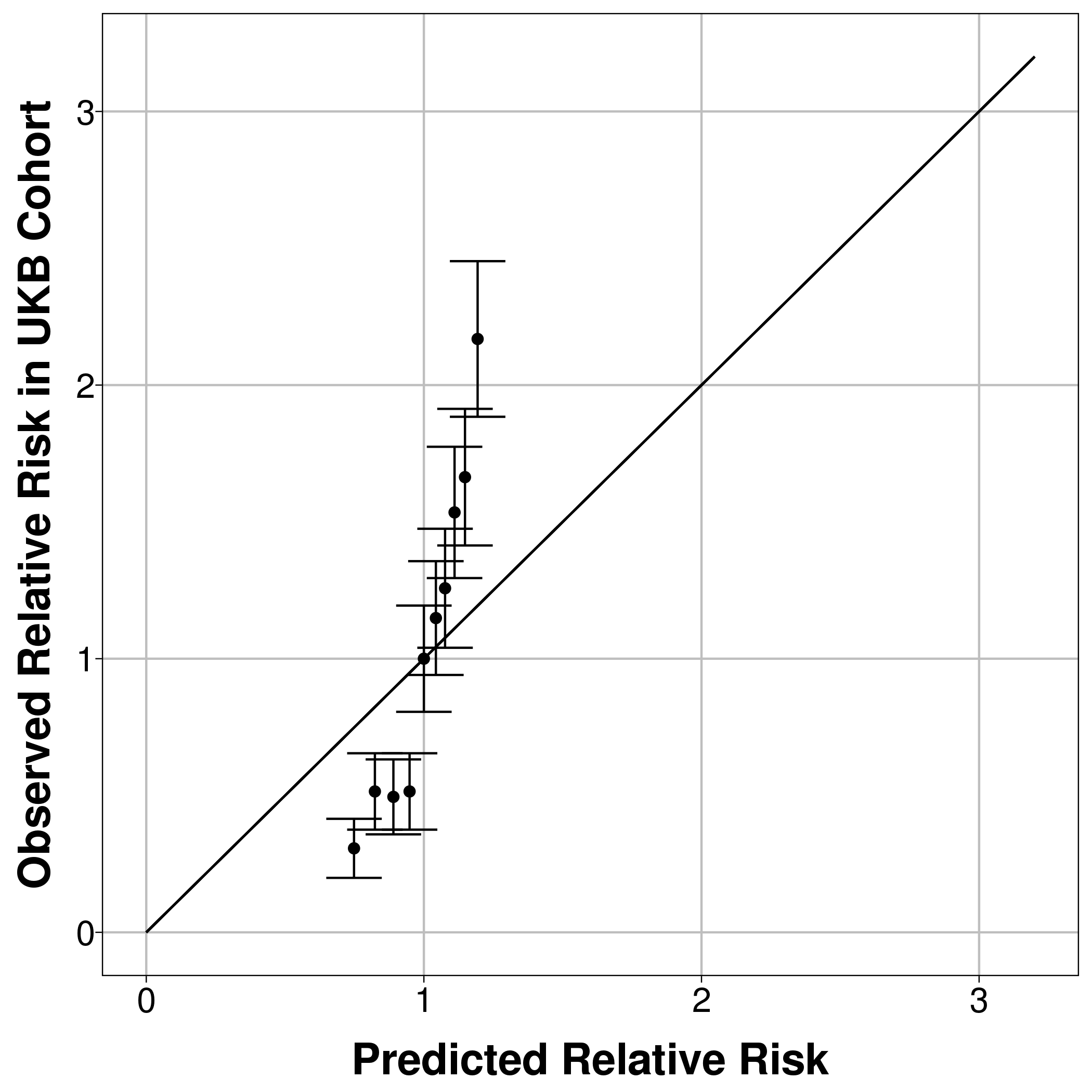 |
| Mortality CMS – short version with only 20 conditions (5 years follow-up) | 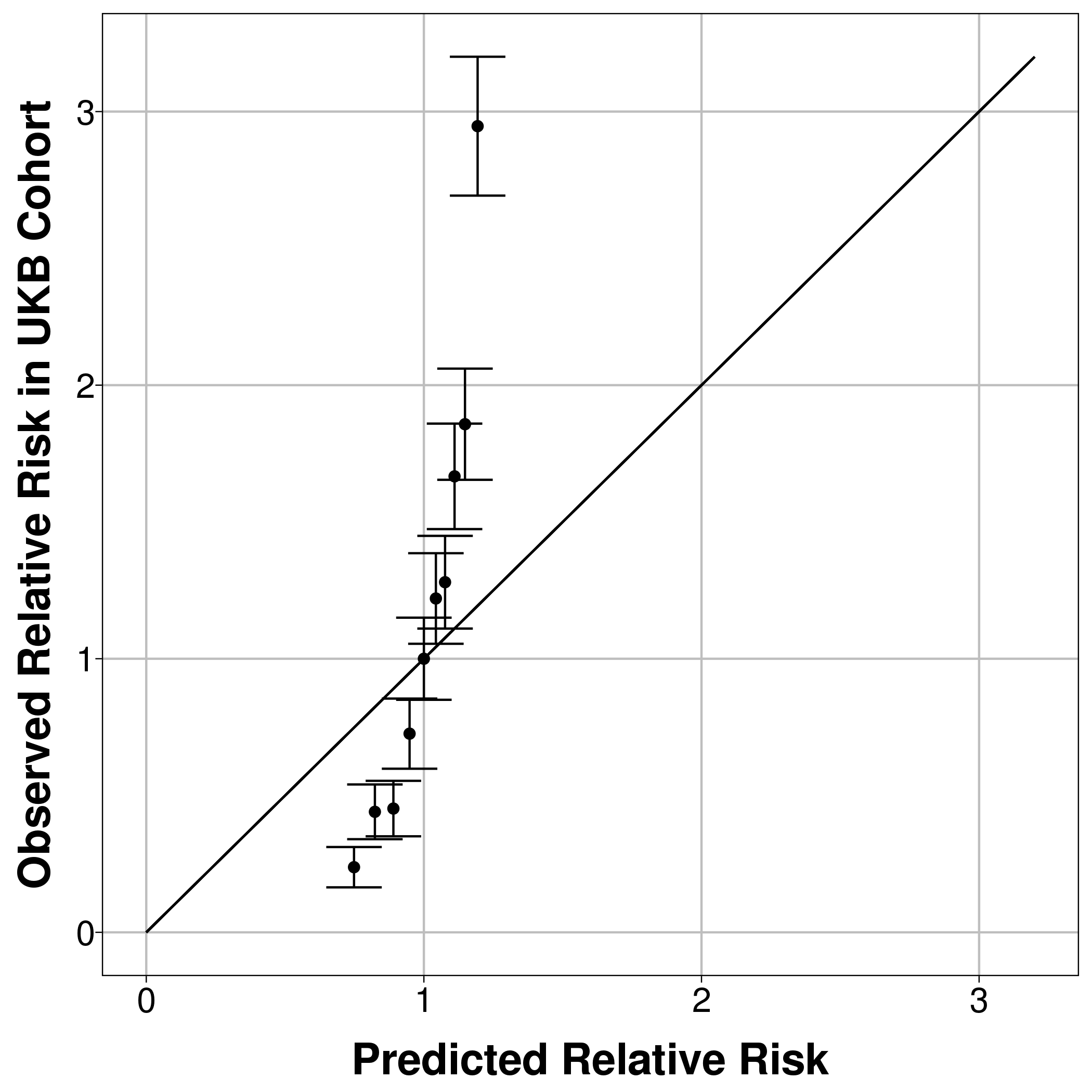 | 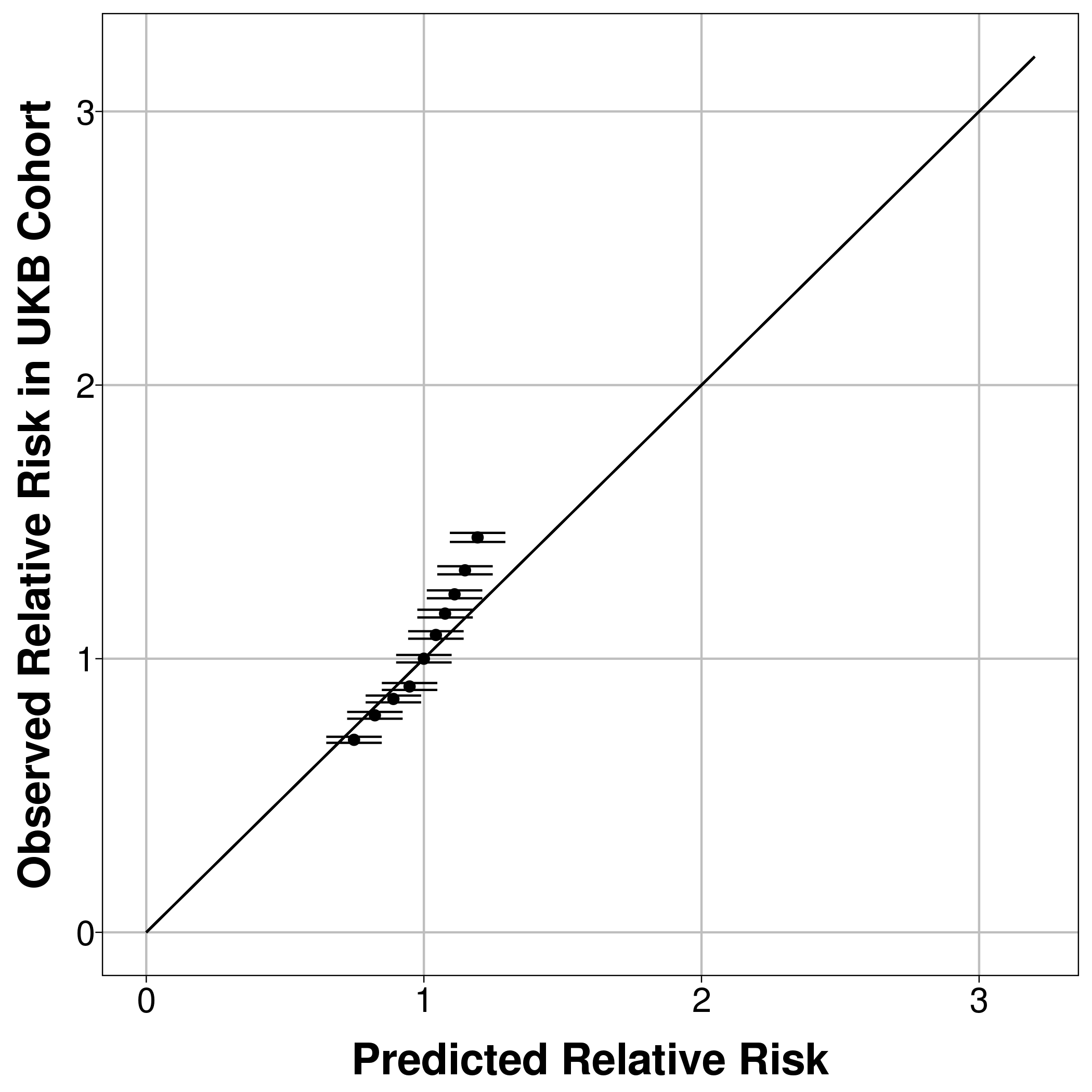 | 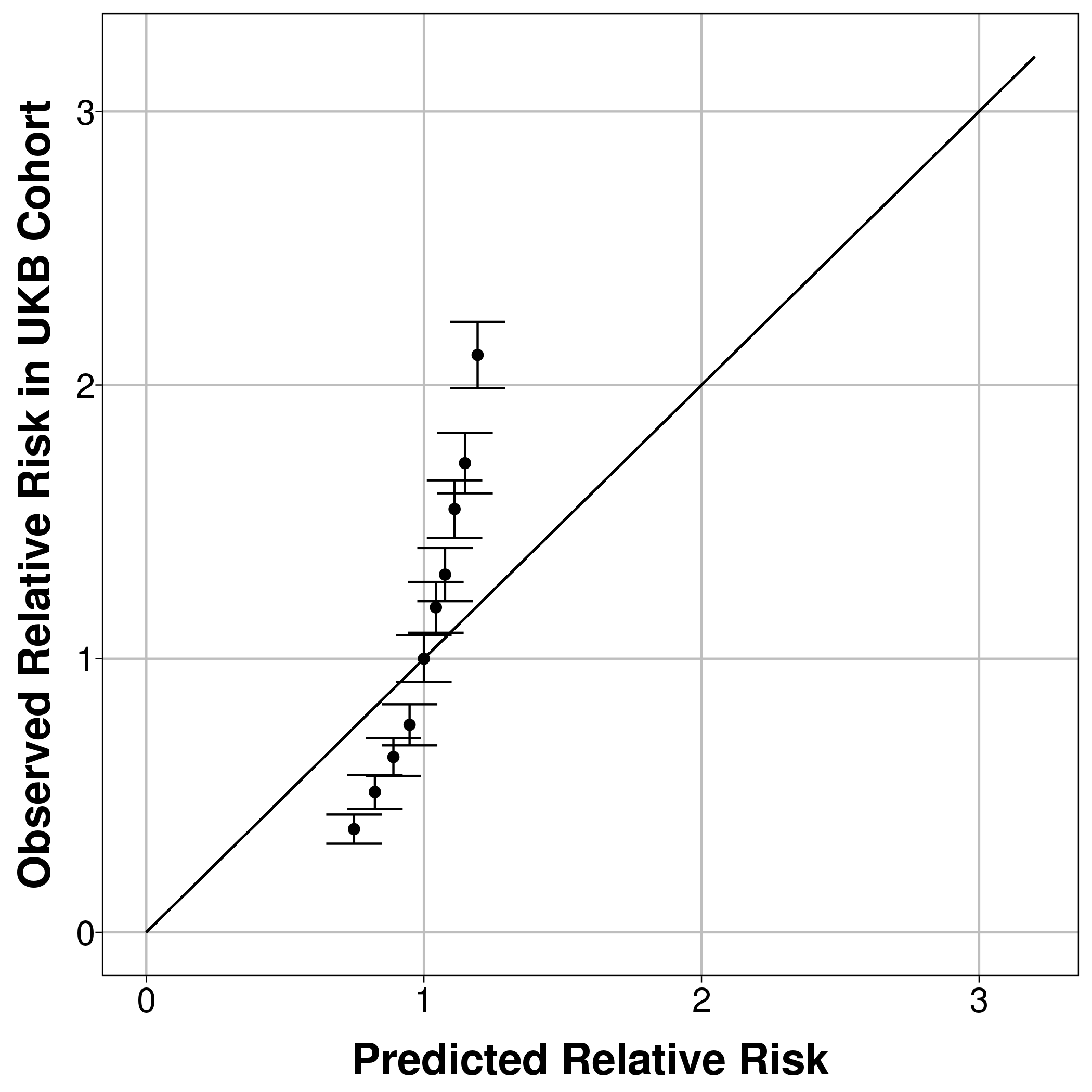 |
| Hospital CMS (1 year follow-up) | 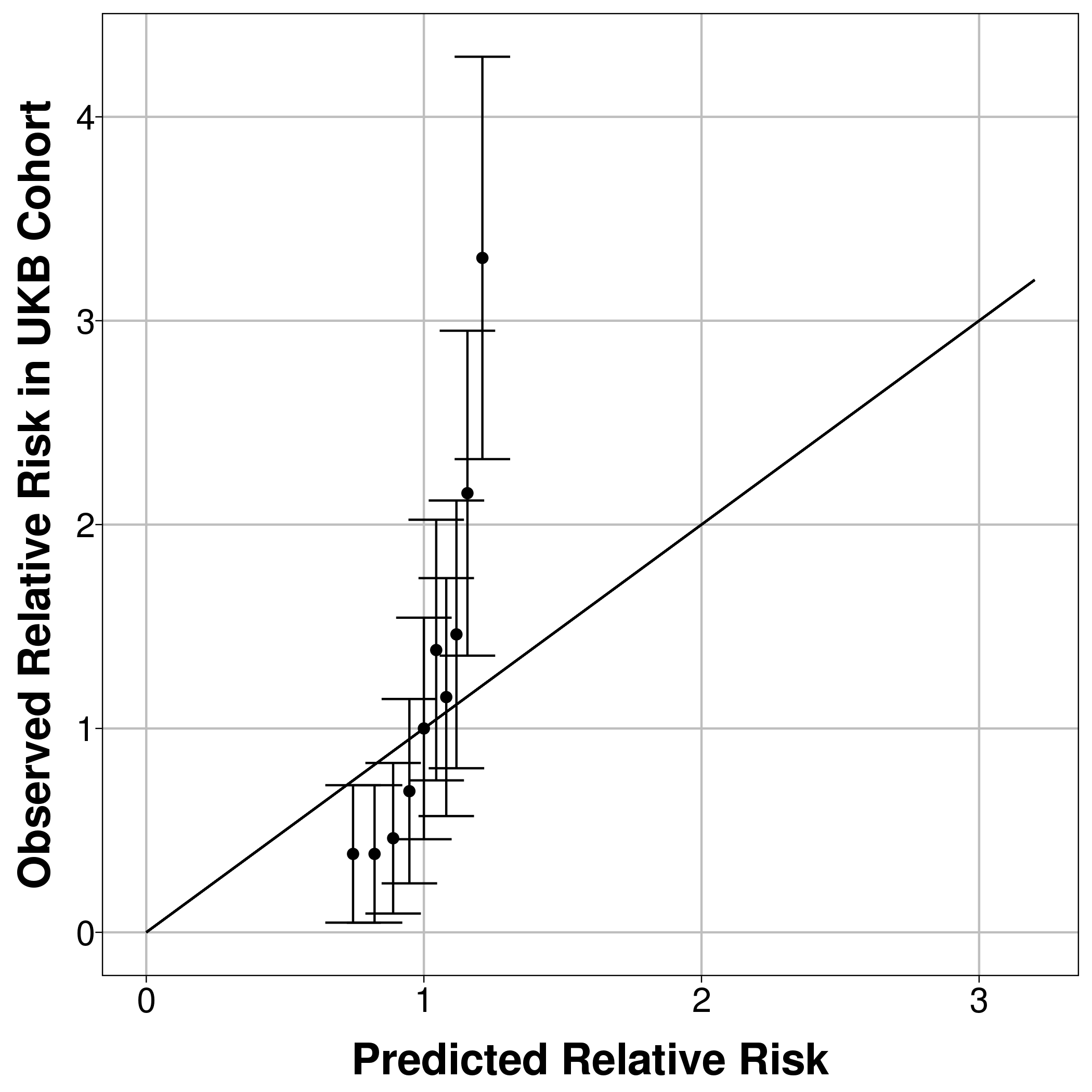 | 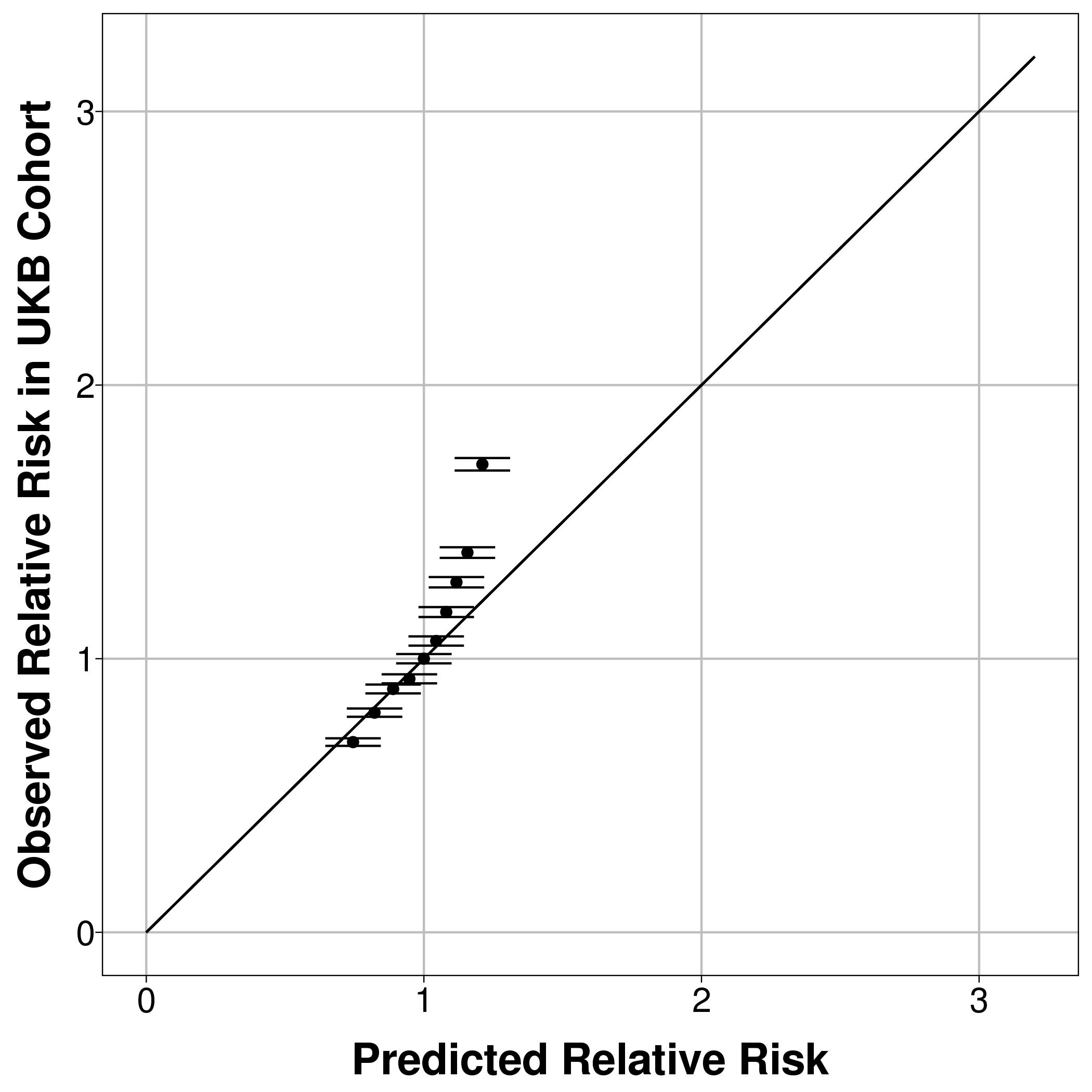 | 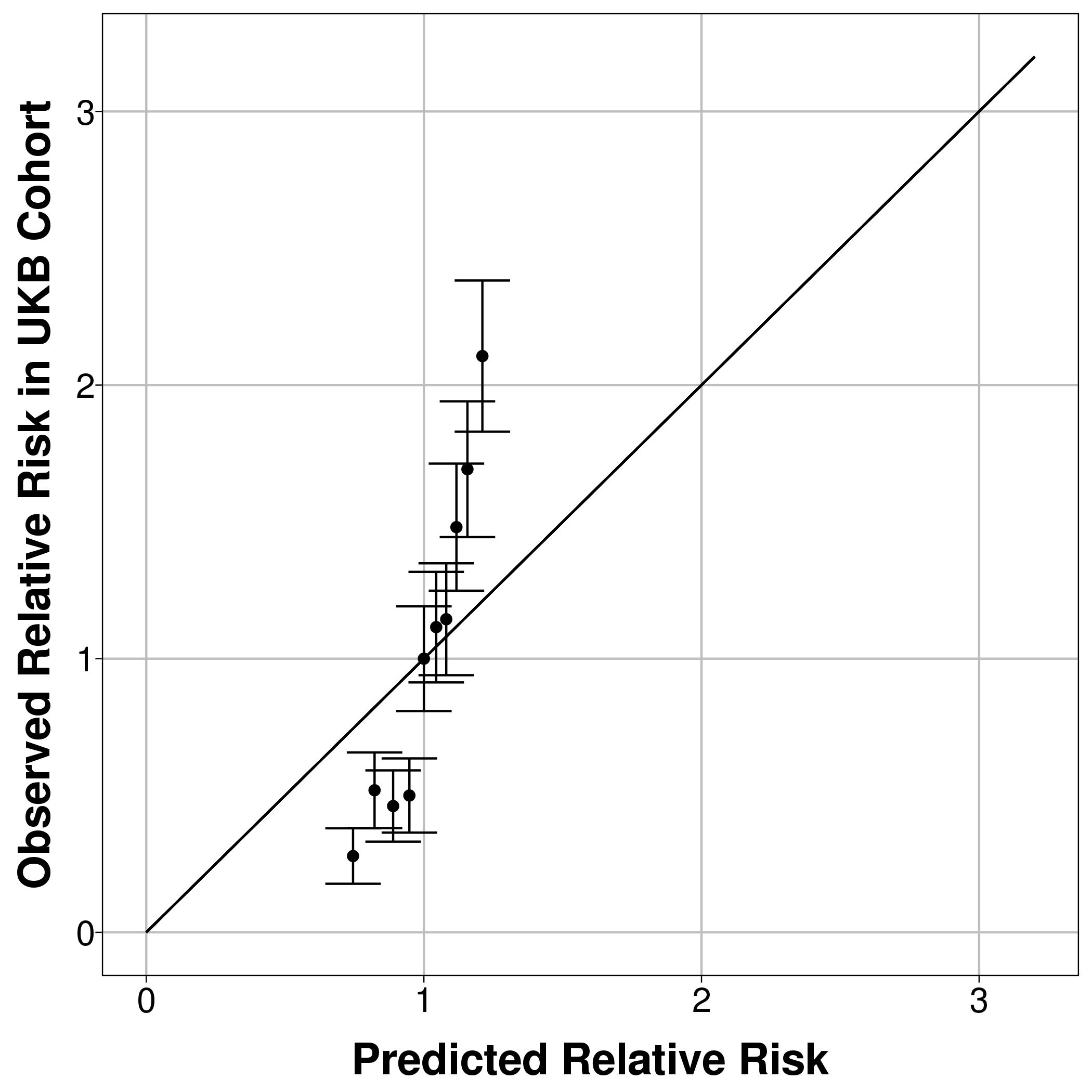 |
| Hospital CMS (5 years follow-up) | 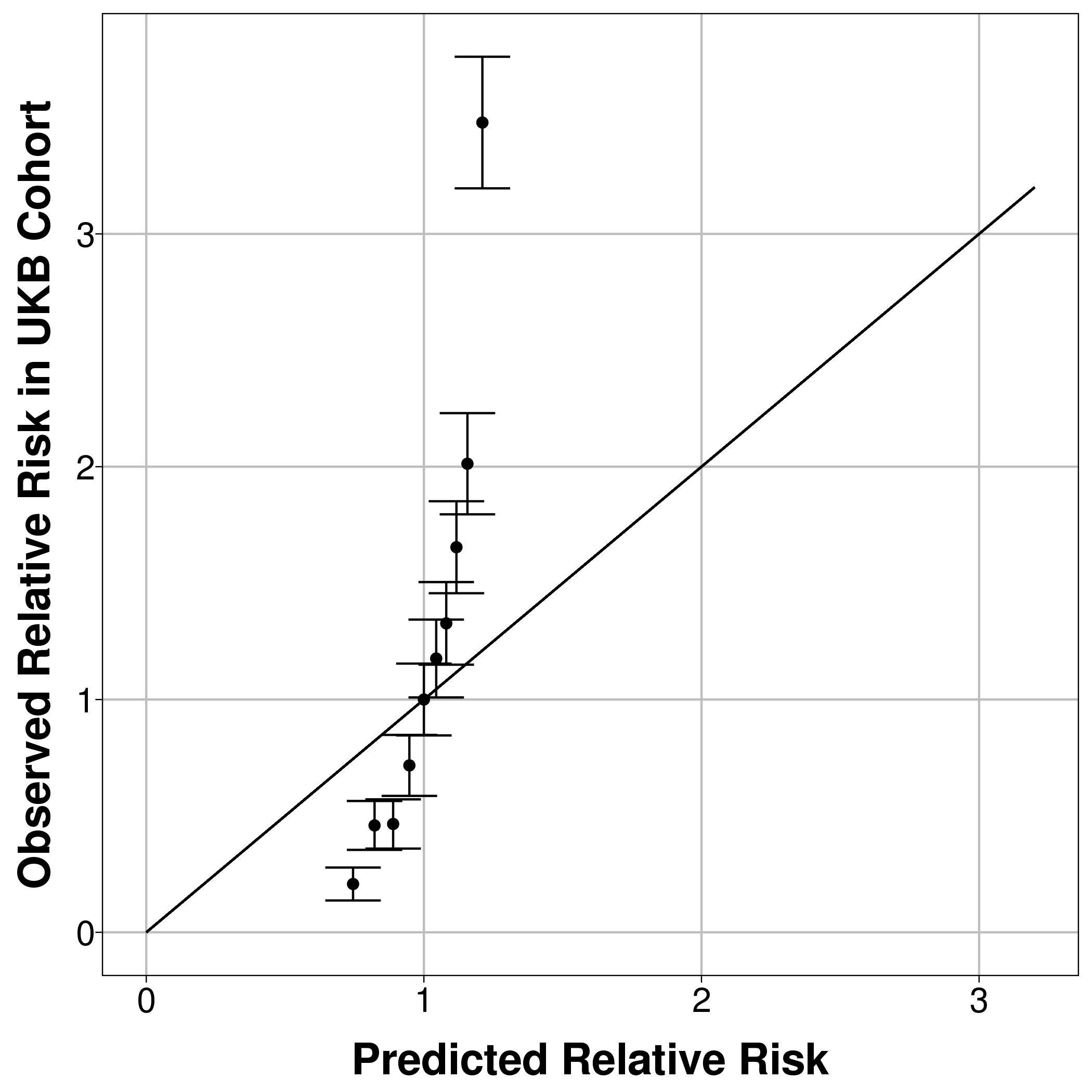 | 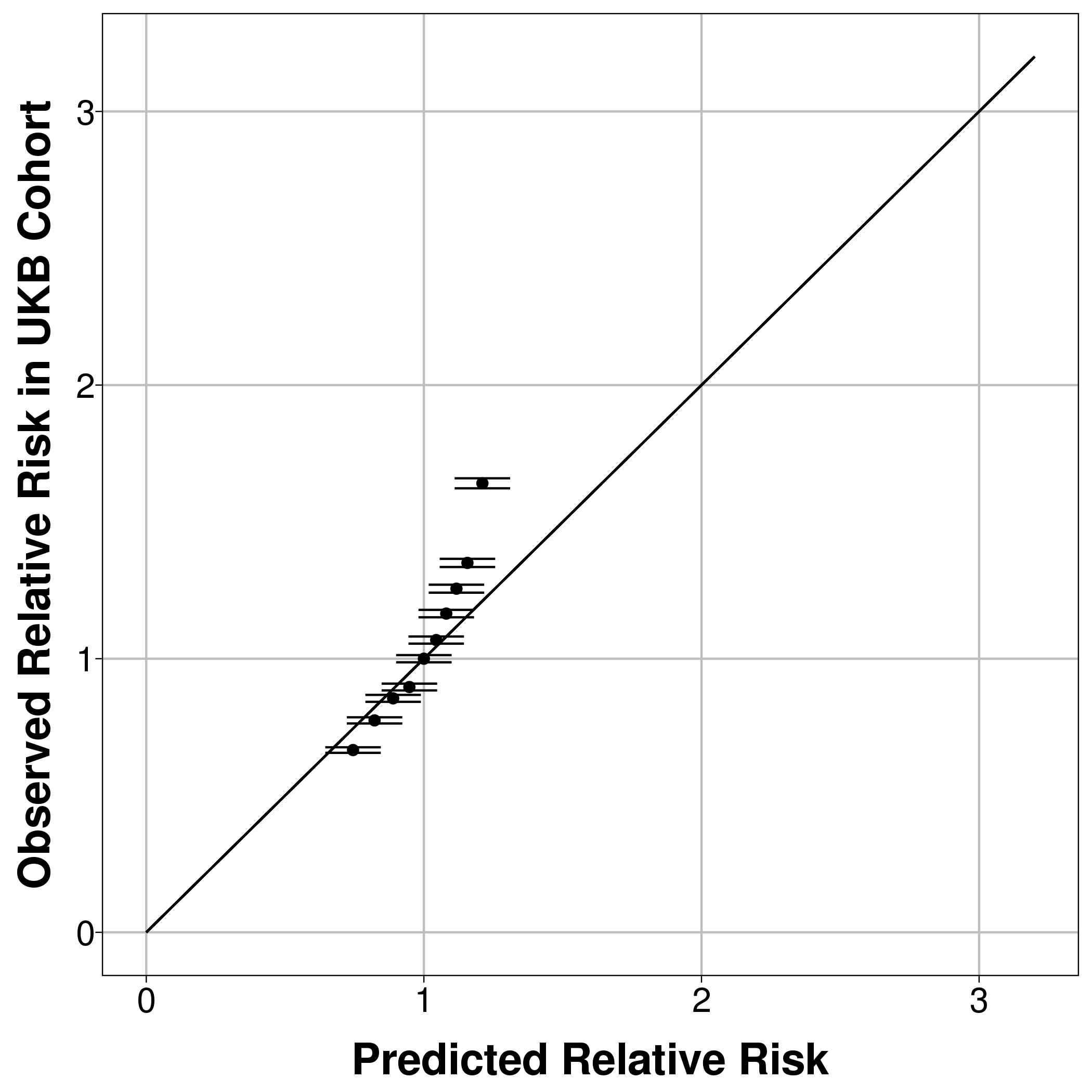 | 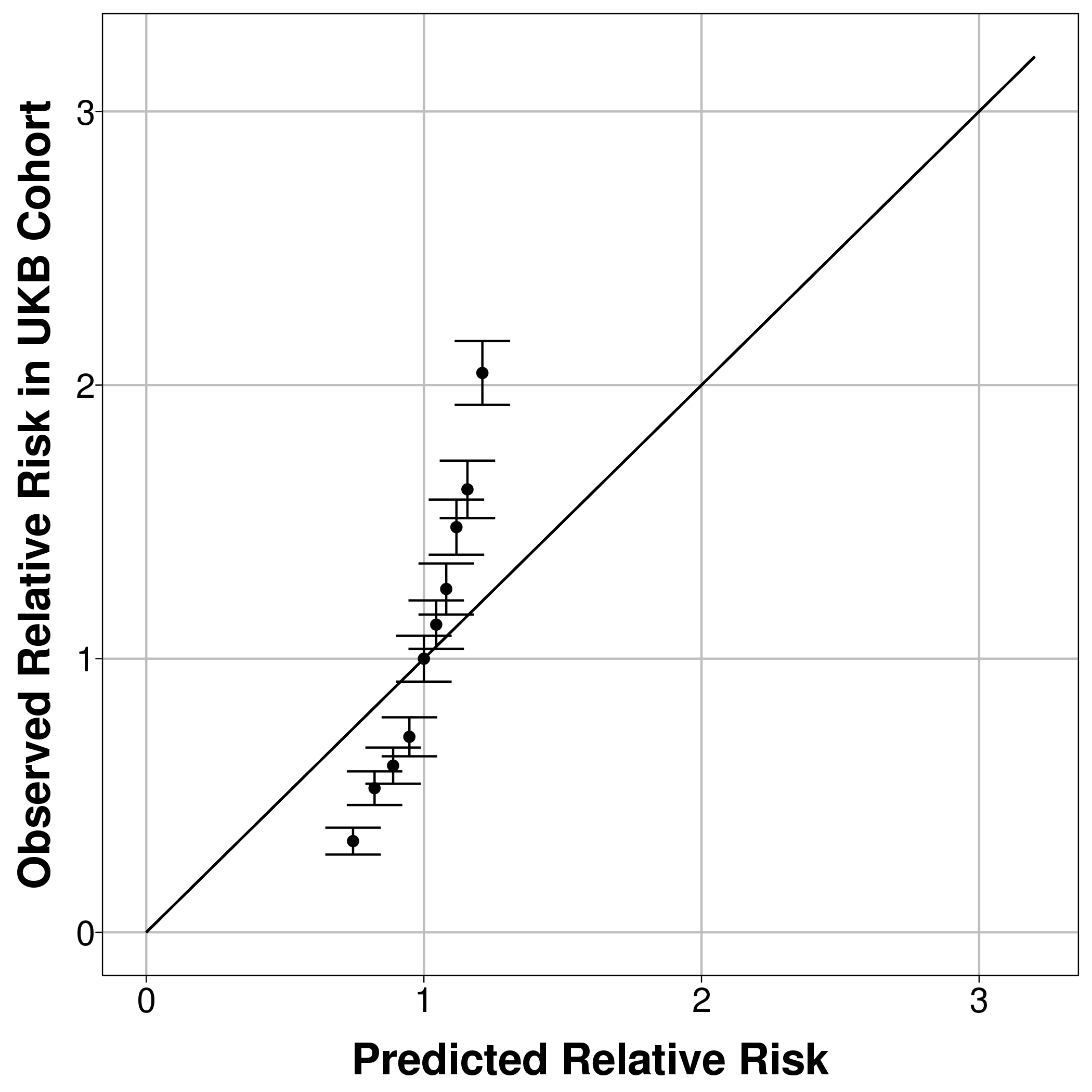 |
| Hospital CMS – short version with only 20 conditions (1 year follow-up) | 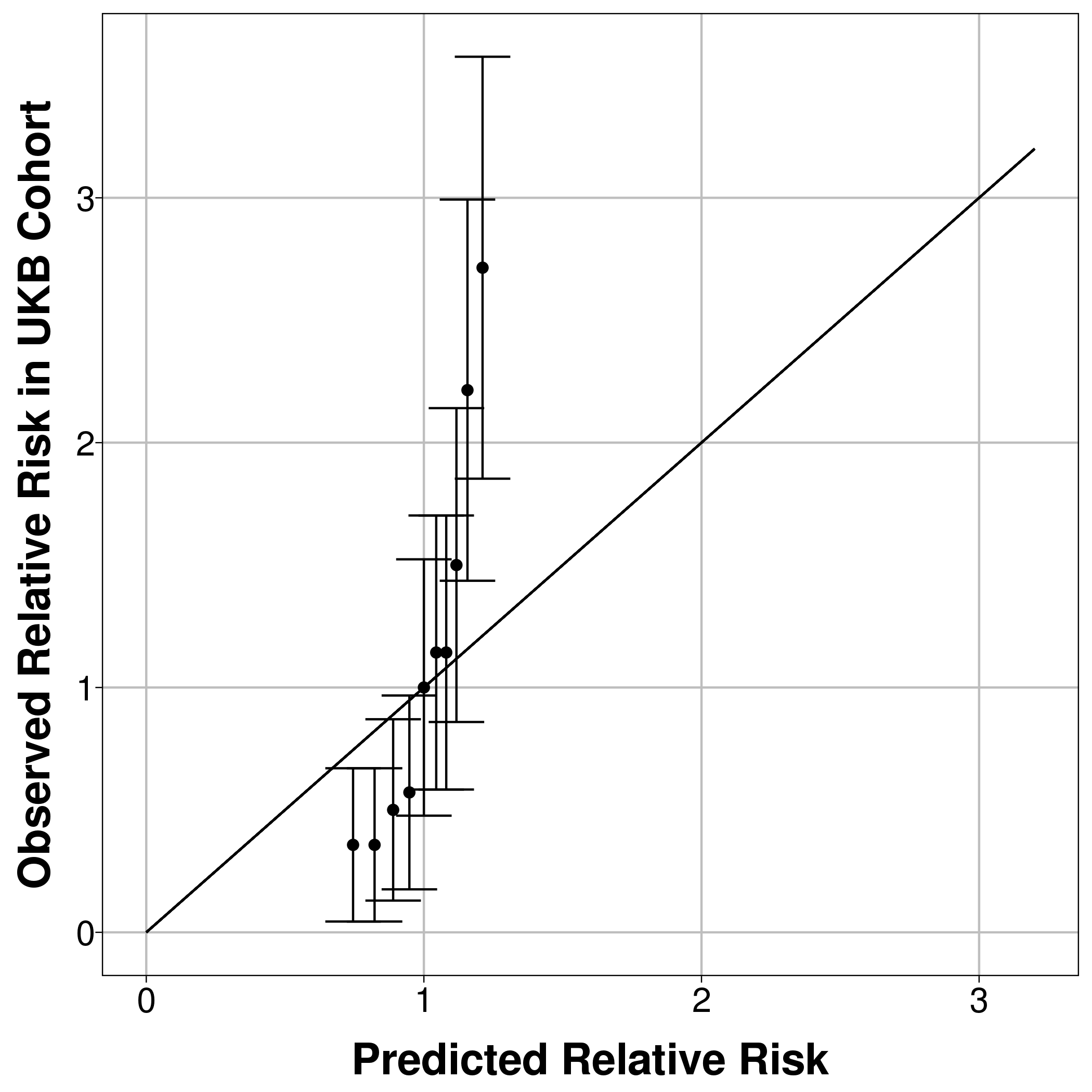 | 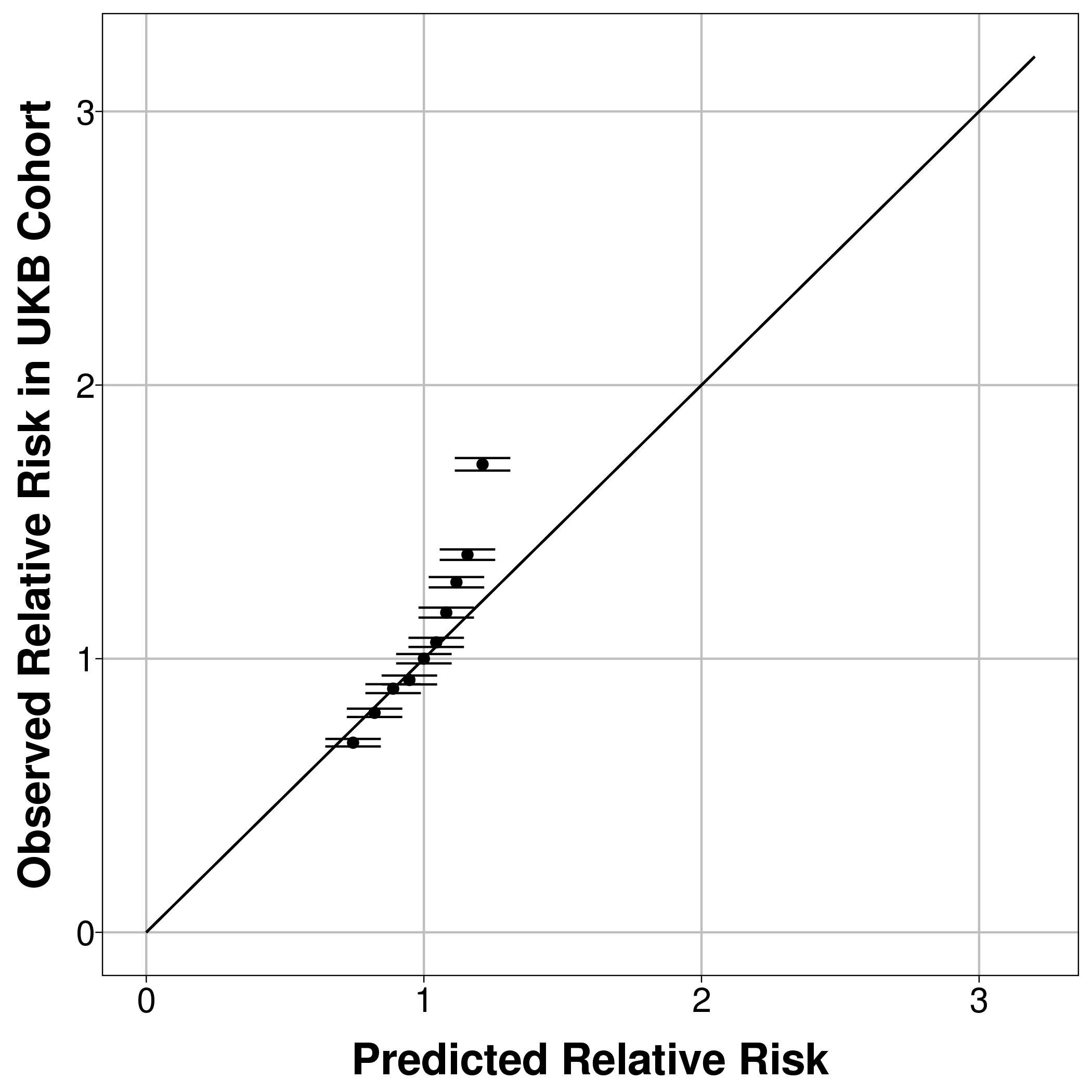 | 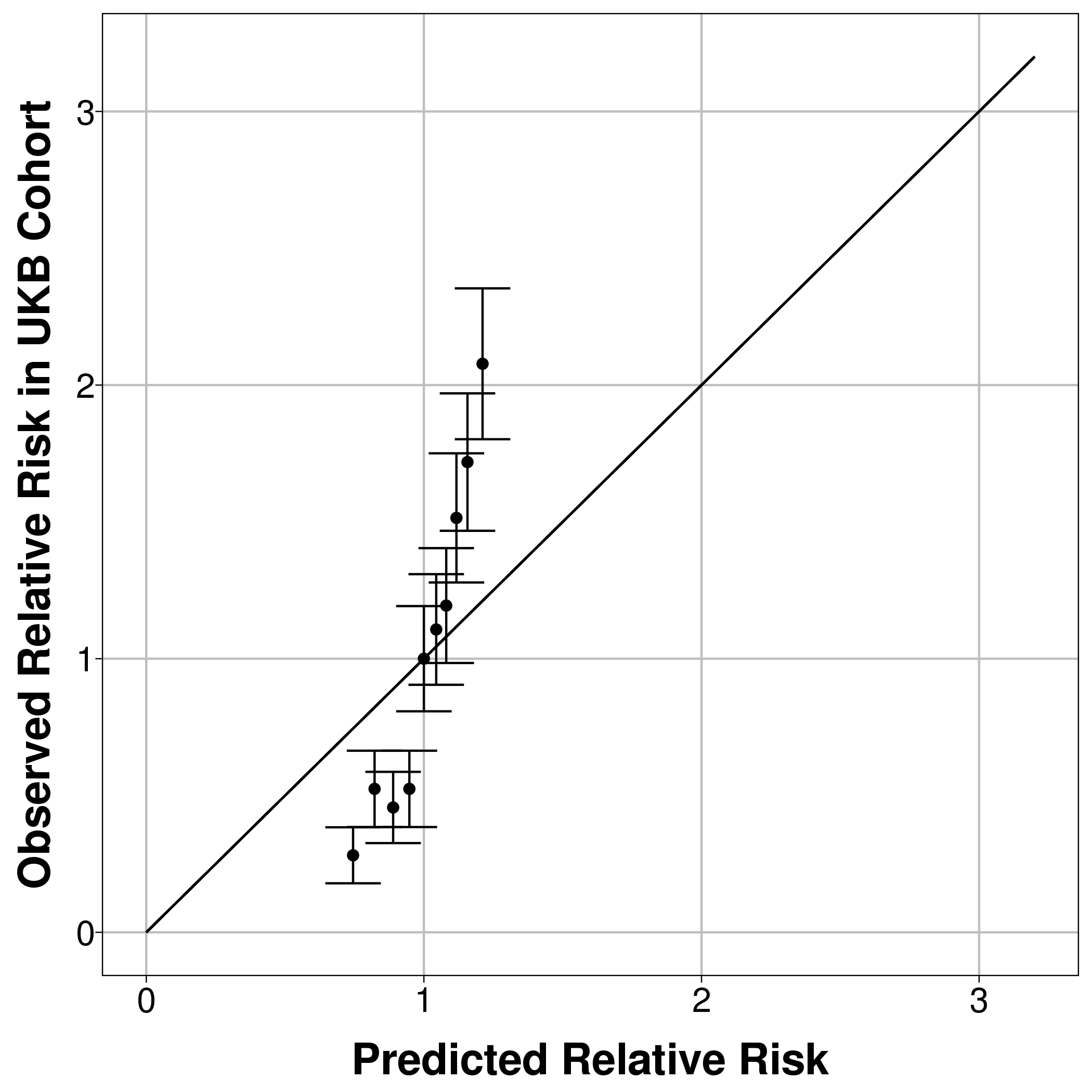 |
| Hospital CMS – short version with only 20 conditions (5 years follow-up) | 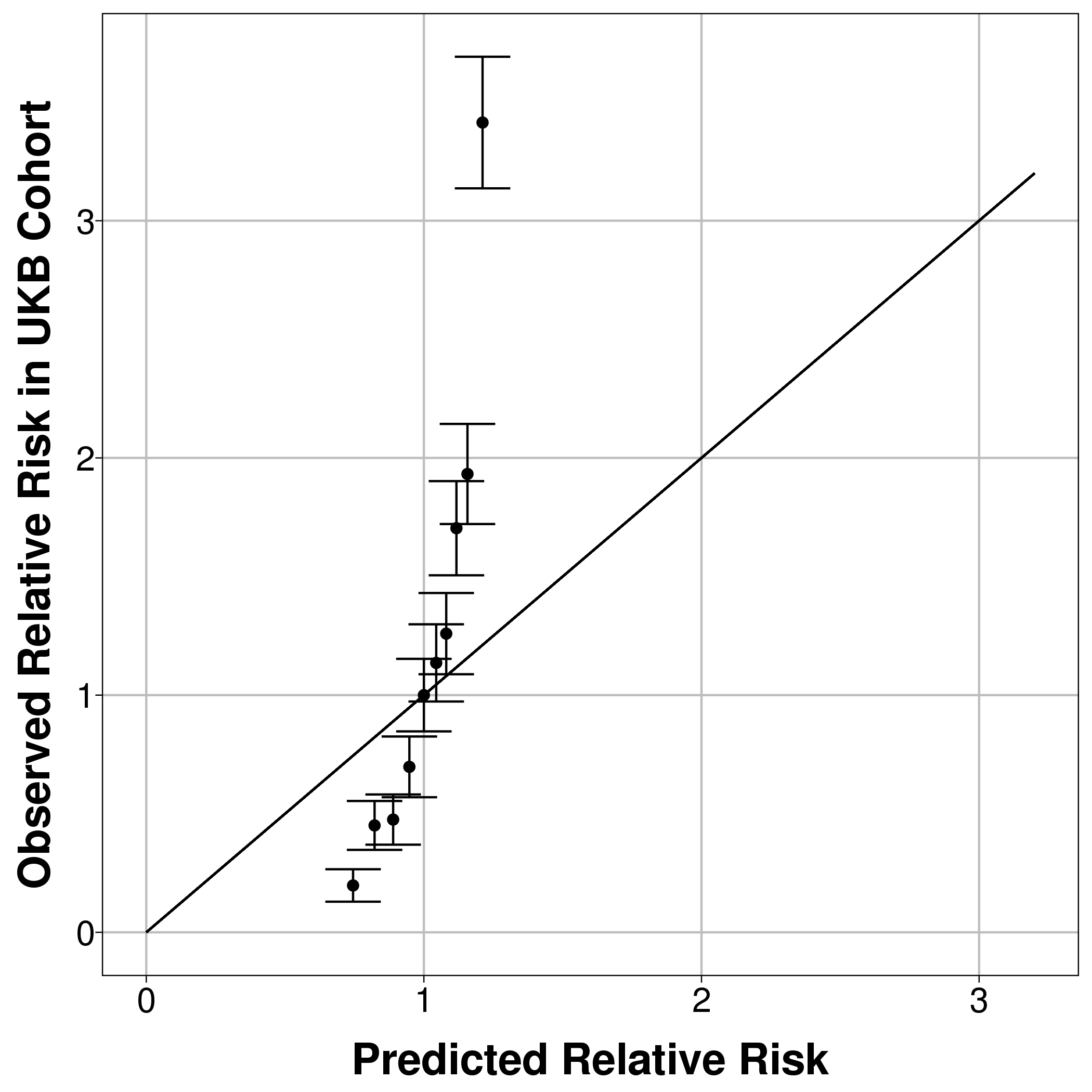 | 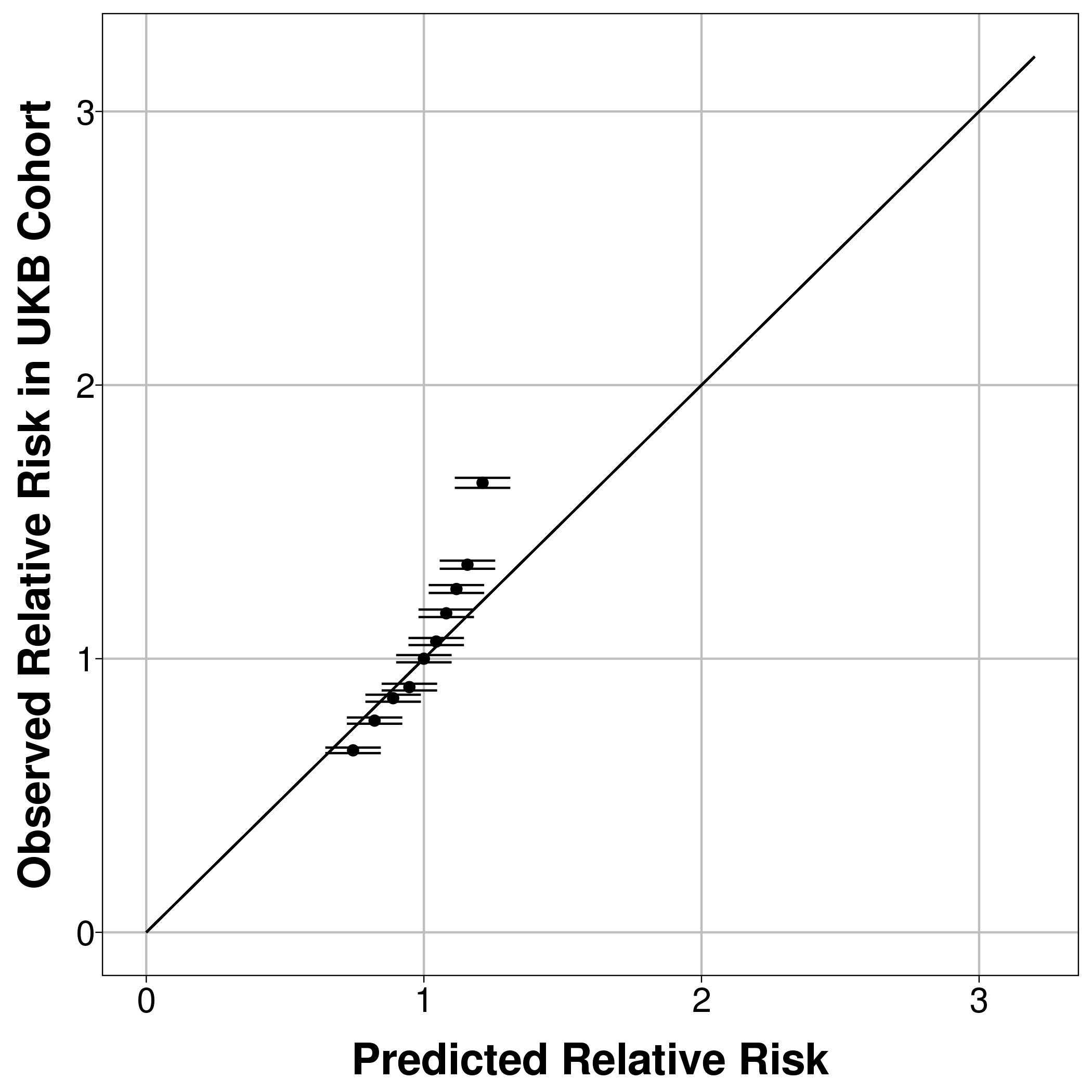 | 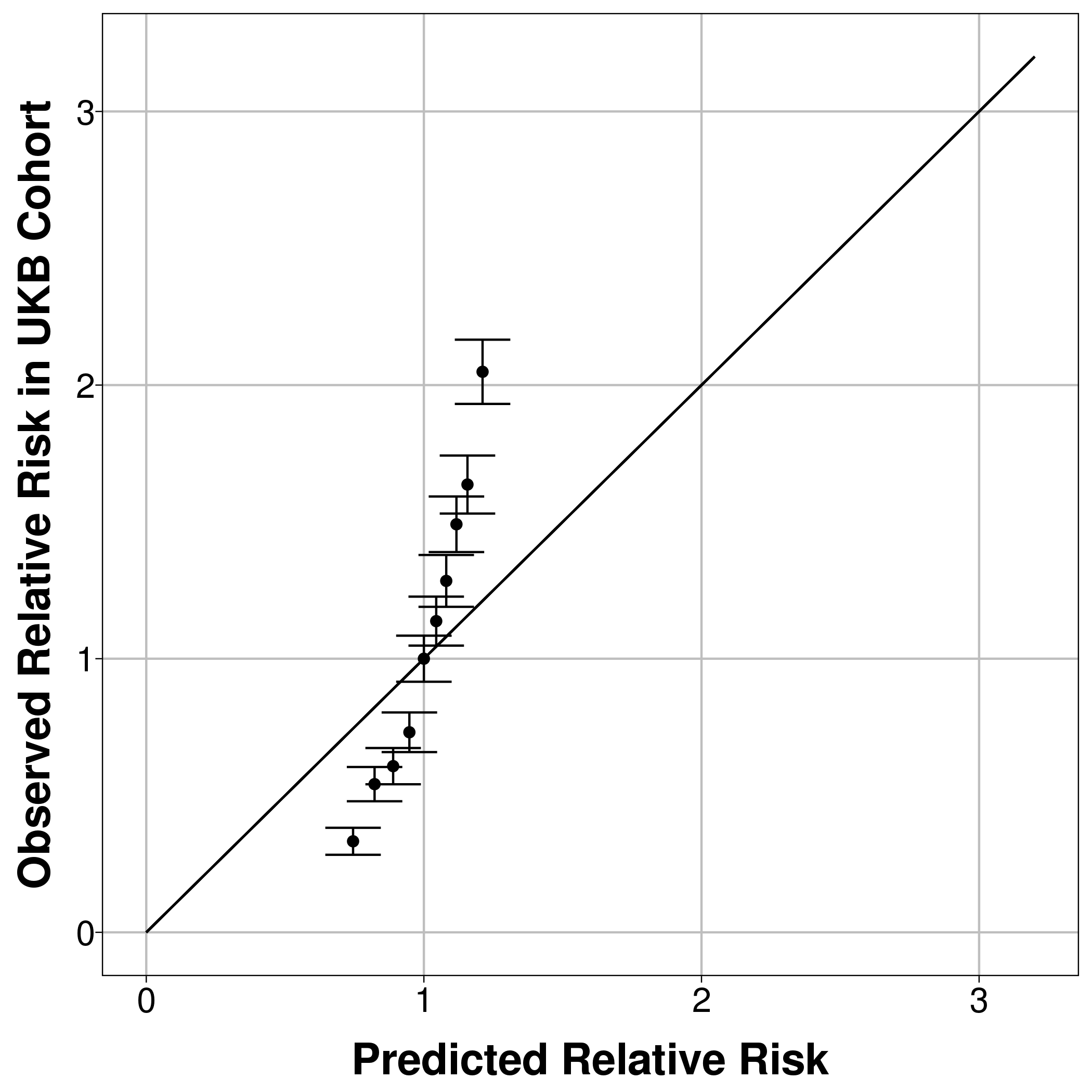 |

Figure S4 (a&b) – sensitivity analysis for the cancer diagnosis outcome


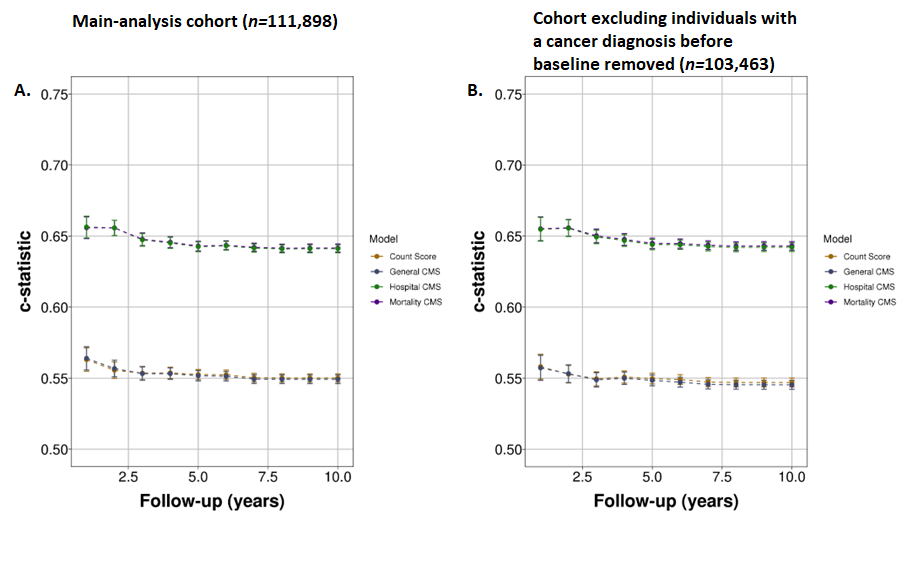
*Comparing discrimination for the cancer diagnosis outcome using (a) the main analysis cohort and using (b) a cohort which excludes those individuals (n=8,435) diagnosed with cancer (except non-melanoma skin cancer) before baseline assessment.*
