# Supplementary material for: Implementation and external validation of the Cambridge Multimorbidity Score in the UK Biobank cohort
Source: BMC Med Res Methodol. 2024 Mar 20;24:71. doi: 10.1186/s12874-024-02175-9 (PMC10953059; doi:10.1186/s12874-024-02175-9)
Supplement: Supplementary file 2 — Supplementary material 2. [file 12874_2024_2175_MOESM2_ESM.docx]

**Supplementary Methods**

*Codelist Development*


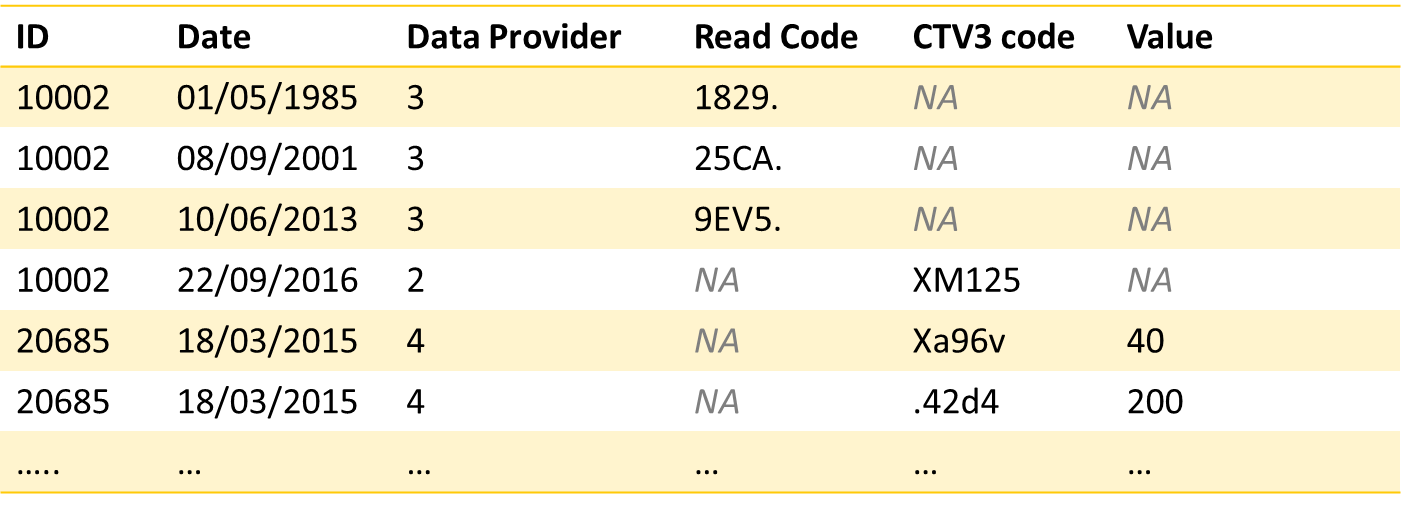
The primary care data (both GP clinical records and prescription record) for the UKB is provided in a row-per-event format, where each individual may appear in the dataset multiple times, for different clinical events (see dummy data in Fig. S4). For each event, the dataset provides the participant ID, the date the event was recorded, the data provider (see Table S6) and a code that defines the event type. By using a list that includes codes that describe a certain type of clinical occurrence (for example, hypertension or asthma), known as a “codelist”, the datasets can be queried to identify all events of that type.

Figure S4: dummy data extract from UKB GP clinical records

These coded clinical events include symptoms, diagnoses and test results (in the GP clinical records) as well as medications prescribed (prescription records). Some events (for example, 451F. – “glomerular filtration rate”) also include a value field, which may provide additional useful information. The data provided by UKB goes back as far as the 1980s for some participants. The most recent records provided are from 2016-2017 (last linkage date varies by data provider, see Table S6).

We took codes for the 37 conditions used in the Cambridge multimorbidity score from the codelists published from the score development study [1], which are available online from CPRD@Cambridge[2]. These codelists were developed by Payne et al. [1] for use with the Clinical Practise Research Dataset (CPRD) and use the readv2 coding framework for GP clinical records and Prodcodes for prescription records. To use these codelists to query the UKB primary care records, they needed to be converted into the range of different coding frameworks used by the four distinct data providers (see Table S6).

The majority of the 37 conditions were defined by events recorded in GP clinical records (for example, a diagnosis of hypertension or a recorded instance of alcohol abuse). Although three of the four data providers used the same coding framework as the CPRD dataset used in the development, the largest (English TPP) used the CTV3 coding framework. The original readv2 codelist was converted to CTV3 using the conversion table provided by UKB[3]. Subsequently the resulting combined readv2 and CTV3 codelist was manually checked to ensure consistency and identify any codes incorrectly included for each of the conditions.

A small number of the 37 conditions were defined using prescription records, either in combination with the GP clinical records (anxiety/depression, asthma, epilepsy, irritable bowel syndrome, migraines, painful conditions, psoriasis/eczema) or alone (constipation, schizophrenic/bipolar disorder). We followed the same process for converting the medications codelists as described above for the GP clinical records, however, given the larger number of coding frameworks involved this was a more complex process. The conversions carried out are shown in Fig. S5. Conversion tables from Prodcodes to DM+D and BNF (10 character) were taken from the product dictionary provided by CPRD[4]. Subsequently the UK Biobank conversion table for BNF to readv2 was used[3], and BNF codes were also reformatted to align with the different systems. Following conversion and reformatting the resulting medication codelists were manually checked to minimise inconsistency between data providers and identify any codes
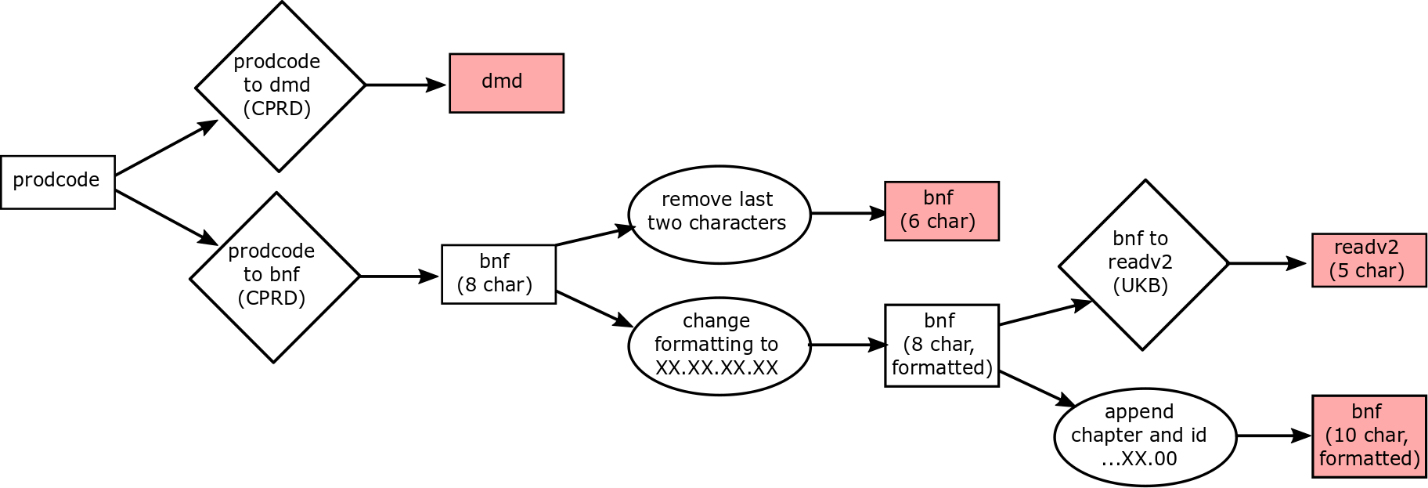
incorrectly included for each of the conditions.

Figure S5: Conversion and reformatting process for medication codelists

The final codelists used for all 37 conditions in this analysis as well as the R code used for their processing (conversion and reformatting) are included in the public GitHub repository for this project.

*Assessing Calibration*

Calibration of all of the models included in the analysis, for all three outcomes (death, GP consultation rate and cancer diagnosis), was assessed graphically. The risk predicted by the CMS and the relative risk of the outcome (death, cancer diagnosis or the frequency of GP consultation) were compared for both 1-year and 5-year follow-up.

We stratified our analysis cohort by deciles of predicted relative risk; the 5^th^ decile was used as baseline. The observed relative risk in each decile was calculated as the number of cases in that decile, divided by the number of individuals in that decile (for the outcome of GP consultation rate the mean consultation rate is used), divided by the same estimate in the 5^th^ decile. The predicted risk is the mean risk predicted by the model being assessed for the individuals in that decile and is also divided by the same estimate in the 5^th^ decile.

In all of the calibration plots, the line showing ideal calibration (equal observed and expected risk) is plotted. The 95% confidence intervals of the observed risk estimate are also given.

1. Payne, R.A., et al., *Development and validation of the Cambridge Multimorbidity Score.* CMAJ : Canadian Medical Association journal = journal de l'Association medicale canadienne, 2020. **192**(5): p. E107-E114.

2. CPRD@Cambridge. *CPRD at Cambridge*. Available from: <https://www.phpc.cam.ac.uk/pcu/research/research-groups/crmh/cprd_cam/>.

3. UK Biobank. *Resource 592: Clinical coding classification systems and maps*. Available from: <https://biobank.ndph.ox.ac.uk/showcase/refer.cgi?id=592>.

4. Clinical Practice Research Datalink. *PRODDICT: CPRD (GPRD) Product dictionary*. 28/02/2020; Available from: <https://rdrr.io/rforge/CALIBERcodelists/man/PRODDICT.html>.
