# Supplementary material for: Implementation and external validation of the Cambridge Multimorbidity Score in the UK Biobank cohort
Source: BMC Med Res Methodol. 2024 Mar 20;24:71. doi: 10.1186/s12874-024-02175-9 (PMC10953059; doi:10.1186/s12874-024-02175-9)
Supplement: Supplementary file 3 — Supplementary material 3. [file 12874_2024_2175_MOESM3_ESM.docx]

Table S1 – Prevalence of 37 conditions used in the CMS in both the development and validation cohorts

| **Conditions** | **Abbrv** | **Number in validation cohort** | **Prevalence in validation cohort (%)** | **Prevalence in CPRD development cohort (%) as reported by Payne et al.** | **Used in short versions of the CMS (20 conditions)** |
| --- | --- | --- | --- | --- | --- |
| Painful condition | PNC | 18783 | 16.79 | 11.63 | x |
| Hypertension | HYP | 18426 | 16.47 | 19.24 | x |
| Anxiety/Depression | ANX, DEP | 10903 | 9.74 | 12.85 | x |
| Irritable bowel syndrome | IBS | 6882 | 6.15 | 7.61 | x |
| Thyroid disorders | THY | 6401 | 5.72 | 5.24 |  |
| Hearing loss | HEL | 5893 | 5.27 | 11.27 | x |
| Diabetes | DIB | 5233 | 4.68 | 6.58 | x |
| Coronary heart disease | CHD | 4328 | 3.87 | 4.79 | x |
| Chronic kidney disease | CKD | 2847 | 2.54 | 4.50 | x |
| Constipation | CON | 2161 | 1.93 | 2.67 | x |
| Diverticular disease | DIV | 1937 | 1.73 | 3.24 |  |
| Schizophrenia (and related non-organic psychosis) or bipolar disorder | SCZ | 1725 | 1.54 | 0.98 | x |
| Asthma | AST | 1587 | 1.42 | 7.20 | x |
| Prostate Disorders | PRO | 1279 | 1.14 | 6.31 |  |
| Chronic sinusitis | SIN | 1247 | 1.11 | 2.96 |  |
| Stroke & TIA | STR | 1171 | 1.05 | 2.55 |  |
| Psoriasis or eczema | PSO | 993 | 0.89 | 1.16 |  |
| Rheumatoid arthritis, other inflammatory polyarthropathies & systematic connective tissue disorders | RHE | 966 | 0.86 | 2.33 | x |
| Inflammatory bowel disease | IBD | 922 | 0.82 | 0.96 |  |
| Migraine | MIG | 915 | 0.82 | 0.51 |  |
| COPD | COP | 677 | 0.61 | 2.46 | x |
| Peptic ulcer disease | PEP | 626 | 0.56 | 1.62 |  |
| Atrial fibrillation | ATR | 527 | 0.47 | 2.72 | x |
| Peripheral vascular disease | PVD | 427 | 0.38 | 0.88 |  |
| Heart failure | HEF | 386 | 0.35 | 1.04 | x |
| Alcohol problems | ALC | 340 | 0.30 | 1.60 | x |
| Blindness and low vision | BLI | 340 | 0.30 | 1.08 |  |
| Epilepsy | EPI | 289 | 0.26 | 0.97 | x |
| Chronic Liver Disease and Viral Hepatitis | CLD | 224 | 0.20 | 0.53 |  |
| Cancer | CAN | 216 | 0.19 | 2.15 | x |
| Substance Misuse | PSM | 160 | 0.14 | 1.19 |  |
| Anorexia or bulimia | ANO | 51 | 0.05 | 0.55 |  |
| Multiple sclerosis | MSC | 24 | 0.02 | 0.28 |  |
| Dementia | DEM | 17 | 0.02 | 1.02 | x |
| Learning disability | LEA | 14 | 0.01 | 0.47 |  |
| Bronchiectasis | BRO | 0 | 0.00 | 0.43 |  |
| Parkinson's disease | PRK | 0 | 0.00 | 0.28 |  |

Table S2 – comparison of whole UKB cohort to both the subset with available primary care records and the validation cohort using only individuals with English TPP records.

|  | | **UK Biobank Cohort** | **Available Primary Care Records** | **Validation Cohort** |
| --- | --- | --- | --- | --- |
| **Counts** | **n (%)** | 502413 (100) | 161482 (32.1) | 111898 (22.3) |
| **Cohort Characteristics** | | | | |
| **Age** | **Mean (SD)** | 56.5 (8.1) | 56.7 (8.1) | 57.0 (8.0) |
|  | **Missing (%)** | 0 (0) | 0 (0) | 0 (0) |
| **Sex** | **Female (%)** | 273328 (54.4) | 88048 (54.5) | 60036 (53.7) |
|  | **Male (%)** | 229085 (45.6) | 73434 (45.5) | 51862 (46.3) |
|  | **Missing (%)** | 0 (0) | 0 (0) | 0(0) |
| **Smoking Status** | **Never (%)** | 273478 (54.4) | 88062 (54.5) | 61638 (55.1) |
|  | **Former (%)** | 173025 (34.4) | 56147 (34.8) | 38845 (34.7) |
|  | **Current (%)** | 52962 (10.5) | 16382 (10.1) | 10864 (9.7) |
|  | **Missing (%)** | 2948 (0.59) | 891 (0.55) | 551 (0.49) |
| **Deprivation Quintiles (English IMD)** | **1 (least deprived)** | 124425 (24.8) | 41449 (26.7) | 29789 (26.6) |
|  | **2** | 100215 (19.9) | 36671 (22.7) | 26247 (23.5) |
|  | **3** | 80295 (16.0) | 31311 (19.4) | 22008 (19.7) |
|  | **4** | 70620 (14.1) | 26478 (16.4) | 18082 (16.2) |
|  | **5 (most deprived)** | 57095 (11.3) | 20188 (12.5) | 12460 (11.1) |
|  | **Missing (%)** | 69763 (13.9) | 5385 (3.3) | 3312 (3.0) |
| **BMI (kg/m^2^)** | **Mean (sd)** | 27.4 (4.8) | 27.5 (4.8) | 27.4 (4.7) |
|  | **<20 (%)** | 11375 (2.3) | 3496 (2.16) | 2444 (2.2) |
|  | **20 - 24.9 (%)** | 149464 (29.7) | 47314 (29.3) | 33265 (29.7) |
|  | **25 - 29.9 (%)** | 206856 (41.2) | 67125 (41.6) | 46958 (42.0) |
|  | **≥ 30 (%)** | 118407 (23.6) | 39047 (24.2) | 26349 (23.5) |
|  | **Missing (%)** | 16311 (3.2) | 4500 (2.8) | 2882 (2.6) |
| **Outcome measures** | | | | |
| **Deaths** | **Number in 1 year (%)** | 967 (0.19) | 281 (0.17) | 161 (0.14) |
|  | **Number in 5 years (%)** | 8679 (1.7) | 2640 (1.6) | 1987 (1.8) |
| **Cancer Diagnoses (first in follow-up)** | **Number in 1 year (%)** | 4587 (0.91) | 1504 (0.93) | 1071 (0.96) |
|  | **Number in 5 years (%)** | 24735 (4.9) | 7921 (4.9) | 5589 (5.0) |
| **GP Consultation Rate** | **Rate per year, Median (IQR)** | - | 7.18 (4.05-11.44) | 7.68 (4.78-11.73) |
|  | **Zero consultations (%)** | - | 1453 (0.90) | 43 (0.04) |
| **GP Follow-up (years)** | **Median (IQR)** | - | 6.90 (6.15-7.55) | 7.03 (6.48-7.61) |

Table S3 – Discrimination (c-statistic) of the CMS ( long versions of the models including all 37 conditions only)

| **Primary analysis** | **Years of follow-up** | | | | | | | | | |
| --- | --- | --- | --- | --- | --- | --- | --- | --- | --- | --- |
|  | **1** | **2** | **3** | **4** | **5** | **6** | **7** | **8** | **9** | **10** |
| **Outcome: Death** | | | | | | | | | | |
| **Deaths (cumulative)** | 161 | 533 | 965 | 1464 | 1987 | 2620 | 3275 | 3937 | 4700 | 5503 |
| **General CMS** | 0.697 (0.676-0.719) | 0.69 (0.678-0.702) | 0.682 (0.673-0.691) | 0.668 (0.661-0.675) | 0.654 (0.648-0.66) | 0.649 (0.643-0.654) | 0.646 (0.642-0.651) | 0.646 (0.642-0.651) | 0.648 (0.643-0.652) | 0.645 (0.641-0.649) |
| **Mortality CMS** | 0.666 (0.646-0.687) | 0.686 (0.675-0.696) | 0.684 (0.675-0.692) | 0.684 (0.678-0.691) | 0.685 (0.679-0.69) | 0.688 (0.683-0.693) | 0.69 (0.686-0.694) | 0.691 (0.687-0.695) | 0.694 (0.69-0.698) | 0.696 (0.692-0.699) |
| **Hospital CMS** | 0.688 (0.668-0.708) | 0.705 (0.694-0.716) | 0.702 (0.694-0.71) | 0.7 (0.694-0.707) | 0.699 (0.693-0.704) | 0.701 (0.696-0.706) | 0.703 (0.698-0.707) | 0.703 (0.699-0.707) | 0.706 (0.702-0.71) | 0.707 (0.704-0.711) |
| **Unweighted Count Score** | 0.684 (0.663-0.705) | 0.681 (0.669-0.692) | 0.672 (0.664-0.681) | 0.658 (0.651-0.665) | 0.644 (0.638-0.65) | 0.639 (0.634-0.644) | 0.638 (0.633-0.643) | 0.638 (0.633-0.642) | 0.639 (0.635-0.643) | 0.637 (0.633-0.64) |
| **Outcome: GP Consultation Rate** | | | | | | | | | | |
| **Cohort Size** | 111166 | 110083 | 108737 | 107479 | 105726 | 102777 | 58192 | 10219 |  |  |
| **Median Rate** | 6.00 | 6.50 | 7.00 | 7.00 | 7.20 | 7.33 | 7.43 | 7.63 |  |  |
| **General CMS** | 0.654 (0.654-0.655) | 0.663 (0.662-0.664) | 0.665 (0.664-0.666) | 0.666 (0.665-0.667) | 0.667 (0.666-0.668) | 0.668 (0.667-0.669) | 0.667 (0.665-0.668) | 0.665 (0.663-0.668) |  |  |
| **Mortality CMS** | 0.599 (0.598-0.6) | 0.611 (0.61-0.612) | 0.617 (0.616-0.618) | 0.621 (0.62-0.622) | 0.625 (0.624-0.625) | 0.627 (0.626-0.628) | 0.629 (0.628-0.631) | 0.632 (0.629-0.635) |  |  |
| **Hospital CMS** | 0.617 (0.616-0.618) | 0.629 (0.628-0.63) | 0.636 (0.635-0.637) | 0.64 (0.639-0.641) | 0.643 (0.642-0.644) | 0.646 (0.645-0.647) | 0.648 (0.647-0.649) | 0.65 (0.647-0.653) |  |  |
| **Unweighted Count Score** | 0.655 (0.654-0.656) | 0.664 (0.663-0.664) | 0.666 (0.665-0.667) | 0.667 (0.666-0.668) | 0.668 (0.668-0.669) | 0.669 (0.668-0.67) | 0.668 (0.667-0.669) | 0.667 (0.664-0.669) |  |  |
| **Outcome: Cancer Diagnosis** | | | | | | | | | | |
| **Cases (cumulative)** | 1071 | 2139 | 3270 | 4396 | 5589 | 6824 | 7849 | 8294 | 8315 | 8315 |
| **General CMS** | 0.564 (0.556-0.572) | 0.557 (0.551-0.563) | 0.553 (0.548-0.558) | 0.553 (0.549-0.557) | 0.552 (0.548-0.555) | 0.551 (0.548-0.555) | 0.549 (0.546-0.552) | 0.549 (0.546-0.552) | 0.549 (0.546-0.552) | 0.549 (0.546-0.552) |
| **Mortality CMS** | 0.656 (0.648-0.664) | 0.656 (0.65-0.661) | 0.648 (0.643-0.652) | 0.646 (0.642-0.65) | 0.643 (0.64-0.646) | 0.644 (0.64-0.647) | 0.642 (0.639-0.645) | 0.641 (0.639-0.644) | 0.642 (0.639-0.644) | 0.642 (0.639-0.644) |
|  |  |  |  |  |  |  |  |  |  |  |
| **Hospital CMS** | 0.656 (0.649-0.664) | 0.656 (0.65-0.661) | 0.647 (0.643-0.652) | 0.645 (0.641-0.649) | 0.643 (0.639-0.646) | 0.643 (0.64-0.646) | 0.642 (0.639-0.644) | 0.641 (0.638-0.644) | 0.641 (0.638-0.644) | 0.641 (0.638-0.644) |
| **Unweighted Count Score** | 0.563 (0.555-0.571) | 0.556 (0.55-0.561) | 0.554 (0.549-0.558) | 0.554 (0.55-0.558) | 0.553 (0.549-0.556) | 0.552 (0.549-0.556) | 0.55 (0.547-0.553) | 0.55 (0.547-0.553) | 0.55 (0.547-0.553) | 0.55 (0.547-0.553) |

Table S4 – Discrimination (c-statistic) of the CMS (short versions of the models including only 20 conditions)

| **Primary analysis** | **Years of follow-up** | | | | | | | | | |
| --- | --- | --- | --- | --- | --- | --- | --- | --- | --- | --- |
|  | **1** | **2** | **3** | **4** | **5** | **6** | **7** | **8** | **9** | **10** |
| **Outcome: Death** | | | | | | | | | | |
| **Deaths (cumulative)** | 161 | 533 | 965 | 1464 | 1987 | 2620 | 3275 | 3937 | 4700 | 5503 |
| **Mortality CMS** | 0.665 (0.645-0.686) | 0.685 (0.675-0.696) | 0.683 (0.675-0.691) | 0.684 (0.677-0.69) | 0.684 (0.679-0.69) | 0.688 (0.683-0.693) | 0.69 (0.685-0.694) | 0.691 (0.687-0.695) | 0.694 (0.69-0.698) | 0.695 (0.692-0.699) |
| **Hospital CMS** | 0.685 (0.665-0.706) | 0.704 (0.693-0.715) | 0.701 (0.693-0.709) | 0.7 (0.693-0.706) | 0.698 (0.693-0.704) | 0.7 (0.696-0.705) | 0.702 (0.698-0.707) | 0.703 (0.699-0.707) | 0.706 (0.702-0.71) | 0.707 (0.704-0.711) |
| **Unweighted Count Score** | 0.688 (0.667-0.709) | 0.675 (0.663-0.686) | 0.667 (0.659-0.676) | 0.654 (0.647-0.661) | 0.641 (0.635-0.647) | 0.637 (0.632-0.642) | 0.636 (0.631-0.64) | 0.635 (0.631-0.64) | 0.637 (0.633-0.641) | 0.635 (0.631-0.639) |
| **Outcome: GP Consultation Rate** | | | | | | | | | | |
| **Cohort Size** | 111166 | 110083 | 108737 | 107479 | 105726 | 102777 | 58192 | 10219 |  |  |
| **Median Rate** | 6.00 | 6.50 | 7.00 | 7.00 | 7.20 | 7.33 | 7.43 | 7.63 |  |  |
| **Mortality CMS** | 0.599 (0.598-0.6) | 0.611 (0.61-0.612) | 0.618 (0.617-0.619) | 0.622 (0.621-0.622) | 0.625 (0.624-0.626) | 0.628 (0.627-0.629) | 0.63 (0.629-0.631) | 0.633 (0.63-0.636) |  |  |
| **Hospital CMS** | 0.617 (0.616-0.618) | 0.629 (0.628-0.63) | 0.636 (0.635-0.637) | 0.64 (0.639-0.641) | 0.643 (0.642-0.644) | 0.646 (0.645-0.647) | 0.648 (0.647-0.649) | 0.65 (0.647-0.653) |  |  |
| **Unweighted Count Score** | 0.646 (0.645-0.646) | 0.653 (0.653-0.654) | 0.655 (0.655-0.656) | 0.656 (0.656-0.657) | 0.657 (0.657-0.658) | 0.658 (0.657-0.659) | 0.657 (0.656-0.658) | 0.657 (0.655-0.659) |  |  |
| **Outcome: Cancer Diagnosis** | | | | | | | | | | |
| **Cases (cumulative)** | 1071 | 2139 | 3270 | 4396 | 5589 | 6824 | 7849 | 8294 | 8315 | 8315 |
| **Mortality CMS** | 0.656 (0.648-0.663) | 0.656 (0.65-0.661) | 0.648 (0.643-0.652) | 0.646 (0.642-0.649) | 0.643 (0.639-0.646) | 0.643 (0.64-0.646) | 0.642 (0.639-0.645) | 0.641 (0.638-0.644) | 0.641 (0.639-0.644) | 0.641 (0.639-0.644) |
| **Hospital CMS** | 0.656 (0.649-0.664) | 0.656 (0.65-0.661) | 0.647 (0.643-0.652) | 0.645 (0.641-0.649) | 0.642 (0.639-0.646) | 0.643 (0.64-0.646) | 0.641 (0.638-0.644) | 0.641 (0.638-0.644) | 0.641 (0.638-0.644) | 0.641 (0.638-0.644) |
| **Unweighted Count Score** | 0.561 (0.553-0.57) | 0.555 (0.549-0.561) | 0.553 (0.548-0.557) | 0.552 (0.548-0.556) | 0.55 (0.547-0.554) | 0.55 (0.547-0.553) | 0.549 (0.546-0.551) | 0.548 (0.546-0.551) | 0.548 (0.546-0.551) | 0.548 (0.546-0.551) |

Table S5– Discrimination (c-statistic) by subgroup (age, sex, age & sex) of the General CMS (long version including all 37 conditions), for the outcomes of (a) death, (b) GP consultation rate and (c) cancer diagnosis

| **A: Outcome, Death** | **Cohort Size** | **Years of follow-up** | | | | | | | | | |
| --- | --- | --- | --- | --- | --- | --- | --- | --- | --- | --- | --- |
|  |  | **1** | **2** | **3** | **4** | **5** | **6** | **7** | **8** | **9** | **10** |
| Whole Cohort | 111898 | 0.697 (0.676-0.719) | 0.69 (0.678-0.702) | 0.682 (0.673-0.691) | 0.668 (0.661-0.675) | 0.654 (0.648-0.66) | 0.649 (0.643-0.654) | 0.646 (0.642-0.651) | 0.646 (0.642-0.651) | 0.648 (0.643-0.652) | 0.645 (0.641-0.649) |
| In 40’s | 24269 | 0.705 (0.627-0.784) | 0.677 (0.632-0.722) | 0.65 (0.616-0.685) | 0.656 (0.628-0.685) | 0.631 (0.606-0.655) | 0.626 (0.605-0.647) | 0.624 (0.605-0.643) | 0.617 (0.599-0.635) | 0.62 (0.603-0.637) | 0.613 (0.596-0.629) |
| In 50’s | 36605 | 0.67 (0.623-0.717) | 0.668 (0.642-0.694) | 0.657 (0.638-0.676) | 0.645 (0.63-0.66) | 0.622 (0.609-0.636) | 0.613 (0.601-0.624) | 0.613 (0.602-0.623) | 0.613 (0.603-0.623) | 0.614 (0.605-0.623) | 0.611 (0.602-0.619) |
| In 60’s | 50425 | 0.672 (0.646-0.699) | 0.665 (0.65-0.679) | 0.659 (0.648-0.67) | 0.64 (0.631-0.649) | 0.63 (0.623-0.638) | 0.625 (0.618-0.631) | 0.622 (0.616-0.628) | 0.621 (0.616-0.627) | 0.622 (0.617-0.627) | 0.62 (0.615-0.625) |
|  | | | | | | | | | | | |
| All women | 60036 | 0.659 (0.619-0.698) | 0.66 (0.639-0.681) | 0.656 (0.641-0.672) | 0.648 (0.636-0.66) | 0.633 (0.623-0.643) | 0.628 (0.619-0.637) | 0.625 (0.617-0.633) | 0.627 (0.62-0.635) | 0.631 (0.624-0.638) | 0.628 (0.621-0.635) |
| Women, 40's | 13099 | 0.765 (0.637-0.893) | 0.625 (0.554-0.697) | 0.579 (0.523-0.635) | 0.617 (0.574-0.659) | 0.57 (0.534-0.606) | 0.554 (0.524-0.585) | 0.566 (0.538-0.594) | 0.568 (0.542-0.595) | 0.573 (0.548-0.599) | 0.565 (0.541-0.588) |
| Women, 50's | 20378 | 0.636 (0.562-0.709) | 0.641 (0.599-0.683) | 0.64 (0.611-0.669) | 0.63 (0.606-0.653) | 0.599 (0.578-0.619) | 0.597 (0.579-0.615) | 0.594 (0.578-0.611) | 0.598 (0.583-0.613) | 0.596 (0.582-0.61) | 0.594 (0.581-0.607) |
| Women, 60s | 26277 | 0.624 (0.574-0.674) | 0.643 (0.616-0.669) | 0.641 (0.622-0.661) | 0.628 (0.612-0.643) | 0.621 (0.608-0.634) | 0.616 (0.605-0.627) | 0.611 (0.601-0.621) | 0.612 (0.603-0.622) | 0.616 (0.608-0.625) | 0.613 (0.604-0.621) |
|  | | | | | | | | | | | |
| All men | 51862 | 0.714 (0.688-0.74) | 0.706 (0.692-0.721) | 0.699 (0.688-0.709) | 0.682 (0.673-0.691) | 0.669 (0.661-0.677) | 0.663 (0.656-0.67) | 0.662 (0.656-0.668) | 0.661 (0.655-0.666) | 0.661 (0.655-0.666) | 0.658 (0.653-0.663) |
| Men, 40's | 11170 | 0.689 (0.594-0.784) | 0.712 (0.656-0.768) | 0.694 (0.653-0.736) | 0.689 (0.652-0.725) | 0.68 (0.648-0.713) | 0.685 (0.656-0.713) | 0.673 (0.648-0.699) | 0.659 (0.635-0.683) | 0.66 (0.637-0.682) | 0.654 (0.632-0.675) |
| Men, 50's | 16227 | 0.697 (0.637-0.756) | 0.691 (0.658-0.723) | 0.675 (0.651-0.7) | 0.661 (0.641-0.681) | 0.644 (0.627-0.661) | 0.628 (0.613-0.644) | 0.63 (0.616-0.644) | 0.628 (0.615-0.641) | 0.631 (0.619-0.643) | 0.626 (0.615-0.638) |
| Men, 60s | 24148 | 0.686 (0.655-0.716) | 0.673 (0.656-0.691) | 0.667 (0.654-0.68) | 0.646 (0.635-0.657) | 0.635 (0.626-0.645) | 0.629 (0.621-0.638) | 0.627 (0.62-0.635) | 0.627 (0.62-0.634) | 0.625 (0.619-0.632) | 0.624 (0.618-0.63) |

| **B: Outcome, GP consultation rate** | **Cohort Size** | **Years of follow-up** | | | | | | | | | |
| --- | --- | --- | --- | --- | --- | --- | --- | --- | --- | --- | --- |
|  |  | **1** | **2** | **3** | **4** | **5** | **6** | **7** | **8** | **9** | **10** |
| Whole Cohort | 111898 | 0.654 (0.654-0.655) | 0.663 (0.662-0.664) | 0.665 (0.664-0.666) | 0.666 (0.665-0.667) | 0.667 (0.666-0.668) | 0.668 (0.667-0.669) | 0.667 (0.665-0.668) | 0.665 (0.663-0.668) |  |  |
| In 40’s | 24269 | 0.62 (0.618-0.622) | 0.626 (0.625-0.628) | 0.629 (0.627-0.631) | 0.63 (0.628-0.631) | 0.631 (0.629-0.633) | 0.631 (0.629-0.632) | 0.63 (0.628-0.632) | 0.638 (0.633-0.643) |  |  |
| In 50’s | 36605 | 0.643 (0.642-0.644) | 0.651 (0.65-0.652) | 0.654 (0.652-0.655) | 0.655 (0.653-0.656) | 0.655 (0.654-0.656) | 0.656 (0.654-0.657) | 0.654 (0.652-0.656) | 0.65 (0.645-0.655) |  |  |
| In 60’s | 50425 | 0.649 (0.648-0.651) | 0.657 (0.656-0.659) | 0.658 (0.657-0.66) | 0.66 (0.658-0.661) | 0.661 (0.659-0.662) | 0.661 (0.66-0.663) | 0.661 (0.659-0.663) | 0.656 (0.651-0.66) |  |  |
|  | | | | | | | | | | | |
| All women | 60036 | 0.647 (0.646-0.648) | 0.657 (0.656-0.658) | 0.66 (0.659-0.661) | 0.662 (0.661-0.663) | 0.664 (0.663-0.665) | 0.665 (0.664-0.666) | 0.665 (0.664-0.666) | 0.661 (0.658-0.665) |  |  |
| Women, 40's | 13099 | 0.619 (0.617-0.622) | 0.628 (0.625-0.63) | 0.631 (0.628-0.633) | 0.632 (0.63-0.635) | 0.634 (0.632-0.637) | 0.634 (0.632-0.636) | 0.636 (0.633-0.639) | 0.644 (0.637-0.651) |  |  |
| Women, 50's | 20378 | 0.641 (0.638-0.643) | 0.65 (0.648-0.652) | 0.654 (0.652-0.656) | 0.655 (0.653-0.657) | 0.656 (0.654-0.658) | 0.657 (0.656-0.659) | 0.657 (0.655-0.66) | 0.653 (0.647-0.659) |  |  |
| Women, 60s | 26277 | 0.645 (0.643-0.647) | 0.655 (0.653-0.657) | 0.657 (0.655-0.658) | 0.659 (0.657-0.661) | 0.66 (0.659-0.662) | 0.662 (0.66-0.663) | 0.661 (0.659-0.664) | 0.651 (0.645-0.658) |  |  |
|  | | | | | | | | | | | |
| All men | 51862 | 0.663 (0.662-0.664) | 0.669 (0.668-0.67) | 0.67 (0.669-0.672) | 0.671 (0.669-0.672) | 0.671 (0.67-0.672) | 0.671 (0.67-0.672) | 0.668 (0.666-0.669) | 0.67 (0.666-0.674) |  |  |
| Men, 40's | 11170 | 0.614 (0.611-0.617) | 0.617 (0.615-0.62) | 0.619 (0.617-0.622) | 0.619 (0.617-0.621) | 0.619 (0.617-0.622) | 0.619 (0.617-0.622) | 0.616 (0.613-0.619) | 0.625 (0.617-0.632) |  |  |
| Men, 50's | 16227 | 0.631 (0.629-0.634) | 0.634 (0.631-0.636) | 0.632 (0.63-0.634) | 0.631 (0.628-0.633) | 0.629 (0.626-0.631) | 0.628 (0.625-0.63) | 0.622 (0.618-0.625) | 0.621 (0.613-0.629) |  |  |
| Men, 60s | 24148 | 0.654 (0.652-0.656) | 0.66 (0.658-0.662) | 0.66 (0.658-0.662) | 0.66 (0.658-0.662) | 0.661 (0.659-0.663) | 0.661 (0.659-0.663) | 0.661 (0.659-0.664) | 0.66 (0.654-0.667) |  |  |

| **C: Outcome, Cancer Diagnosis** | **Cohort Size** | **Years of follow-up** | | | | | | | | | |
| --- | --- | --- | --- | --- | --- | --- | --- | --- | --- | --- | --- |
|  |  | **1** | **2** | **3** | **4** | **5** | **6** | **7** | **8** | **9** | **10** |
| Whole Cohort | 111898 | 0.564 (0.556-0.572) | 0.557 (0.551-0.563) | 0.553 (0.548-0.558) | 0.553 (0.549-0.557) | 0.552 (0.548-0.555) | 0.551 (0.548-0.555) | 0.549 (0.546-0.552) | 0.549 (0.546-0.552) | 0.549 (0.546-0.552) | 0.549 (0.546-0.552) |
| In 40’s | 24269 | 0.522 (0.496-0.547) | 0.5 (0.482-0.518) | 0.52 (0.506-0.535) | 0.513 (0.501-0.525) | 0.515 (0.504-0.526) | 0.517 (0.507-0.527) | 0.511 (0.502-0.52) | 0.51 (0.501-0.518) | 0.51 (0.501-0.518) | 0.51 (0.501-0.518) |
| In 50’s | 36605 | 0.528 (0.511-0.545) | 0.52 (0.508-0.531) | 0.511 (0.502-0.521) | 0.51 (0.502-0.518) | 0.509 (0.502-0.516) | 0.512 (0.506-0.519) | 0.513 (0.508-0.519) | 0.513 (0.508-0.519) | 0.513 (0.508-0.519) | 0.513 (0.508-0.519) |
| In 60’s | 50425 | 0.54 (0.53-0.55) | 0.536 (0.529-0.543) | 0.532 (0.527-0.538) | 0.535 (0.53-0.54) | 0.533 (0.529-0.538) | 0.531 (0.527-0.535) | 0.529 (0.525-0.533) | 0.529 (0.525-0.533) | 0.529 (0.525-0.532) | 0.529 (0.525-0.532) |
|  | | | | | | | | | | | |
| All women | 60036 | 0.536 (0.524-0.548) | 0.537 (0.529-0.545) | 0.539 (0.532-0.546) | 0.541 (0.535-0.547) | 0.541 (0.536-0.546) | 0.539 (0.535-0.544) | 0.539 (0.535-0.543) | 0.539 (0.535-0.543) | 0.539 (0.535-0.543) | 0.539 (0.535-0.543) |
| Women, 40's | 13099 | 0.515 (0.485-0.546) | 0.502 (0.48-0.524) | 0.526 (0.509-0.544) | 0.508 (0.493-0.522) | 0.51 (0.498-0.523) | 0.508 (0.496-0.519) | 0.498 (0.487-0.509) | 0.497 (0.486-0.508) | 0.497 (0.486-0.508) | 0.497 (0.486-0.508) |
| Women, 50's | 20378 | 0.491 (0.469-0.513) | 0.501 (0.485-0.516) | 0.498 (0.486-0.51) | 0.501 (0.49-0.511) | 0.504 (0.495-0.513) | 0.506 (0.497-0.514) | 0.51 (0.502-0.518) | 0.511 (0.503-0.519) | 0.511 (0.503-0.519) | 0.511 (0.503-0.519) |
| Women, 60s | 26277 | 0.532 (0.516-0.548) | 0.533 (0.522-0.544) | 0.534 (0.525-0.543) | 0.542 (0.534-0.55) | 0.541 (0.534-0.547) | 0.538 (0.531-0.544) | 0.537 (0.531-0.543) | 0.537 (0.531-0.543) | 0.537 (0.531-0.543) | 0.537 (0.531-0.543) |
|  | | | | | | | | | | | |
| All men | 51862 | 0.592 (0.58-0.603) | 0.577 (0.569-0.585) | 0.569 (0.562-0.575) | 0.567 (0.561-0.572) | 0.564 (0.559-0.569) | 0.564 (0.56-0.569) | 0.561 (0.557-0.565) | 0.56 (0.556-0.565) | 0.56 (0.556-0.564) | 0.56 (0.556-0.564) |
| Men, 40's | 11170 | 0.512 (0.469-0.556) | 0.466 (0.436-0.495) | 0.471 (0.447-0.495) | 0.496 (0.474-0.518) | 0.494 (0.475-0.514) | 0.509 (0.491-0.528) | 0.512 (0.495-0.529) | 0.511 (0.494-0.527) | 0.511 (0.494-0.527) | 0.511 (0.494-0.527) |
| Men, 50's | 16227 | 0.576 (0.549-0.603) | 0.545 (0.527-0.563) | 0.529 (0.515-0.543) | 0.521 (0.509-0.533) | 0.515 (0.505-0.526) | 0.52 (0.511-0.53) | 0.517 (0.508-0.526) | 0.516 (0.507-0.524) | 0.516 (0.507-0.525) | 0.516 (0.507-0.525) |
| Men, 60s | 24148 | 0.546 (0.532-0.559) | 0.538 (0.529-0.548) | 0.531 (0.523-0.539) | 0.53 (0.523-0.537) | 0.528 (0.522-0.534) | 0.526 (0.52-0.531) | 0.522 (0.517-0.528) | 0.522 (0.517-0.527) | 0.522 (0.517-0.527) | 0.522 (0.517-0.527) |

Table S6 - Comparison of discrimination (c-statistic) between development study (as reported by Payne et al.) and validation in UK Biobank, for a 1-year follow-up (all values are given for the long versions - including all 37 conditions - of the CMS)

| **a: Validation (UKB)** | **Outcomes (1 -year)** | | |
| --- | --- | --- | --- |
|  | **Death** | **Cancer Diagnosis** | **Rate** |
| **General CMS** | 0.697 (0.676-0.719) | 0.564 (0.556-0.572) | 0.654 (0.654-0.655) |
| **Mortality CMS** | 0.666 (0.646-0.687) | 0.656 (0.648-0.664) | 0.599 (0.598-0.6) |
| **Hospital CMS** | 0.688 (0.668-0.708) | 0.656 (0.649-0.664) | 0.617 (0.616-0.618) |
| **Unweighted Count Score** | 0.666 (0.646-0.687) | 0.563 (0.555-0.571) | 0.655 (0.654-0.656) |
|  | | | |
| **b: Development (CPRD), from Payne et al. (2020)** | **Outcomes (1- year)** | | |
|  | **Death** | **Cancer Diagnosis** | **Rate** |
| **General CMS** | 0.880 (0.872–0.889) | **-** | 0.690 (0.689–0.691) |
| **Mortality CMS** | 0.908 (0.902-0.915) | - | - |
| **Hospital CMS** | - | - | - |
| **Unweighted Count Score** | - | - | - |

Table S7 – UK Biobank data providers, number of participants, last linkage date and coding framework

| **Ref** | **GP Computer System Suppliers** | **Number of UK Biobank participants included** | **Region** | **Clinical Coding Framework** | **Prescription Coding Framework** | **Last reliable linkage date** | **% UK population covered by data provider** |
| --- | --- | --- | --- | --- | --- | --- | --- |
| 1 | Vision | 18,000 | England | Readv2 | Readv2/DM+D | 25.05.2017 | 18.1% (English) |
| 2 | EMIS/Vision | 27,000 | Scotland | Readv2 | Readv2/BNF (6 char) | 19.04.2017 | 100% (Scottish) |
| 3 | TPP (SystmOne) | 165,000 | England | CTV3 | BNF (10 char) | 14.06.2016 | 17.8% (English) |
| 4 | EMIS/Vision | 21,000 | Wales | Readv2 | Readv2 | 18.11.2017 | 100% (Welsh) |

*Information drawn from* [*https://biobank.ndph.ox.ac.uk/showcase/showcase/docs/primary_care_data.pdf*](https://biobank.ndph.ox.ac.uk/showcase/showcase/docs/primary_care_data.pdf) *more details can be found in the linked document provided by UK Biobank*
